# Supplementary material for: An experimentally supported model of the Bacillus subtilis global transcriptional regulatory network
Source: Mol Syst Biol. 2015 Nov 17;11(11):839. doi: 10.15252/msb.20156236 (PMC4670728; doi:10.15252/msb.20156236)
Supplement: Supplementary file 1 — Appendix [file MSB-11-839-s001.pdf]

APPENDIX

TABLE OF CONTENTS

MATERIAL AND METHODS

Bacterial strains and primers (p.3)

Transcriptional Profiling Experiments (pp.3-5)

Media and growth conditions

RNA isolation, cDNA synthesis, labeling and hybridization to microarrays

Microarray design

Validation experiments (other than transcriptional profiling) (pp.5-6)

Fluorescence microscopy

Spore purification procedure

Computational search for DNA binding motifs

Network inference (pp.6-9)

Gold standard (GS) network

Inferring regulatory relationships using Bayesian Best Subset Regression

Bayesian Regression With Informative Prior

Model selection

Comparing TFA-BBSR to other inference methods

Network visualization and exploration (pp.10-11)

Network visualization

Network modularity

Functional enrichment analyses

Differential gene transcription analyses

Network validation using gene counts

RESULTS

Network validation experiments (pp.11-13)

Transcriptional profiling experiments to characterize the regulons of alternative σ factors

Other regulators

External validation

DNA motifs

Functional analyses (pp.14-16)

Competence (ComK regulon)

Metabolism (CodY regulon)

Sporulation

REFERENCES (pp. 17-19)

APPENDIX TABLES (pp.20-21)

APPENDIX FIGURES (p.22-30)

MATERIAL AND METHODS

Bacterial strains and primers

All *B. subtilis* strains used are listed in **Table EV1**. Gene fusions to *gfp* were generated by cloning the 3' end of the target gene (amplified by PCR from genomic DNA) into pCV0119 plasmid (van Ooij *et al*, 2004). Primers used for production of *gfp* fusions are listed below:

YtdA-left: GCGCGGATCCgtgccgctgatagggttaaa
YtdA-right: GCGCCTCGAGtgggggttcttttccgtttt
SpsI-left: GCGCGGATCCcagaacgctttgaacagcag
SpsI-right: GCGCCTCGAGcttttcgtcctgtcctttcc
SpsJ-left: gcgcGGATCCgccatcattacacgatgcag
SpsJ-right: gcgcCTCGAGtcggtcattgtctgtataccactg
SpsK-left: gatcGGATCCacaaaagacagc
SpsK-right: gatcCTCGAGatcacacgcactgctcc
YtcB-left: GGATCCaagccgcttaccat
YtcB-right: CTCGAGttccccctgatacagcga
YfnH-left: GCGCGGATCCcgtttttggagactggggta
YfnH-right: GCGCCTCGAGccataccttccatacttgttgtcc
SpsM-left: gatcGGATCCgacgatgcagctac
SpsM-right: gatcCTCGAGtgcctttttctcttg
YetF-left: GCGCGGATCCgcaaagcctattgaggcaag
YetF-right: GCGCCTCGAGtgttgatctgctttgttcgtacg
YkoT-left: GCGCGGATCCtgggtggacggtacattttt
YkoT-right: GCGCCTCGAGactgtactttttttctaaccgctc
YkzQ-left: GCGCGGATCCtgaatgaaggggaaaatacct
YkzQ-right: GCGCCTCGAGtctttctcttatctttataatttgtcc

Transcriptional Profiling Experiments

Experimental conditions for every transcriptional profiling experiment are provided in **Table EV2**.

Media and growth conditions

The following media were used: Luria-Bertani (per liter: 10g Tryptone, 5g Yeast extract, and 10g NaCl); Difco Sporulation Medium (per liter: 8g Bacto-nutrient broth, 10mL 10% (w/v) KCl, 10mL 1.2% (w/v) $MgSO_4 \cdot 7H_2O$, 0.5mL NaOH, 1.0 mL 1M $Ca(NO_3)_2$, 1.0mL 0.1M $MnCl_2$, and 1.0mL 1mM $FeSO_4$); Supplemented Lysogeny Broth (per liter: 2.5g Tryptone, 1.2g Yeast extract, 2.5g NaCl, 50 mL CHIII [1.98g $MgSO_4 \cdot 7H_20$ and 4mL 10% $CaCl_2$ per 100mL], and 2mL CHIV [1.1g $MnSO_4 \cdot 4H_20$ per 100 mL]); Sterlini Mandelstam medium (90mL Solution A+B [per liter: 989mL H_2O, 1mL Solution A {0.089g $FeCl_3 \cdot 6H_2O$, 0.830g $MgCl_2 \cdot 6H_2O$, and 1.979g $MnCl_2 \cdot 4H_2O$ per 100mL}, and 10 mL Solution B {53.5g NH_4Cl, 10.6g Na_2SO_4, 6.8g KH_2PO_4, and 9.7g NH_4NO_3 per 100mL} with pH adjusted to 7.0 using 2M NaOH], 4mL 5% glutamate, 1mL 0.1M $CaCl_2$, and 4mL 1M $MgSO_4$); Competence medium (To 500mL 1X Spizizen salts (per liter: 6g KH_2PO_4, 14g K_2HPO_4, 2g $(NH_4)_2SO_4$, 1g Na-citrate·$2H_2O$, 0.2g $MgSO_4 \cdot 7H_2O$) add 5mL 10% yeast extract, 5mL 2% casein hydrolyzate, 5mL 50% glucose, 2.5mL 10mg/10mL

histidine, 2.5mL 10mg/mL leucine, 2.5mL 10mg/mL methionine, and 2.5mL 0.5M $MgCl_2$); MSgg medium (5mM KH_2PO_4 (pH 7), 100mM MOPS (pH 7), 2mM $MgCl_2$, 700μM $CaCl_2$, 50μM $MnCl_2$, 50μM $FeCl_3$, 1μM $ZnCl_2$, 2μM thiamine, 0.5% glycerol, 0.5% glutamate). The phosphate minimal medium has been described in Pragai & Hardwood (2000). Unless indicated otherwise, bacterial cultures were grown at 37°C. For sporulation by resuspension, cells were incubated at 37°C in supplemented lysogeny broth medium to an A_{600} of 0.6, pelleted by centrifugation at 4000 *g* for 5 min and resuspended in SM medium (Sterlini & Mandelstam, 1969).

RNA isolation, cDNA synthesis, labeling and hybridization to microarrays

Samples were immediately mixed with an equal volume of methanol (-20˚C) and centrifuged to pellet cells. Cell pellets were stored at -80˚C. RNA was isolated using a hot acid-phenol isolation procedure modified from Fawcett et al (2000). In this method, cell pellets are resuspended in LETS Buffer (10 mM Tris-HCl pH 8.0, 50 mM LiCl, 10 mM EDTA, 1% SDS) and then vortexed with acid-washed beads and hot (75˚C) acid-phenol, followed by addition of chloroform. The aqueous phase is next treated with hot phenol-chloroform and the subsequent aqueous phase is added to an equal volume of isopropanol to precipitate the RNA. The sample is centrifuged, washed, and resuspended in water. The sample is then treated with Qiagen's RNase-Free DNase in solution and the RNA clean-up protocol of Qiagen's RNeasy® kit was used. The quality of the RNA was checked by visualizing the integrity of the 23S and 16S rRNA bands on an agarose gel. The RNA was quantified using the nanodrop.

Labeled cDNA was generated from RNA using Agilent's Fairplay® III Microarray Labeling Kit. The Agilent Two-Color Microarray-Based Prokaryotic Analysis (FairPlay III Labeling) Protocol was used with the following changes: between 6-10 μg total RNA was used, the reverse transcription reaction was performed at 42˚C for 2 hours, and the NHS-ester dye-coupling reaction to amino allyl dUTP (using GE Healthcare Cy™3 and Cy™5 monofunctional reactive dyes) was incubated for 90 minutes. Hybridizations were incubated at 65˚C for 17 hours. Arrays were scanned by Agilent Technologies DNA Microarray Scanner with Surescan High-Resolution Technology.

Microarray design

The NCBI and EMBL annotations of the *B. subtilis* 168 genome were downloaded on July 28th 2010 and all annotated protein coding genes and non-coding RNAs were combined with small RNAs (sRNAs) identified in the tiling array and RNAseq transcriptional profiling experiments carried out by Rasmussen et al (2009) and Irnov et

al (2010). All sequences were then combined using CD-HIT (Huang *et al*, 2010). CD-HIT was used to identify proteins that were 99% sequence identical over 99% of their length (primarily accounting for the large overlap between the NCBI and EMBL annotations). Agilent's eArray software (accessed via the Agilent website) was then used to design an array with three 60-mer probes (features) per protein-coding gene and 2 or 3 probes per RNA gene. We also incorporated 536 Agilent control features that are part of the Agilent platform. In total, the array has 15744 features for a total of 4,231 protein-coding genes and 659 putative non-coding RNAs (12 rRNAs, 49 tRNAs and 598 putative or previously characterized small RNAs) and can be ordered from Agilent (array name: b.subt-final-sense-3probes-july29).

Validation experiments (other than transcriptional profiling)

Fluorescence microscopy

Fluorescence microscopy was performed as described before (Kim *et al*, 2006; Wang *et al*, 2009; McKenney *et al*, 2010). Briefly, 1 ml aliquots of the sporulating cultures were transferred into microcentrifuge tubes and spun down at 8000 rpm for 2 min in a microcentrifuge. Pellets were resuspended in 100 µl PBS supplemented with the membrane dye FM4-64 (Invitrogen) at 1.5 µg ml^{-1} final concentration. Two microliters of the concentrated sample were placed on a microscope slide and covered by a poly-L-lysine-treated coverslip for analysis. Images were taken using a Nikon 90i motorized fluorescent microscope equipped with a Roper 1 K monochrome digital camera and driven by the NIS-Elements AR 3.0 software. A 100× Plan Fluor 1.3NA objective (Nikon) was used for all image collection. Images were processed with ImageJ (http://rsbweb.nih.gov/ij/) or Photoshop for minor adjustments of brightness, contrast and color balance.

Spore purification procedure

Bacterial cultures (20 mL) were incubated in DSM for 48 hours and centrifuged at 5,000RPM for 10 minutes at room temperature. The pellet is washed three times with 20mL cold deionized water. In a separate Eppendorf tube, 200uL 50% renografin solution is added. The pellets are re-suspended in 400uL 20% renografin solution and transferred into the Eppendorf tube, layering carefully to avoid mixing of the two solutions. The Eppendorf tube is then centrifuged at 13,200RPM for 10 minutes at room temperature. The renografin solutions with different densities help filter out cellular debris, leaving purified spores pelleted at the bottom in the 50% renografin layer. Over time, the renografin may induce germination of the spores, so the pellet is washed three times with cold deionized water in order to remove all excess renografin. The purified spores are stored in 1mL

PBS at -20°C. To test the adhesion of the spores to glass, purified spores are re-suspended in water to a final OD600 of 15. One hundred microliters of each sample are then transferred into Pyrex glass tubes and vortexed gently for 1 minute, giving the spinning spores sufficient time to adhere to the walls of the tube. The remaining solution is then aspirated off, leaving the residue behind to dry for ten minutes before image acquisition with a digital camera.

Computational search for DNA binding motifs

We used the MEME package version 4.9.1 (Bailey *et al*, 2009) to identify putative DNA sequence motifs characteristic of TF binding. For TFs with KO data, we manually defined the differentially expressed operons, i.e. when adjacent genes oriented in the same direction were differentially transcribed we grouped them in an operon, the most upstream of the differentially expressed genes was selected as the first gene in the operon. For TFs without KO data, we used the operon predictions from the MicrobesOnline database (Dehal *et al*, 2010). Subsequently, we extracted the DNA sequence from 0 to 200 bp upstream of the translational start site of the first gene in each operon predicted as target. MEME was run on each group of sequences using the zoops mode (zero or one occurrence per sequence), restricting the number of output motifs to 10 and using all other parameters as default. Next, we compared the retrieved sequence motifs with those described in previous reports (when available). For TFs for which known motifs were not fully recovered, we tried several combinations of minimum and maximum width parameters based on previously associated motifs (this was required for σ^G, σ^H and σ^K). An operon was considered to be a “known” target if any of its members was already part of the corresponding regulon in the prior network.

Network inference

Gold standard (GS) network

In total, 3040 experimentally validated transcriptional interactions of *B. subtilis*, involving 1874 genes, were obtained from a slightly larger set downloaded from the SubtiWiki database (Michna *et al*, 2014) on March 26, 2013, and from a list of σ^A-controlled genes obtained from Helmann (1995). Genes filtered out during microarray processing due to a lack of expression variance were excluded from the GS. The GS network contains 1746 positive and 1294 negative interactions (**Dataset EV2**). There are 46 TFs with more than 10 targets, 32 TFs with more than 5 but less than 11 targets, and 75 TFs with less than 6 targets.

Inferring regulatory relationships using Bayesian Best Subset Regression

We now describe the BBSR method, an inference method that computes the regression models for a given gene i corresponding to the inclusion and exclusion of each TF that has a known regulatory effect on i, and the ten TFs with highest time-lagged CLR. Prior knowledge is incorporated by using informative priors for the regression parameters, and sparsity is enforced by a model selection step based on the Bayesian Information Criterion (BIC).

Bayesian Regression With Informative Prior

Here we introduce the linear regression we use during the model building step of the algorithm. We assume the prediction error

$$\epsilon_i = y_i - \hat{A}'\beta_i$$

to be independent and identically distributed with mean 0 and variance σ^2. The response variable of gene *i* is denoted as y_i (the ith row of X), the design variables of TFs (TF activities, including time shift if applicable) as $\hat{A}$ and the regression solution as β_i. For clarity, we will omit the index *i* for the remainder of this section. We assume that the target gene response is distributed according to a multivariate normal

$$\left(y \mid \beta, \sigma^2, \hat{A}\right) \propto N_n\left(\hat{A}'\beta, \sigma^2 I\right)$$

with the predicted response as mean, and a variance co-variance matrix that has the error variance σ^2 on its diagonal and is 0 otherwise. In this formulation, *n* is the number of observations (experiments). This can be solved by a Bayesian regression where we can incorporate existing knowledge by tuning the prior on β.

We use a modification of Zellner's g Prior (Zellner, 1983) to include subjective information in our Bayesian regression problem. In the original formulation, the prior distribution of β has the following form

$$\rho\left(\beta \mid \sigma^2\right) \propto N\left(\beta^0, g\left(\hat{A}\hat{A}'\right)^{-1}\sigma^2\right),$$

i.e. a distribution proportional to a multivariate normal with an initial guess β^0 as mean and a data-dependent covariance matrix that is scaled by a user chosen factor of $g \in (0, \infty)$. The prior distribution of σ^2 is the same as

is typically used with the non-informative prior, $\rho\left(\sigma^2\right) \propto \frac{1}{\sigma^2}$. The choice of a large value for *g* will lead to results centered around the ordinary least squares solution, and the error variance will be the lowest. Values of *g* close to 0 on the other hand will lead to solutions that are centered around β^0 with higher error variance.

The joint posterior distribution has the functional form

$$\rho\left(\beta,\sigma^2|y\right)=\rho\left(\beta|y,\sigma^2\right)\rho\left(\sigma^2|y\right),$$

and the marginal posterior distributions are

$$\rho\left(\beta|y,\sigma^2\right)\propto N\left(\frac{g}{g+1}\left(\frac{\beta^0}{g}+\beta^{ols}\right),\frac{\sigma^2 g}{g+1}\left(\hat{A}\hat{A}'\right)^{-1}\right),$$

$$\rho\left(\sigma^2|y\right)\propto \mathrm{IG}\left(\frac{n}{2},\frac{\mathrm{SSR}}{2}+\frac{\left(\beta^0-\beta^{\mathrm{ols}}\right)\hat{A}\hat{A}'\frac{1}{g+1}\left(\beta^0-\beta^{\mathrm{ols}}\right)}{2}\right),$$

where IG is the Inverse Gamma distribution with shape and scale parameter, and SSR is the sum of squares of the residuals of the ordinary least squares solution β^{ols}.

With this set-up, we can propose a prior guess β^0 of the vector of regression coefficients, and encode our belief in this guess with g. To allow for different levels of confidence in the different elements of β^0 we extend the original formulation of the g prior to use a vector $\bar{g}$ with one entry per predictor. The scale parameter of the Inverse Gamma distribution of the marginal posterior distribution of σ^2 then becomes

$$\mathrm{scale}=\frac{\mathrm{SSR}}{2}+\frac{\left(\beta^0-\beta^{\mathrm{ols}}\right)G\hat{A}\hat{A}'G\left(\beta^0-\beta^{\mathrm{ols}}\right)}{2},$$

where *G* is a square diagonal matrix whose diagonal entries starting in the upper left corner are $\sqrt{\frac{1}{\bar{g}+1}}$ and all remaining entries are 0.

In practice, we choose β^0 to be a vector with all entries having the value 0. This reflects our prior belief that the regulatory network is generally quite sparse. We set the vector $\bar{g}$ to values of *g* for those predictors that we have additional knowledge for and believe that they regulate gene *i*, and to 1/*g* for the other predictors. A value of *g* = 1 treats all predictors equally and we refer to it as 'no priors', whereas *g* > 1 allows the predictors with priors to explain for more of the variance of the response.

Model selection

We use the BIC to select the final model from the 2^p possible regression models for a gene *i*. For a given model *m*, the BIC is defined as

$$\mathrm{BIC}_m=n\ln\left(\sigma^2\right)+k\ln\left(n\right)$$

where n is the number of observations and k the number of predictors. To be more robust, we avoid using a point estimator for σ^2 directly, but use the expected value of BIC_m based on the posterior distribution of σ^2

$$E\left[\mathrm{BIC}_m\right]=nE\left[\ln\left(\sigma^2\right)\right]+k\ln\left(n\right)$$

$$E\left[\mathrm{BIC}_m\right]=n\left(\ln\left(shape\right)-\mathrm{Digamma}\left(scale\right)\right)+k\ln\left(n\right),$$

where *shape* and *scale* parameterize the marginal posterior distribution of σ^2 as stated above. As a final step, the predictors of the model with the lowest E[BIC] are selected as the TFs regulating gene *i*.

If *p*, the total number of potential predictors for a given gene, is large (> 10) it becomes infeasible to compute all 2^p possible regression models during the model selection step. To further reduce the number of predictors, we look at a subset of all possible models, and employ an averaging method to discover the 10 most promising predictors. We first build all models containing one or two predictors, and compute the expected BIC for each one. For every predictor we compute the average expected BIC of all models containing that particular predictor. These averages allow us to rank the predictors, and to reduce the set to the 10 best predictors as defined by the BIC in this small subspace of all models.

Comparing TFA-BBSR to other inference methods

To better assess the performance of our method with respect to other state of the art network inference algorithms, we created networks using three additional methods: 1) ARACNE (Margolin *et al*, 2006), 2) CLR (Faith *et al*, 2007), 3) GENIE3 (Huynh-Thu *et al*, 2010). We implemented CLR using the mutual information estimates as provided by the parmigene R package (Sales & Romualdi, 2011). We obtained GENIE3 from http://homepages.inf.ed.ac.uk/vhuynht/software.html and used the R implementation with default parameters. All methods were tested using both data sets, and we modified CLR and GENIE3 to be able to supply design and response variables while keeping the core algorithms the same. This way, all three methods are given identical input (as defined by the TFA estimation and Inferelator core model) and differences due to the use of time-series information are eliminated. Additionally, we obtained ARACNE from http://wiki.c2b2.columbia.edu/califanolab/index.php/Software/ARACNE (aracne2 executable) and ran it using 66 combinations of different MI p-value and DPI tolerance parameters. P-value parameters where in the set of $1e^{-10}$, $1e^{-9}$, $1e^{-8}$, …, 1, while DPI tolerance parameters where in the set 0, 0.05, 0.1, 0.15, 0.2, 1. We observed the highest performance at p-value parameter $1e^{-10}$ and DPI tolerance parameter 0 for which we performed a final

run doing 50 bootstraps. As the performance (measured by AUPR) was only about 50% (0.076) of that of the other tested methods, we did not pursue ARACNE any further.

Network visualization and exploration

Network visualization

The combined network was visualized using Cytoscape 2.8 (Smoot *et al*, 2011). The multiColoredNodes plug-in (Warsow *et al*, 2010) was used to visualize information about the proportion of recovered priors and novel targets for each regulon.

Network modularity

The inferred TRN was clustered (**Figure 5**) using the community cluster algorithm (GLay) with the ClusterMaker plug-in (Morris *et al*, 2011). If at least half of the members of a module are associated with a specific cellular process (as defined by the SubtiWiki gene annotation: http://subtiwiki.uni-goettingen.de/wiki/index.php/Categories) that process becomes the main identifier of the module. When TFs are connected to more than one module, we verify that they reside in the module with the strongest interaction based on the magnitude of the corresponding β scores. This applies to ComK (along with GlcR *and* HxlR, two TFs predicted to function downstream of ComK), which was originally in the module containing PadR, AlsR, YmfC, YofA and YcbG. We moved ComK to the 'transition' module, because the known interaction of *abrB* with *comK* is stronger (β=-0.42) than with *yofA* (β=0.19), which is a novel prediction not supported in the KO experiments. Similarly, SigW was originally part of the module containing PadR, AlsR, YmfC, YofA and YcbG. However, SigW, YbfP and FatR were moved to the 'cell envelope stress' module due to the connection of *fatR* to *sigM* and *sigW* to *yvrHb*. In the case of CodY, we chose to place the TF at an intermediate location between the 'sporulation' and 'cell envelope stress' modules, because the TF is linked with very similar β scores [*bkdR* (β=0.23) and *sigF* (β=0.24)] to TFs in both modules. Further modifications were made to the TF network figure: 1) the 'cell envelope stress' module was obtained by merging two smaller modules: one that included Abh, YwbI, YybA, YvrI, YvrHb, FadR and SigX; another with SigM, CcpN, YtlI, CymR, BkdR, WalR, HrcA, AhrC, LicR, FatR, YbfP and SigW; 2) we split a larger original module between 'general stress' and 'respiration'.

Functional enrichment analyses

Gene annotations were retrieved from SubtiWiki and available literature reports. Genes associated with specific cellular processes were identified using gene categories defined in SubtiWiki (see above).

Differential gene transcription analyses

We compared gene transcription profiles of wild-type (WT) and the respective mutant strains (KO) using Bayesian t-tests with the Cyber-T tool (Baldi & Long, 2001). The main challenge we faced with the Bayesian t-tests was the low number of differentially transcribed genes detected for several TFs after correcting for multiple hypothesis testing. This issue was also observed when other tools such as Limma (Smyth, 2004) were used. To evaluate if the small number of detected differentially transcribed genes was due to the lack of change in the transcription profile of expected genes (based on the regulon's information derived from the GS network), we computed the Precision Recall plot for each WT and KO comparison. We ranked the genes using the raw p-values obtained from the corresponding Bayesian t-test (in increasing order) and computed the Area Under Precision Recall (AUPR) curve using the TF targets in the GS network as reference. To get the expected distribution of AUPRs for the case where the arrays were uninformative with respect to the expected set of differentially transcribed genes, we also computed the AUPR using multiple random rankings of genes. We used 0.01 as the threshold p-value for considering a gene differentially transcribed.

Network validation using gene counts

To test each TF-gene interaction we used the differential transcription analyses described above. Target genes were considered to be supported when differentially transcribed (for steady state experiments) or in at least one time point for time series. All tested networks consisted of the top 4516 predicted interactions of the respective methods.

RESULTS

Network validation experiments

Transcriptional profiling experiments to characterize the regulons of alternative σ factors

In this section, we present a detailed analysis of the network predictions for the alternative σ factors and their validation by transcriptional profiling. From the data presented in **Table I and Dataset EV6**, three groups emerge based on accuracy of predictions. In the first group (high accuracy), composed of σ^E, σ^F, σ^G and σ^W, the accuracy for novel predictions was 0.8 or higher, even though compartmentalization of σ factor activity during sporulation (which applies to σ^F, σ^G, σ^E and σ^K) is a confounding factor. For instance, σ^F and σ^E are both active during the

early stages of sporulation, but σ^F activity is limited to the forespore, while σ^E controls mother cell gene transcription. Out of the 9 novel predictions that were not supported in *sigE* KO experiments, 2 were found to be σ^F-controlled targets in *sigF* KO experiments. Similarly, out of the 4 novel predictions that were not supported for σ^F in *sigF* KO experiments, 3 were differentially expressed in the *sigE* KO experiment. In most cases, inaccurate predictions can be traced back to another TF in the TRN model that is directly connected to the factor for which the prediction was made. Out of the 7 novel predictions that were not supported in the *sigE* KO experiment, and were not differentially expressed in *sigF* KO, 3 are targets of Spo0A, which controls expression of *sigE*.

In the second group (medium accuracy), σ^B, σ^D, σ^H and σ^K have accuracies for novel predictions equal to 0.57, 0.3, 0.61 and 0.62, respectively. It should be noted that with a less stringent p-value cut-off (0.05 instead of 0.01), the accuracy for novel predictions would increase significantly (to 0.86, 0.6, 0.81 and 0.74, respectively), suggesting that we might be underestimating the number of true predictions, but additional experiments would be necessary to fully validate these putative new targets. It is important to note that many of the targets of σ^K are also dependent on GerE (σ^K and GerE form a feed-forward loop). When the contribution of GerE is taken into account, the accuracy of predictions for σ^K is 0.94.

The last group (very low accuracy) consists of just one σ factor, σ^M (0.04). This is not caused by an inability to estimate activity, because 31 transcription units controlled by σ^M are present in the GS and 84% of these operons were recovered in the model. We believe that the group of putative σ^M targets contributed by the second dataset is made of co-regulated genes, but that the inference approach did not assign the correct TF(s) to it.

Other regulators

For other global regulators, accuracy for novel predictions was between 0.3 and 0.65 (**Table I**). One exception was repression by Spo0A, where the low accuracy (0.17) was likely a consequence of a noisy set of priors. The Spo0A genes in the GS had originally been identified as targets using a ChIP-on-chip approach (Molle *et al*, 2003) that lacked the precision of current approaches. While it is possible that our model could be improved by filtering priors for every TF; we do think that, overall, only very few predictions would change. Novel targets of

ScoC were also predicted at low accuracy (2 validated operons out of 9 novel predictions); however, ScoC is only one of many regulators of the transition to stationary phase. Several of the new predictions for ScoC were shown to be differentially expressed in experiments with *codY* or *spo0A* deletion mutants (which are also involved in this transition).

External validation

In addition to our KO validation experiments, we relied on recent reports of regulon characterization published after the compilation of the GS. This allowed us to confirm even more of the novel predictions, as updated regulons were recently reported for AbrB, CcpA, CodY, PhoP, Spx, TnrA, WalR and Zur. Specifically, 7 out of 17 predicted novel target operons of Spx were identified as direct targets in chromatin immunoprecipitation (ChIP) experiments by Rochat et al (2012). All 4 recovered priors and 1 out of 3 predicted novel target operons of Zur were supported by ChIP experiments (Prestel *et al*, 2015). In addition, 9 out of the 20 novel target operons we predicted for CcpA were reported as differentially expressed in strains producing CcpA at three different levels (Marciniak *et al*, 2012). For the CodY regulon, we relied on information derived from two studies, one reporting the transcriptional profiles of various *codY* mutants (Brinsmade *et al*, 2014), another describing the genome-wide identification of CodY-binding sites at single nucleotide resolution (Belitsky & Sonenshein, 2013). We integrated this information with our *in silico* analysis (see below) of the presence of putative DNA binding motifs for CodY in the upstream sequence of predicted targets (**Appendix Figure S5**) and our own transcriptional profiling experiments with a *codY* gene deletion mutant (**Table I** and **Dataset EV6**). Twelve out of 17 novel targets predicted for CodY were supported by these approaches. In general, transcriptional profiling experiments, bioinformatics analyses and external sources suggest an accuracy of at least 0.5 for novel predictions. Accuracy is expected to be greater for global regulators than local regulators, due to the fact that the number of priors is higher for these TFs and their activity can be more accurately predicted.

DNA motifs

In addition to CodY targets, we obtained conserved motifs matching previously known consensus sequences for σ^B, σ^D, σ^E, σ^G, σ^H, σ^K, σ^L, σ^W and CcpA (**Appendix Figure S5**). The proportion of recovered and novel targets exhibiting putative binding sites does not significantly differ (although it is usually slightly lower for novel targets). The highest proportion of novel targets containing a match to the consensus is obtained for σ^E (0.98). Known motifs were also identified for CymR, Fur, LexA, Zur and IolR (data not shown). Overall, when DNA binding motifs were identified, 68% of the novel targets were supported; in total, this represents 209 novel TF-operons

interactions.

Functional analyses

Competence (ComK regulon)

From the inferred TRN, we obtained a list of putative targets of ComK. Subsequently, we obtained experimental support for these interations by transcriptional profiling of a *comK* mutant strain. We grouped and color-coded these genes according to their functional annotation (**Appendix Figure S6**). As expected, the main categories were competence, DNA repair and recombination. New targets of ComK include *coiA* (*yjbF*), a gene whose role in competence had been previously recognized (Kramer *et al*, 2007), *sacY* which is involved in sucrose metabolism (Crutz & Steinmetz, 1992) and *hxlR*, which encodes a TF regulating the response to formaldehyde induced-stress (Yasueda *et al*, 1999). Another group is formed by genes encoding transporters (i.e. the *ywfM* and *ycKBA* operons, and the *yvrPON* operon, a previously known target). Thus, cellular functions of the ComK regulon are not restricted to competence, but impact a variety of processes, including a new connection with the oxidative stress response mediated by HxlR.

Metabolism (CodY regulon)

In **Appendix Figure S7**, we present a functional analysis of the CodY regulon. Importantly, our predictions are in good agreement with two recent publications reporting the global identification of CodY targets (Belitsky & Sonenshein, 2013; Brinsmade *et al*, 2014). We observed that the following processes are repressed by CodY: i) biosynthesis of branched chain amino acids (BCAA); ii) exploitation of alternative sources of nitrogen, including uptake and utilization of peptides and amino acids, regulation of the urea cycle, and protein degradation (2 new targets, *ispA* and *vpr*, encoding two serine proteases, contribute to this process). Recently, *vpr* was described as a CodY target (Barbieri *et al*, 2015); iii) carbon metabolism, with target genes encoding enzymes of the tricarboxylic acid cycle (*citB* and *acsA*); iv) production of antibacterial compounds, including *bacG* (a previously known target involved in the synthesis of bacilysin, whose regulation is also dependent on ScoC) and the *pks* operon (a novel target involved in the synthesis of polyketides); v) initiation of sporulation (*rapA-phrA*, *rapE-phrE* and *kinB*), by controlling the phosphorylation of Spo0F, an intermediate in the pathway leading to activation of Spo0A; vi) membrane fluidity (*yuaF-floT-yuaI* operon); vii) 13 novel targets of unknown function (*yknV*, *amhX*, *yjbA*, *sndC*, *yfmB*, *ykwB*, *yjcL*, *yjcK*, *yocS*, *yvdA*, *ywqJ*, *yqfZ* and *yhjB* in an operon with a previously known target, *yhjC*). Thus, the addition of several genes of unknown function to the regulons of well-known regulators

like ComK and CodY shows that even the best characterized cell processes contain previously unrecognized components and new connections to other cell processes.

<u>*Sporulation*</u>

In **Appendix Figure S8A**, we analyzed the expression of *yetF*, which encodes a putative membrane protein of unknown function. In our model, *yetF* is predicted to be a target of σ^F. Just upstream of *yetF, lplD* was previously recognized as a σ^E target (Eichenberger *et al*, 2004). Transcriptional profiling experiments with *sigF* and *sigE* mutant strains confirm that *lplD* is differentially expressed in a *sigF* mutant, while its level remains almost unchanged in a *sigE* deletion mutant. We conclude that *yetF* is primarily under σ^F control. Inspection of the sequence directly upstream of *yetF* reveals a near perfect match to the consensus sequence for σ^F promoters. In wild type cells, YetF-GFP localization is similar to that of other forespore membrane proteins, i.e. a ring of green fluorescence overlapping with the location of the forespore membranes. As expected, both *sigF* and *sigE* mutants exhibited the classic disporic phenotype characteristic of *sigE* inactivation [with polar septa formed at both ends of the sporulating cell (Pogliano *et al*, 1999), but a green fluorescent signal was only detected in a *sigE* mutant, demonstrating that *yetF* expression does not strictly require the σ^E-dependent promoter. Control experiments showed that the pattern of YetF-GFP localization is unaffected by inactivation of σ^G or σ^K.

In **Appendix Figure S8B**, we validate the prediction that the *ykoS-ykoT* operon is a novel target of σ^G. Both genes are predicted to encode membrane proteins, but, in addition, YkoT is homologous to a family of glycosyltransferases. Downstream of *ykoT*, we find a previously characterized sporulation operon, *ykoV-ligD*, involved in DNA repair via a non-homologous end joining mechanism (Wang *et al*, 2006). This second operon is known to be the target of a coherent FFL formed by σ^G and SpoVT. Transcriptional profiling experiments confirm that, as predicted, both operons are regulated by σ^G; however, they also show that SpoVT is an activator of *ykoV-ligD* and a repressor of *ykoST* (therefore, *ykoST* operon is the target of an incoherent FFL formed by σ^G and SpoVT). Thus, although both regulators were correctly identified for *ykoV* (a gene included in the GS), the prediction for *ykoST* was incomplete (because it did not include SpoVT). As observed for many other targets of FFLs, the primary regulator (σ^G) was accurately predicted, but the contribution of the secondary regulator (SpoVT) was missed. Importantly, we identified a putative binding site for σ^G with a good match to the consensus upstream of *ykoS*. In addition, as expected, a GFP fusion to YkoT showed the typical expression pattern of a

forespore membrane protein. Furthermore, no expression was detected in a *sigG* mutant, while subcellular localization was not affected in either the *spoVT* or *sigK* mutants.

In **Appendix Figure S8C**, we characterize the expression and sub-cellular localization during sporulation of YkzQ-GFP, a putative novel target of σ^K. The gene immediately upstream of *ykzQ* (*ykvP*) is a previously known target of σ^K and GerE (Kodama *et al*, 2000). YkzQ is a small protein of 75 a.a., which was missed in the original annotation of the *B. subtilis* genome sequence (Kunst *et al*, 1997). It contains a LysM domain known to promote binding to peptidoglycan (Buist *et al*, 2008) and frequently found in spore coat proteins. We confirmed the prediction that expression of *ykzQ* was dependent on σ^K (presumably from the promoter located upstream of *ykvP*). YkzQ-GFP localizes to the spore coat, more specifically the outer coat, since localization was impaired in a *cotE* mutant.

REFERENCES

Bailey TL, Boden M, Buske FA, Frith M, Grant CE, Clementi L, Ren J, Li WW, Noble WS (2009) MEME SUITE: tools for motif discovery and searching. *Nucleic Acids Res.* **37:** W202–W208

Baldi P, Long AD (2001) A Bayesian framework for the analysis of microarray expression data: regularized t -test and statistical inferences of gene changes. *Bioinformatics* **17:** 509–519

Barbieri G, Voigt B, Albrecht D, Hecker M, Albertini AM, Sonenshein AL, Ferrari E, Belitsky BR (2015) CodY regulates expression of the *Bacillus subtilis* extracellular proteases Vpr and Mpr. *J. Bacteriol.* **197**: 1423-1432

Belitsky BR, Sonenshein AL (2013) Genome-wide identification of *Bacillus subtilis* CodY-binding sites at single-nucleotide resolution. *Proc. Natl. Acad. Sci. U. S. A.* **110:** 7026–7031

Brinsmade SR, Alexander EL, Livny J, Stettner AI, Segrè D, Rhee KY, Sonenshein AL (2014) Hierarchical expression of genes controlled by the *Bacillus subtilis* global regulatory protein CodY. *Proc. Natl. Acad. Sci. U. S. A.* **111:** 8227–8232

Buist G, Steen A, Kok J, Kuipers OP (2008) LysM, a widely distributed protein motif for binding to (peptido)glycans. *Mol. Microbiol.* **68:** 838–847

Crutz AM, Steinmetz M (1992) Transcription of the *Bacillus subtilis sacX* and *sacY* genes, encoding regulators of sucrose metabolism, is both inducible by sucrose and controlled by the DegS-DegU signalling system. *J. Bacteriol.* **174:** 6087–6095

Dehal PS, Joachimiak MP, Price MN, Bates JT, Baumohl JK, Chivian D, Friedland GD, Huang KH, Keller K, Novichkov PS, Dubchak IL, Alm EJ, Arkin AP (2010) MicrobesOnline: an integrated portal for comparative and functional genomics. *Nucleic Acids Res.* **38:** D396–D400

Eichenberger P, Fujita M, Jensen ST, Conlon EM, Rudner DZ, Wang ST, Ferguson C, Haga K, Sato T, Liu JS, Losick R (2004) The program of gene transcription for a single differentiating cell type during sporulation in *Bacillus subtilis*. *PLoS Biol.* **2:** e328

Faith JJ, Hayete B, Thaden JT, Mogno I, Wierzbowski J, Cottarel G, Kasif S, Collins JJ, Gardner TS (2007) Large-Scale Mapping and Validation of *Escherichia coli* Transcriptional Regulation from a Compendium of Expression Profiles. *PLoS Biol.* **5:** e8

Fawcett P, Eichenberger P, Losick R, Youngman P (2000) The transcriptional profile of early to middle sporulation in *Bacillus subtilis*. *Proc. Natl. Acad. Sci. U. S. A.* **97:** 8063–8068

Helmann JD (1995) Compilation and analysis of *Bacillus subtilis* sigma A-dependent promoter sequences: evidence for extended contact between RNA polymerase and upstream promoter DNA. *Nucleic Acids Res.* **23:** 2351–2360

Huang Y, Niu B, Gao Y, Fu L, Li W (2010) CD-HIT Suite: a web server for clustering and comparing biological sequences. *Bioinformatics* **26:** 680–682

Huynh-Thu VA, Irrthum A, Wehenkel L, Geurts P (2010) Inferring regulatory networks from expression data using tree-based methods. *PLoS One* **5:** e12776

Irnov I, Sharma CM, Vogel J, Winkler WC (2010) Identification of regulatory RNAs in *Bacillus subtilis*. *Nucleic Acids Res.* **38:** 6637–6651

Kim H, Hahn M, Grabowski P, McPherson DC, Otte MM, Wang R, Ferguson CC, Eichenberger P, Driks A (2006) The *Bacillus subtilis* spore coat protein interaction network. *Mol. Microbiol.* **59:** 487–502

Kodama T, Takamatsu H, Asai K, Ogasawara N, Sadaie Y, Watabe K (2000) Synthesis and characterization of the spore proteins of *Bacillus subtilis* YdhD, YkuD, and YkvP, which carry a motif conserved among cell wall binding proteins. *J. Biochem.* **128:** 655–663

Kramer N, Hahn J, Dubnau D (2007) Multiple interactions among the competence proteins of *Bacillus subtilis*. *Mol. Microbiol.* **65:** 454–464

Kunst F, Ogasawara N, Moszer I, Albertini AM, Alloni G, Azevedo V, Bertero MG, Bessières P, Bolotin A, Borchert S, Borriss R, Boursier L, Brans A, Braun M, Brignell SC, Bron S, Brouillet S, Bruschi C V, Caldwell B, Capuano V, et al (1997) The complete genome sequence of the Gram-positive bacterium *Bacillus subtilis*. *Nature* **390:** 249–256

Marciniak BC, Pabijaniak M, de Jong A, Dűhring R, Seidel G, Hillen W, Kuipers OP (2012) High- and low-affinity *cre* boxes for CcpA binding in *Bacillus subtilis* revealed by genome-wide analysis. *BMC Genomics* **13:** 401

Margolin AA, Nemenman I, Basso K, Wiggins C, Stolovitzky G, Dalla Favera R, Califano A (2006) ARACNE: an algorithm for the reconstruction of gene regulatory networks in a mammalian cellular context. *BMC Bioinformatics* **7 (Suppl 1):** S7

McKenney PT, Driks A, Eskandarian HA, Grabowski P, Guberman J, Wang KH, Gitai Z, Eichenberger P (2010) A distance-weighted interaction map reveals a previously uncharacterized layer of the *Bacillus subtilis* spore coat. *Curr. Biol.* **20:** 934–938

Michna RH, Commichau FM, Tödter D, Zschiedrich CP, Stülke J (2014) SubtiWiki-a database for the model organism *Bacillus subtilis* that links pathway, interaction and expression information. *Nucleic Acids Res.* **42:** D692–D698

Molle V, Fujita M, Jensen ST, Eichenberger P, González-Pastor JE, Liu JS, Losick R (2003) The Spo0A regulon of *Bacillus subtilis*. *Mol. Microbiol.* **50:** 1683–1701

Morris JH, Apeltsin L, Newman AM, Baumbach J, Wittkop T, Su G, Bader GD, Ferrin TE (2011) *clusterMaker*: a multi-algorithm clustering plugin for Cytoscape. *BMC Bioinformatics* **12:** 436

Van Ooij C, Eichenberger P, Losick R (2004) Dynamic patterns of subcellular protein localization during spore coat morphogenesis in *Bacillus subtilis*. *J. Bacteriol.* **186:** 4441–4448

Pogliano J, Osborne N, Sharp MD, Abanes-De Mello A, Perez A, Sun YL, Pogliano K (1999) A vital stain for studying membrane dynamics in bacteria: a novel mechanism controlling septation during *Bacillus subtilis* sporulation. *Mol. Microbiol.* **31:** 1149–1159

Pragai Z, Hardwood CR (2000) Screening for mutants affected in their response to phosphate. In *Functional Analysis of Bacterial Genes: a Practical Manual*, Schumann W , Ehrlich SD & Ogasawara N (ed) pp 245–249. Chichester: Wiley

Prestel E, Noirot P, Auger S (2015) Genome-wide identification of *Bacillus subtilis* Zur-binding sites associated with a Zur box expands its known regulatory network. *BMC Microbiol.* **15:** 13

Rasmussen S, Nielsen HB, Jarmer H (2009) The transcriptionally active regions in the genome of *Bacillus subtilis*. *Mol. Microbiol.* **73:** 1043–1057

Rochat T, Nicolas P, Delumeau O, Rabatinová A, Korelusová J, Leduc A, Bessières P, Dervyn E, Krásny L, Noirot P (2012) Genome-wide identification of genes directly regulated by the pleiotropic transcription factor Spx in *Bacillus subtilis*. *Nucleic Acids Res.* **40:** 9571–9583

Sales G, Romualdi C (2011) parmigene--a parallel R package for mutual information estimation and gene network reconstruction. *Bioinformatics* **27:** 1876–1877

Smoot ME, Ono K, Ruscheinski J, Wang P-L, Ideker T (2011) Cytoscape 2.8: new features for data integration and network visualization. *Bioinformatics* **27:** 431–432

Smyth GK (2004) Linear models and empirical bayes methods for assessing differential expression in microarray experiments. *Stat. Appl. Genet. Mol. Biol.* **3:** Article3

Sterlini JM, Mandelstam J (1969) Commitment to sporulation in *Bacillus subtilis* and its relationship to development of actinomycin resistance. *Biochem. J.* **113:** 29–37

Wang KH, Isidro AL, Domingues L, Eskandarian HA, McKenney PT, Drew K, Grabowski P, Chua M-H, Barry SN, Guan M, Bonneau R, Henriques AO, Eichenberger P (2009) The coat morphogenetic protein SpoVID is necessary for spore encasement in *Bacillus subtilis*. *Mol. Microbiol.* **74:** 634–649

Wang ST, Setlow B, Conlon EM, Lyon JL, Imamura D, Sato T, Setlow P, Losick R, Eichenberger P (2006) The forespore line of gene expression in *Bacillus subtilis*. *J. Mol. Biol.* **358:** 16–37

Warsow G, Greber B, Falk SSI, Harder C, Siatkowski M, Schordan S, Som A, Endlich N, Schöler H, Repsilber D, Endlich K, Fuellen G (2010) *ExprEssence* - Revealing the essence of differential experimental data in the context of an interaction/regulation net-work. *BMC Syst. Biol.* **4:** 164

Yasueda H, Kawahara Y, Sugimoto S (1999) *Bacillus subtilis yckG* and *yckF* Encode Two Key Enzymes of the Ribulose Monophosphate Pathway Used by Methylotrophs, and *yckH* Is Required for Their Expression. *J. Bacteriol.* **181:** 7154–7160

Zellner A (1983) Applications of Bayesian analysis in econometrics. *Stat.* **32:** 23–24

Appendix Table S1. Confidence of novel predictions depends on the number of priors.

Priors$	TFs	Recovered priors*	Recovery rate	Median confidence recovered priors	Novel interactions	Averaged number of novel targets	Median confidence novel targets
0	63	NA	NA	NA	299	4.7	39040.56
1 to 5	81	214	0.98	593.67	650	8	40837.2
6 to 10	25	171	0.92	1486.54	275	11	39237.01
>10	46	1873	0.73	2300.38	1034	22.5	31944.77

This table reports the effect that the number of priors per TF has on the confidence scores of predicted interactions (for recovered priors and novel interactions) and the average number of novel targets per category.

$ number of known targets in the GS network (excluding auto-regulation)
* present in the GS network and included in our model

Appendix Table S2. Regulatory hypotheses by functional categories

Gene category[#*]	Total genes	Genes in the GS[&]	%	Genes in the inferred TRN[&]	%
Cell envelope and cell division	191	103	53.9	147	77
Transporters	437	237	54.2	354	81
Homeostasis	95	59	62.1	79	83.2
Electron transport and ATP synthesis	98	54	55.1	74	75.5
Carbon metabolism	285	191	67	232	81.4
Amino acid/ nitrogen metabolism	254	157	61.8	203	79.9
Lipid metabolism	89	52	58.4	69	77.5
Nucleotide metabolism	106	66	62.3	80	75.5
Additional metabolic pathways	336	176	52.4	270	80.4
Genetics	176	90	51.1	117	66.5
RNA synthesis and degradation	66	26	39.4	32	48.5
Protein synthesis, modification and degradation	486	142	29.2	251	51.6
Regulation of gene expression	434	216	49.8	274	63.1
Exponential and early post-exponential lifestyles	176	137	77.8	150	85.2
Sporulation and germination	565	418	74	528	93.5
Coping with stress	584	407	69.7	476	81.5
Prophages	291	37	12.7	95	32.6
Essential genes	278	114	41	180	64.7
Membrane proteins	1095	559	51.1	836	76.3
Phosphoproteins	283	145	51.2	200	70.7
Poorly characterized/putative enzymes	204	67	32.8	138	67.6
Unknown functions	872	124	14.2	521	59.7

This table reports the number of genes paired with a regulator in the prior network (GS) and in the inferred transcriptional regulatory network (TRN) by functional category. Functional categories are taken from SubtiWiki.

[#] gene categories (from SubtiWiki)
[*] only categories with 50 or more genes are shown
[&] number of genes with a least one transcription factor

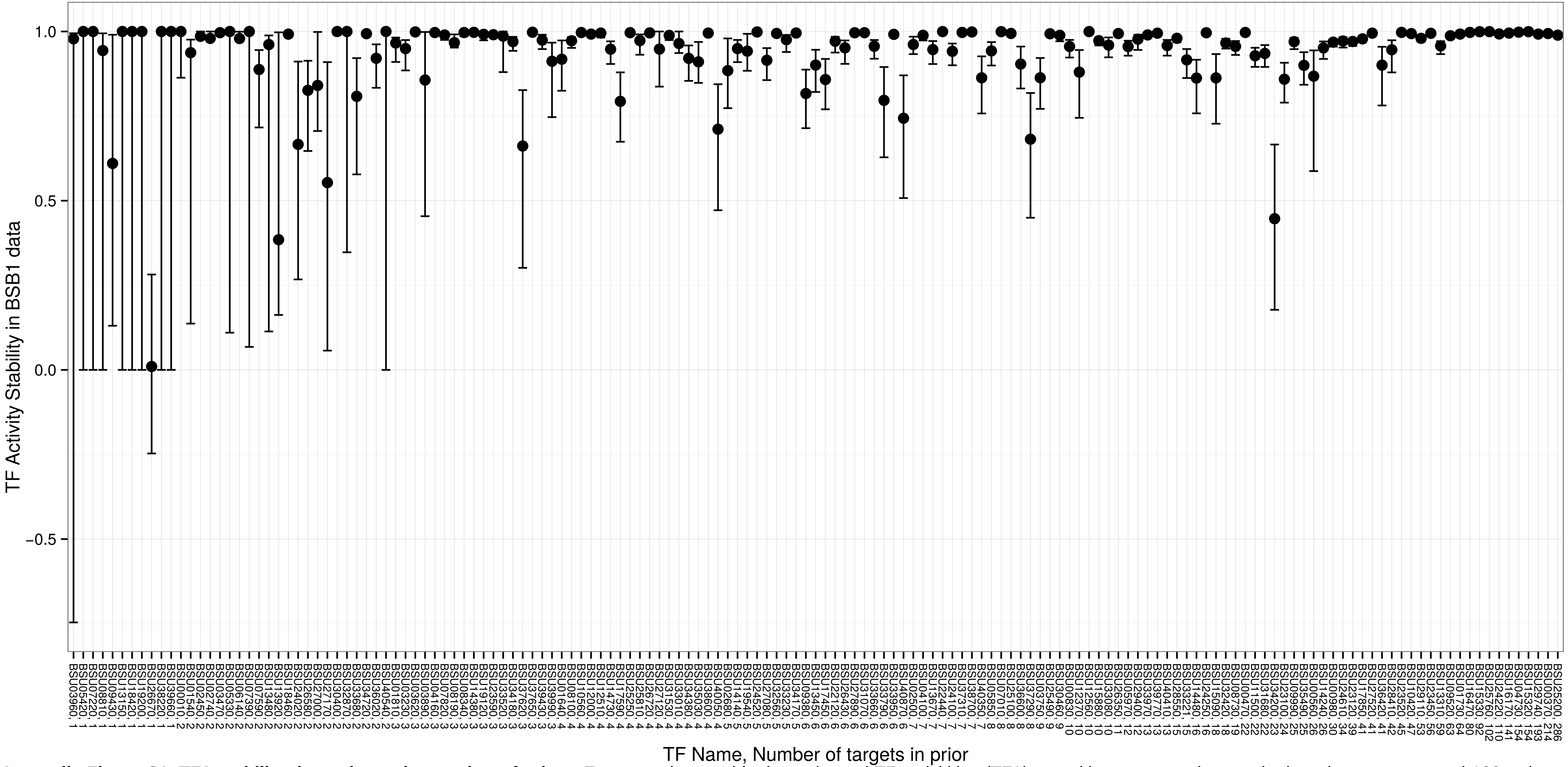

Appendix Figure S1. TFA stability depends on the number of priors. To assess how stable the estimated TF activitities (TFA) are with respect to changes in the prior, we generated 128 reduced priors, by randomly removing 20% of the prior interactions. The resulting activity profiles for each TF are compared by Pearson correlation. Here we show the distribution of all pairwise activity correlations for each TF in the prior. Error bars indicate 25th and 75th percentile, points indicate the median.

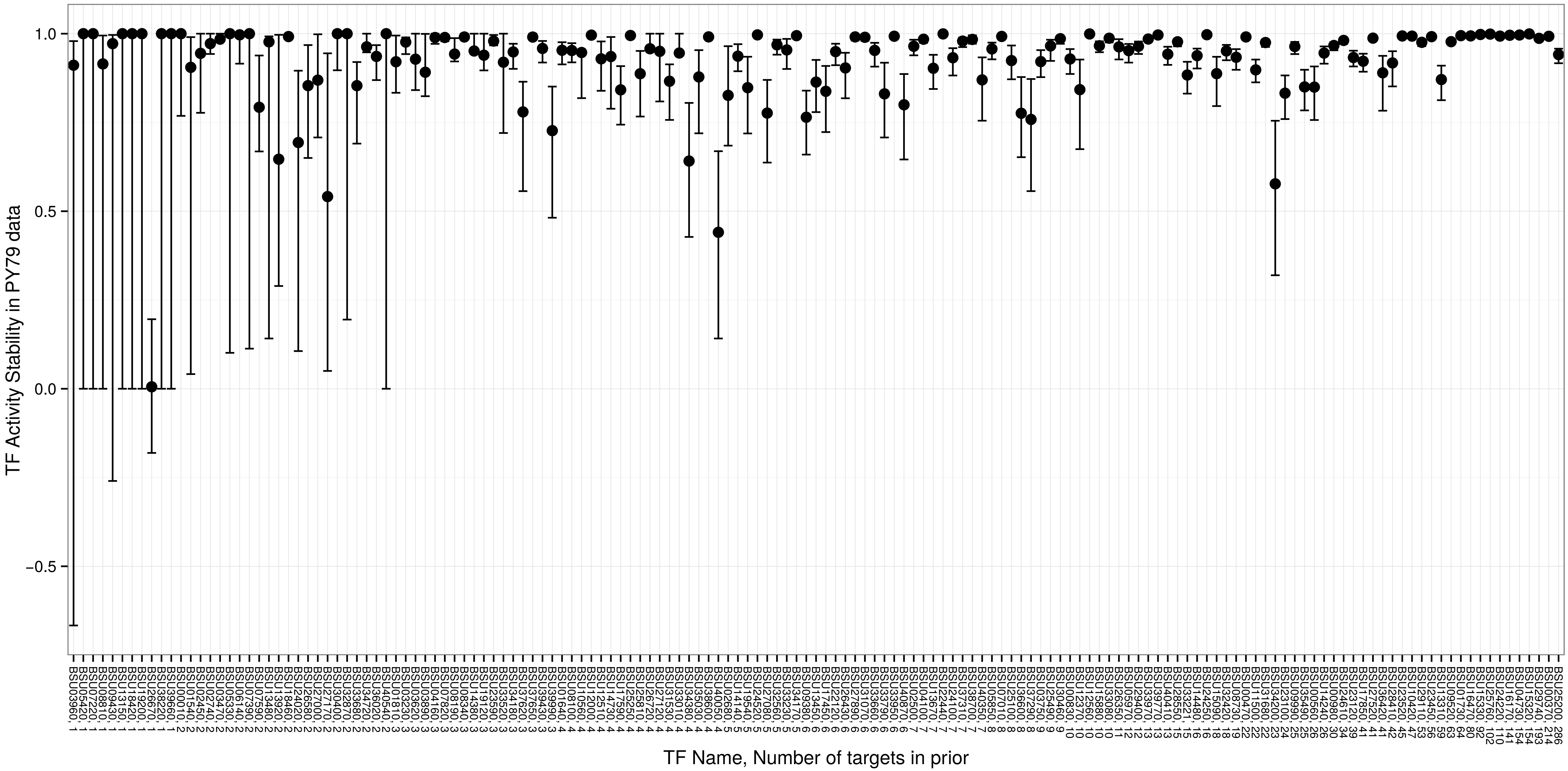

Appendix Figure S2. TFA stability depends on the number of priors. To assess how stable the estimated TF activitities (TFA) are with respect to changes in the prior, we generated 128 reduced priors, by randomly removing 20% of the prior interactions. The resulting activity profiles for each TF are compared by Pearson correlation. Here we show the distribution of all pairwise activity correlations for each TF in the prior. Error bars indicate 25th and 75th percentile, points indicate the median.

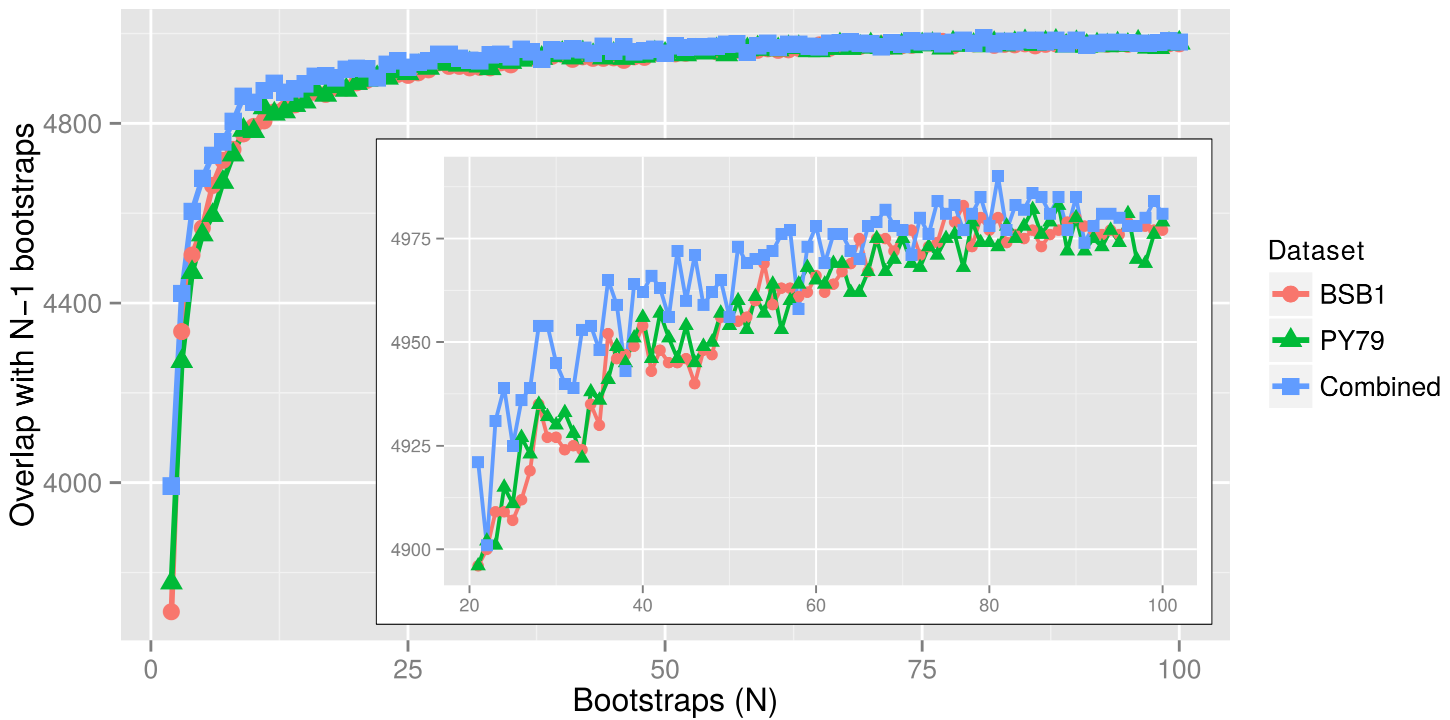

Appendix Figure S3. Top 5000 interactions of BBSR-TFA predictions differ less than 5% after 20 or more bootstraps. We compared the top 5000 interactions for inferred networks using 2 up to 100 bootstraps in the BSB1 dataset, PY79 dataset or both (combined) to the top 5000 interactions using one less bootstrap. The number of overlaps for each comparison is shown in the Y axis.

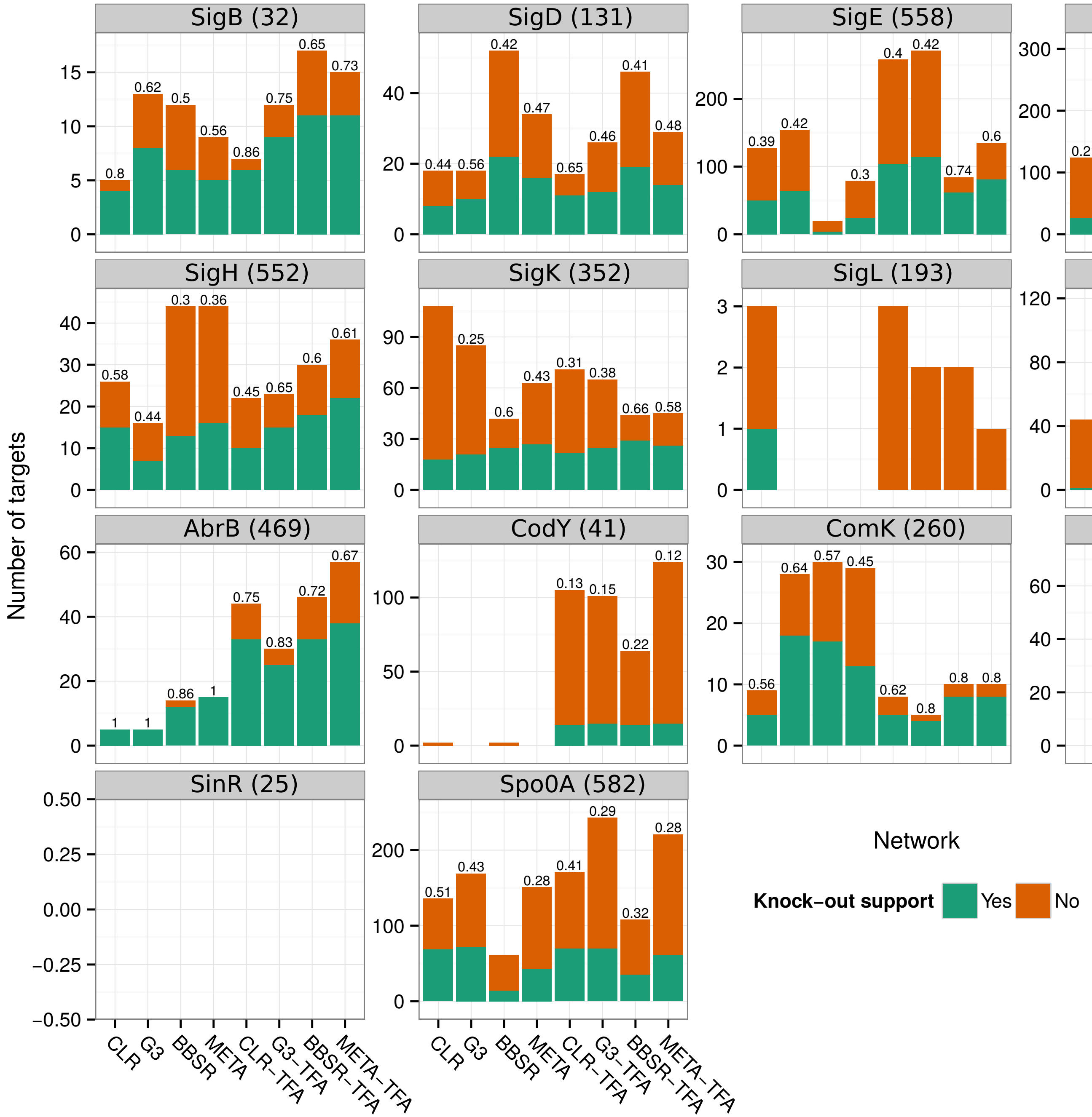

Appendix Figure S4. Support from KO data to novel interactions predicted by BBSR, Genie3 and CLR. For each regulon with KO data we assessed the proportion of novel targets (not included in the GS) supported by the KO data for BBSR, Genie3 (G3), CLR and a consensus method (META) that rank combines the prediction of the three methods. Methods were used without and with TFA (TFA tag). The number in parentheses next to regulon's name indicates the number of differentially transcribed genes in the corresponding KO data that are not known targets of the corresponding TF in the GS network. The number on top each bar indicates the proportion of evaluated interactions (novel interactions) supported by the KO data. This number is omitted if there was no significant (p-value ≥ 0.01) enrichment for differentially transcribed genes in the predicted targets

TF	Identified motif	E-value	Motif present in recovered priors			Motif present in novel targets		
			Yes	No	%yes	Yes	No	% yes
SigB	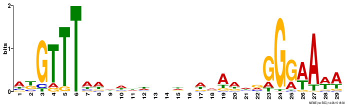	3.60E-078	88	8	92	12	2	86
SigD	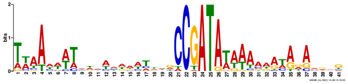	1.20E-035	24	3	89	10	10	50
SigE	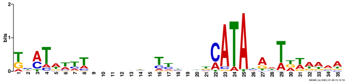	3.80E-050	81	4	95	60	1	98
SigG	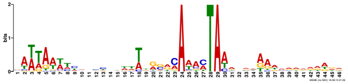	8.20E-020	39	19	67	39	30	57
SigH	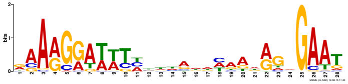	5.70E-003	12	6	67	17	14	55
SigK	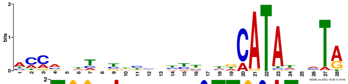	6.70E-026	46	8	85	38	12	76
SigL	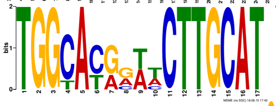	6.10E-015	6	0	100	-	-	-
SigW	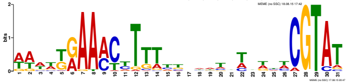	2.10E-049	27	1	96	1	0	100
CcpA-repression	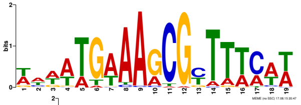	1.30E-062	33	18	65	11	9	55
CodY-repression	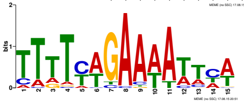	8.40E-004	21	9	70	12	5	71

Appendix Figure S5. Presence of putative binding sites in predicted target operons. Putative binding sites found in the sequence upstream of operons predicted as targets for the corresponding TFs. Identified sequences agree with previously recognized motifs. The number of targets displaying the motif is shown for each regulon. An operon was considered to be “known target” if at least one of its members was annotated as target in the GS.

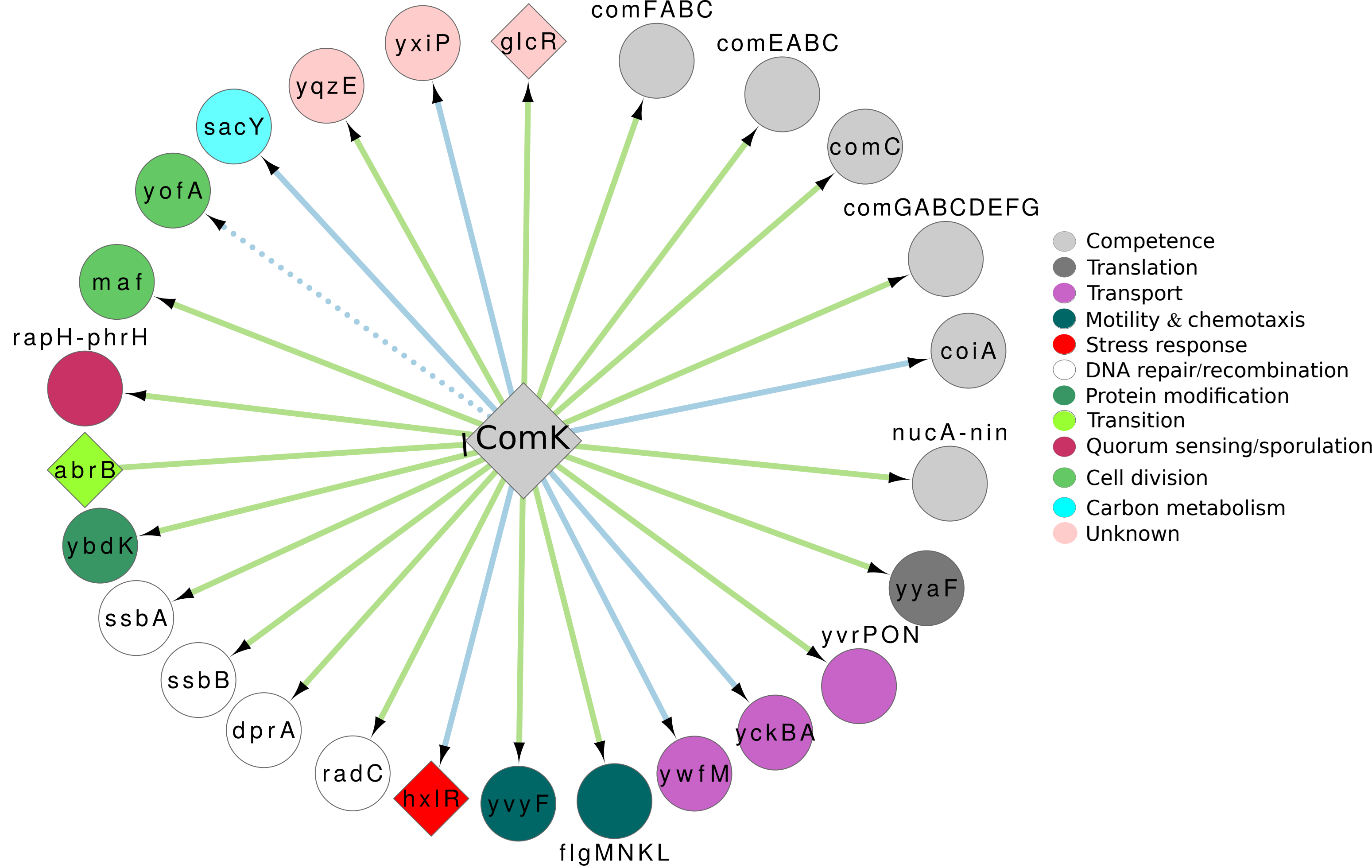

Appendix Figure S6. Functional analysis of the ComK regulon. Predicted targets of ComK. Green and blue edges represent interactions present in the GS and novel targets, respectively. Blue solid lines indicate interactions supported by the *comK* KO data. Dotted lines indicate interactions not supported by the KO data. Operons with genes assigned to more than one functional category were subdivided based on the functional annotation. Functional categories are color-coded. Diamonds indicate transcription factors.

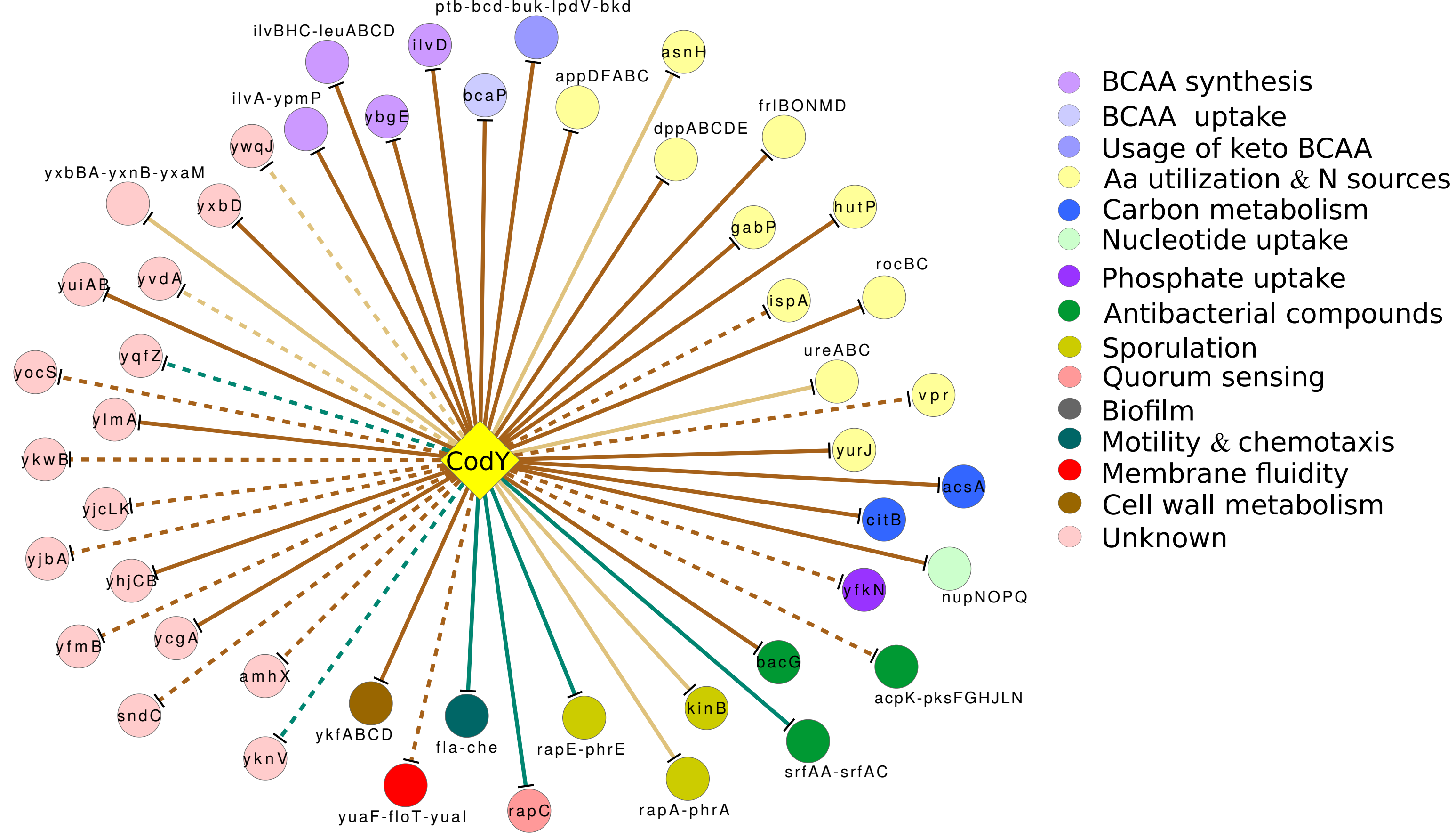

Appendix Figure S7. Functional analysis of the CodY regulon. Predicted targets of CodY. Novel interactions are indicated by dashed lines. Interactions recovered from the GS are indicated by solid lines. Dark brown lines indicate interactions supported by transcriptional profiles, motif searches and ChIP-seq data (Belitsky & Sonenshein, 2013). Light brown lines indicate interactions supported by two out of the three supporting data types. Seagreen lines indicate interactions supported by one out of the three supporting data types. Operons with genes assigned to more than one functional category were subdivided based on the functional annotation. Functional categories are color-coded. Diamonds indicate transcription factors.

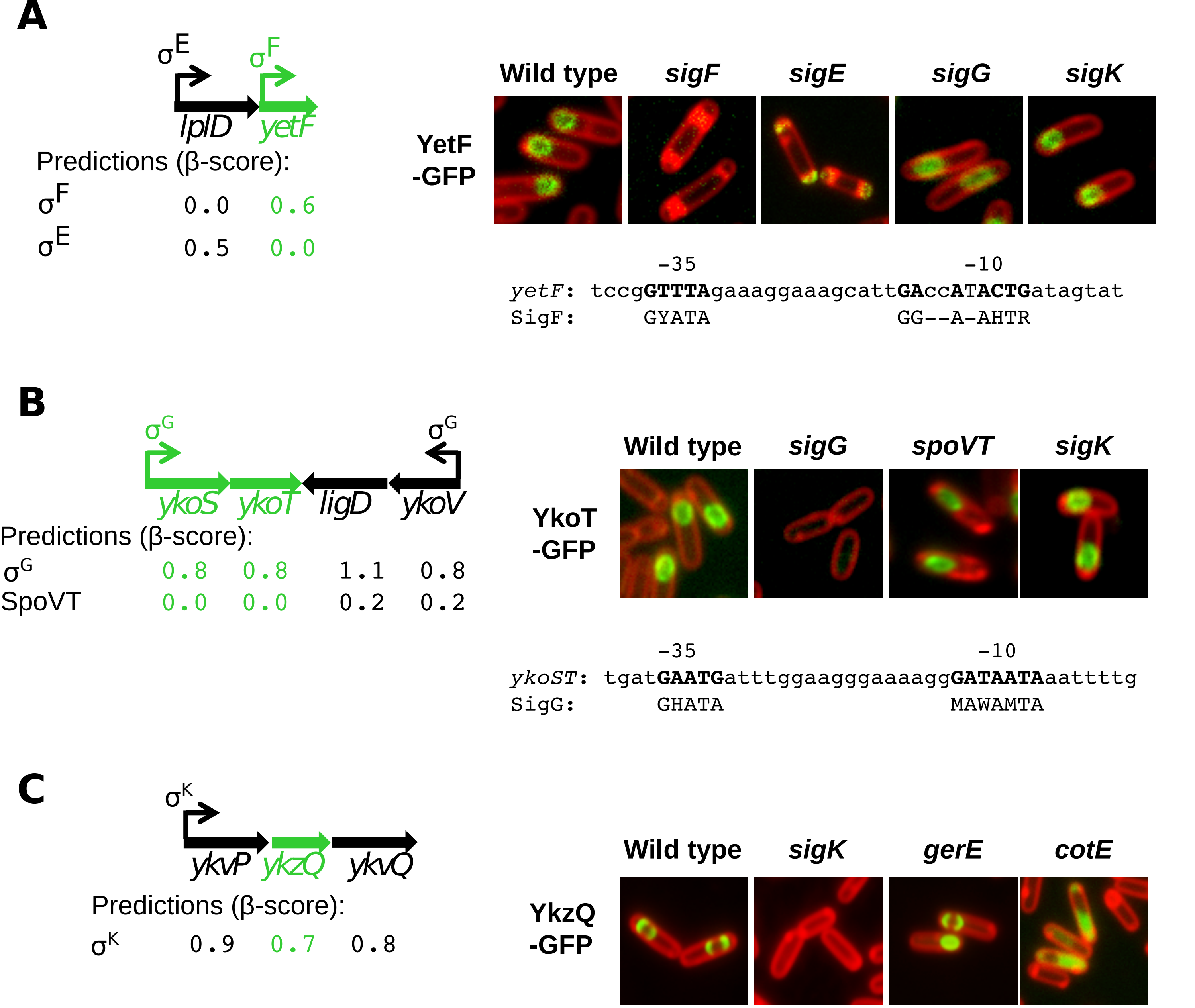

Appendix Figure S8. Characterization of novel sporulation genes by fluorescence microscopy.
A) Characterization of *yetF* transcription and subcellular localization of YetF-GFP. Images were collected at hour 3 of sporulation after suspension in Sterlini-Mandelstam medium at 37°C. The putative σ^F promoter is indicated (H is A or C or T; R is A or G; Y is C or T).
B) Characterization of *ykoST* transcription and subcellular localization of YkoT-GFP. Images were collected at hour 5 of sporulation after suspension in Sterlini-Mandelstam medium at 37°C. The putative σ^G promoter is indicated (H is A or C or T; M is A or C; W is A or T).
C) Characterization of *ykzQ* transcription and subcellular localization of YkzQ-GFP. Images were collected at hour 6 of sporulation after suspension in Sterlini-Mandelstam medium at 37°C.
