# Supplementary material for: An experimentally supported model of the Bacillus subtilis global transcriptional regulatory network
Source: Mol Syst Biol. 2015 Nov 17;11(11):839. doi: 10.15252/msb.20156236 (PMC4670728; doi:10.15252/msb.20156236)
Supplement: Supplementary file 6 — Dataset EV3 [file MSB-11-839-s006.zip › Dataset_EV3/EV3.pdf]

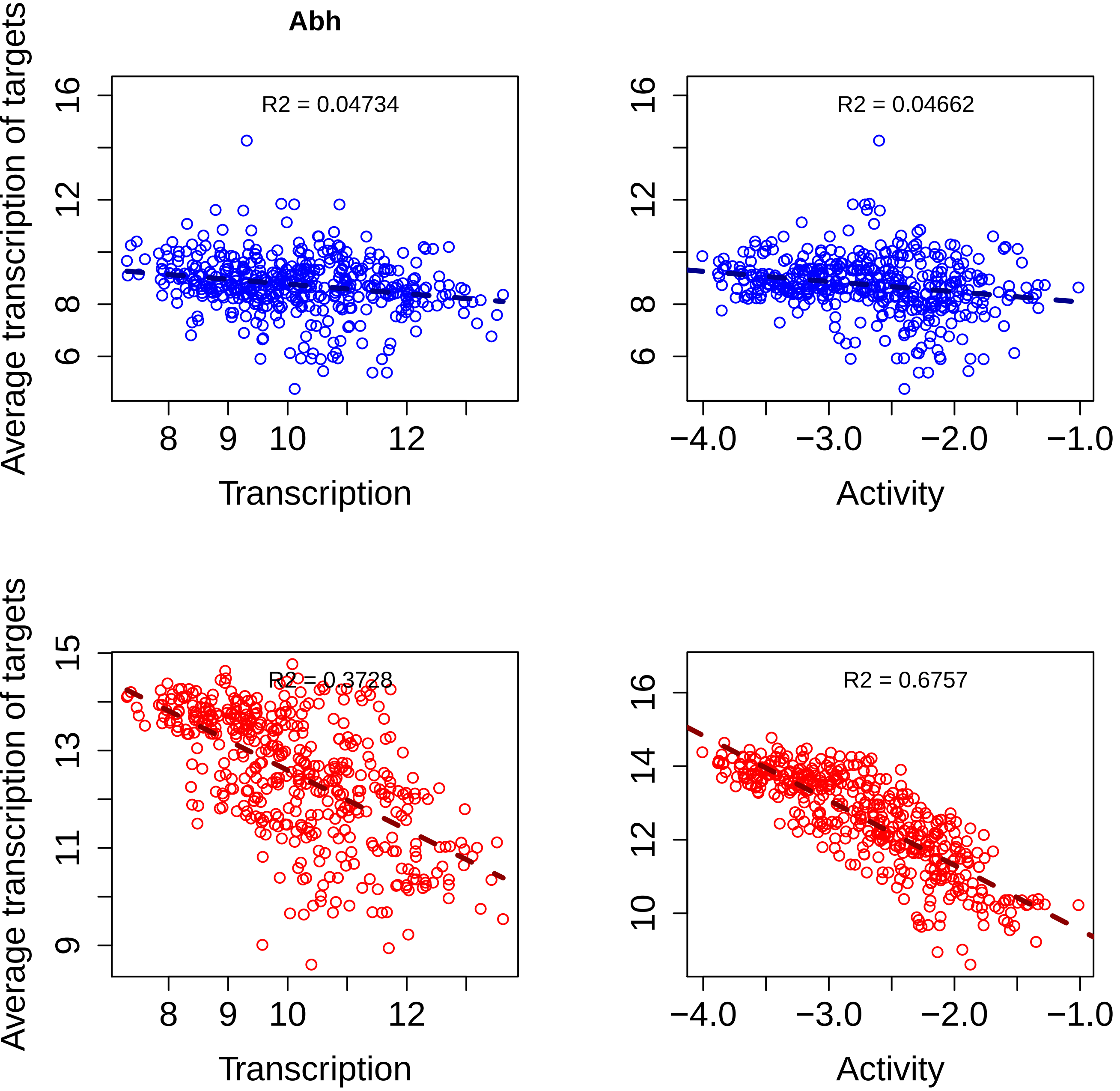

Abh
Average transcription of targets
R2 = 0.04734
Transcription
R2 = 0.04662
Activity
R2 = 0.3728
R2 = 0.6757
6
8
12
16
9
11
13
15
10
14
9
10
12
−4.0
−3.0
−2.0
−1.0

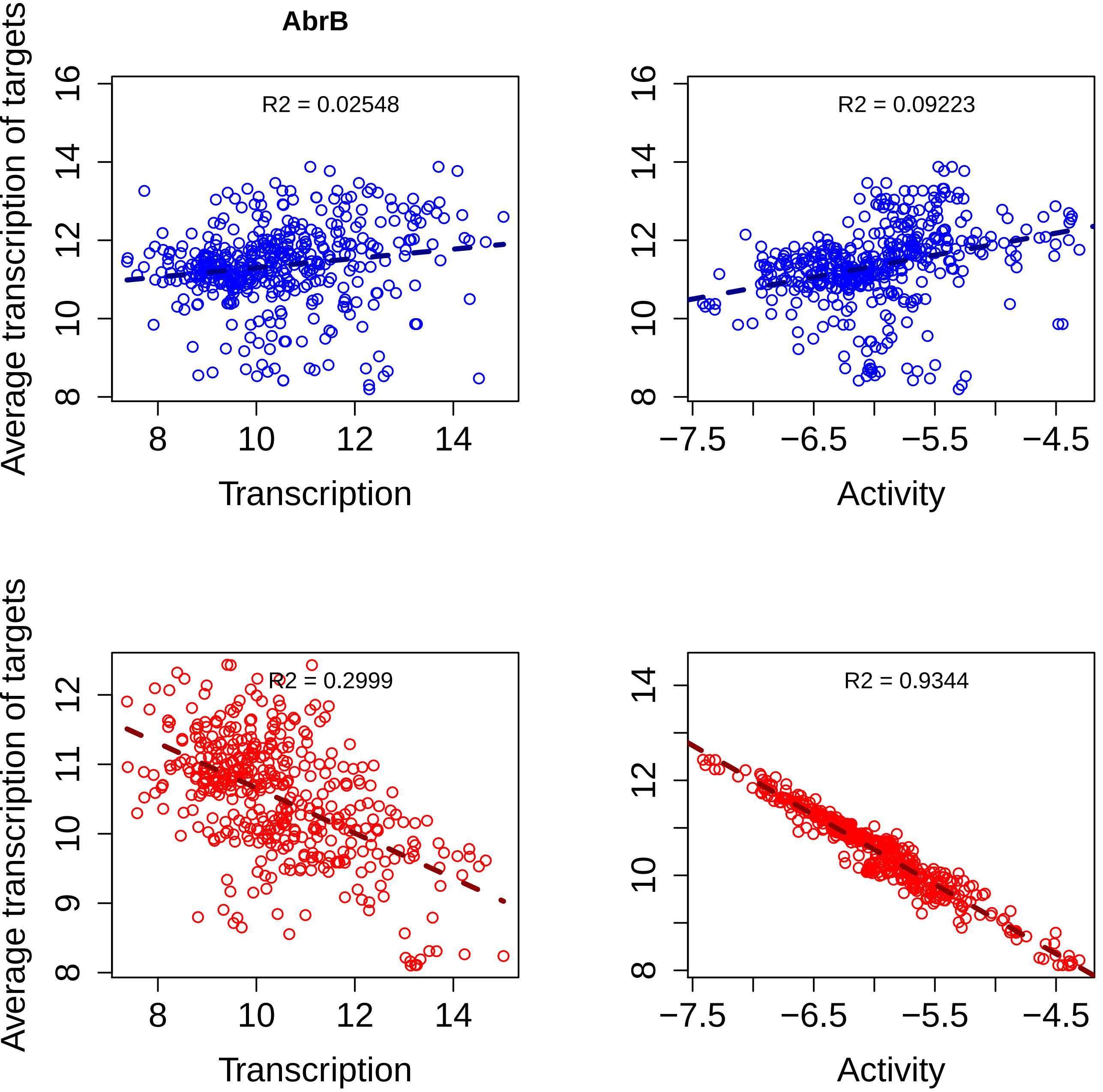

AbrB
Average transcription of targets
R2 = 0.02548
R2 = 0.09223
R2 = 0.2999
R2 = 0.9344
Transcription
Activity
8
10
12
14
16
−7.5
−6.5
−5.5
−4.5
9
11

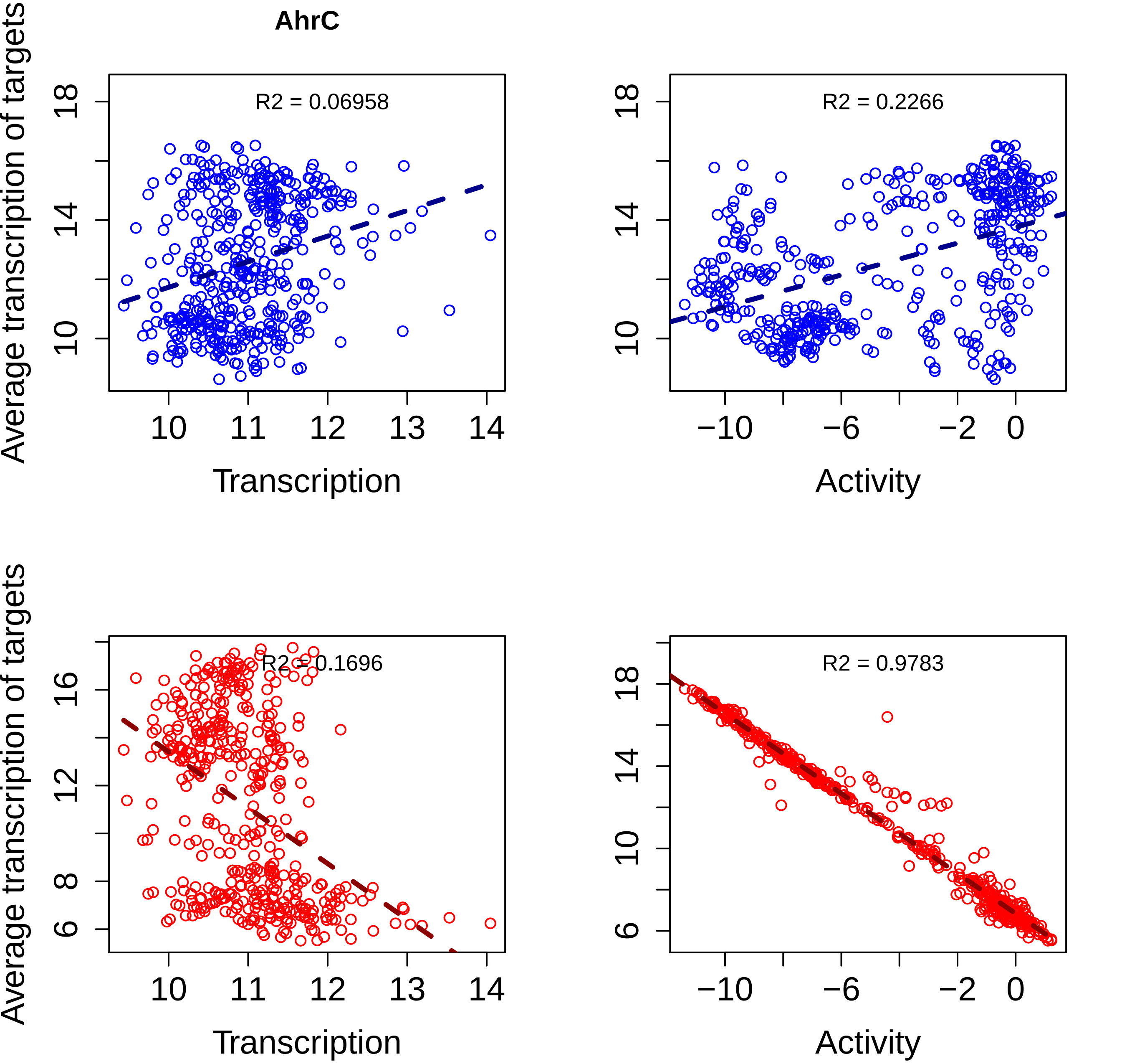

AhrC
Average transcription of targets
R2 = 0.06958
R2 = 0.2266
R2 = 0.1696
R2 = 0.9783
Transcription
Activity
10
11
12
13
14
−10
−6
−2
0
18
14
10
16
12
8
6

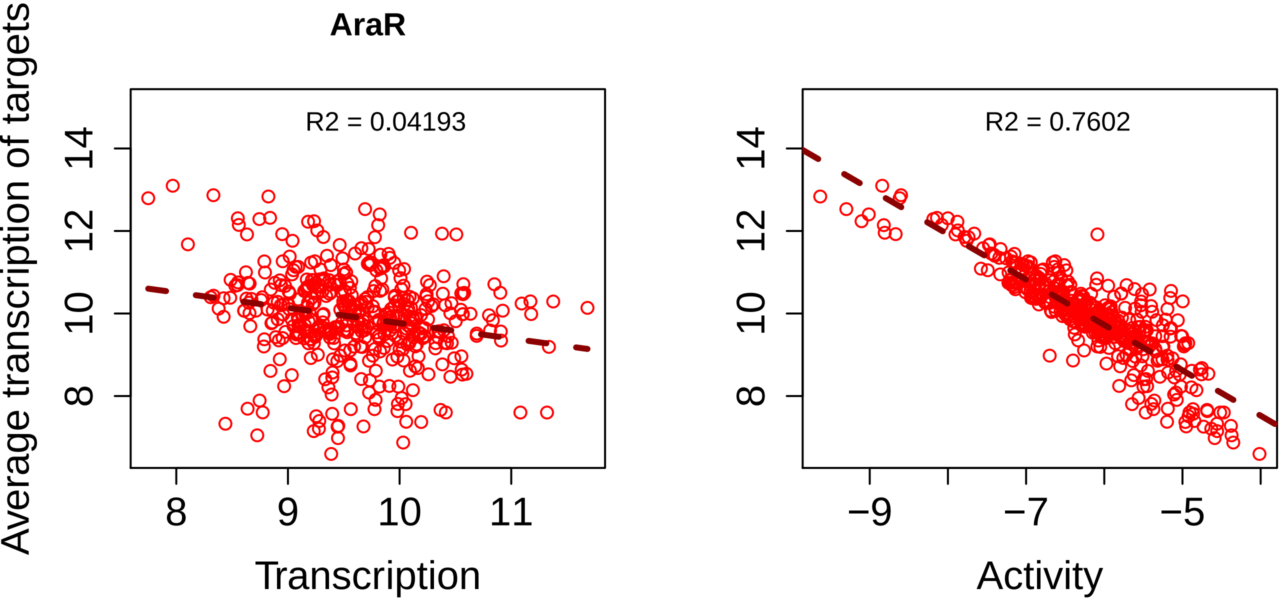
AraR
Average transcription of targets
R2 = 0.04193
R2 = 0.7602
8
10
12
14
8
9
10
11
−9
−7
−5
Transcription
Activity

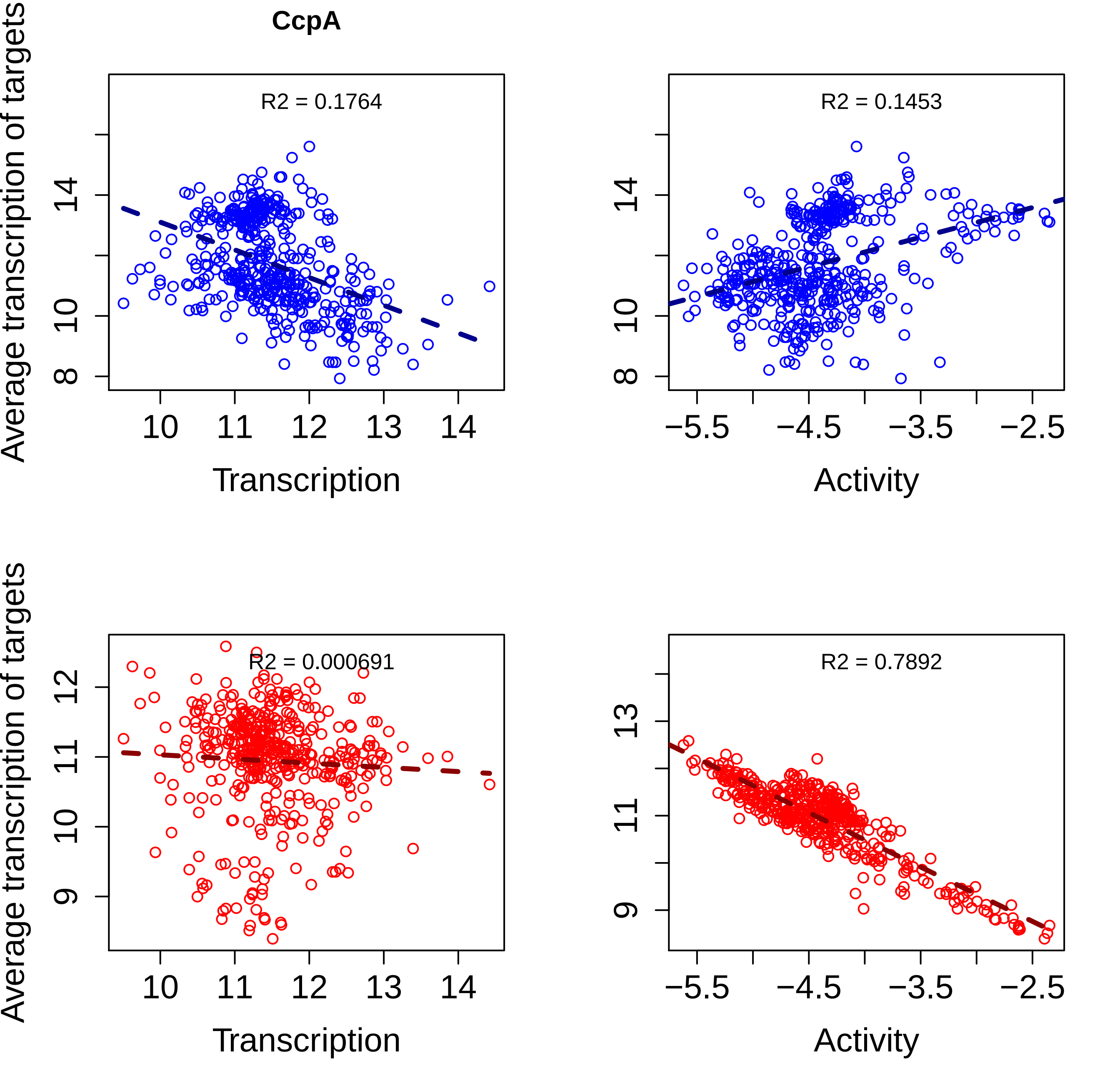

CcpA
R2 = 0.1764
R2 = 0.1453
R2 = 0.000691
R2 = 0.7892
Average transcription of targets
Average transcription of targets
Transcription
Activity
Transcription
Activity
8
10
14
10
11
12
13
14
−5.5
−4.5
−3.5
−2.5
9
10
11
12
9
11
13

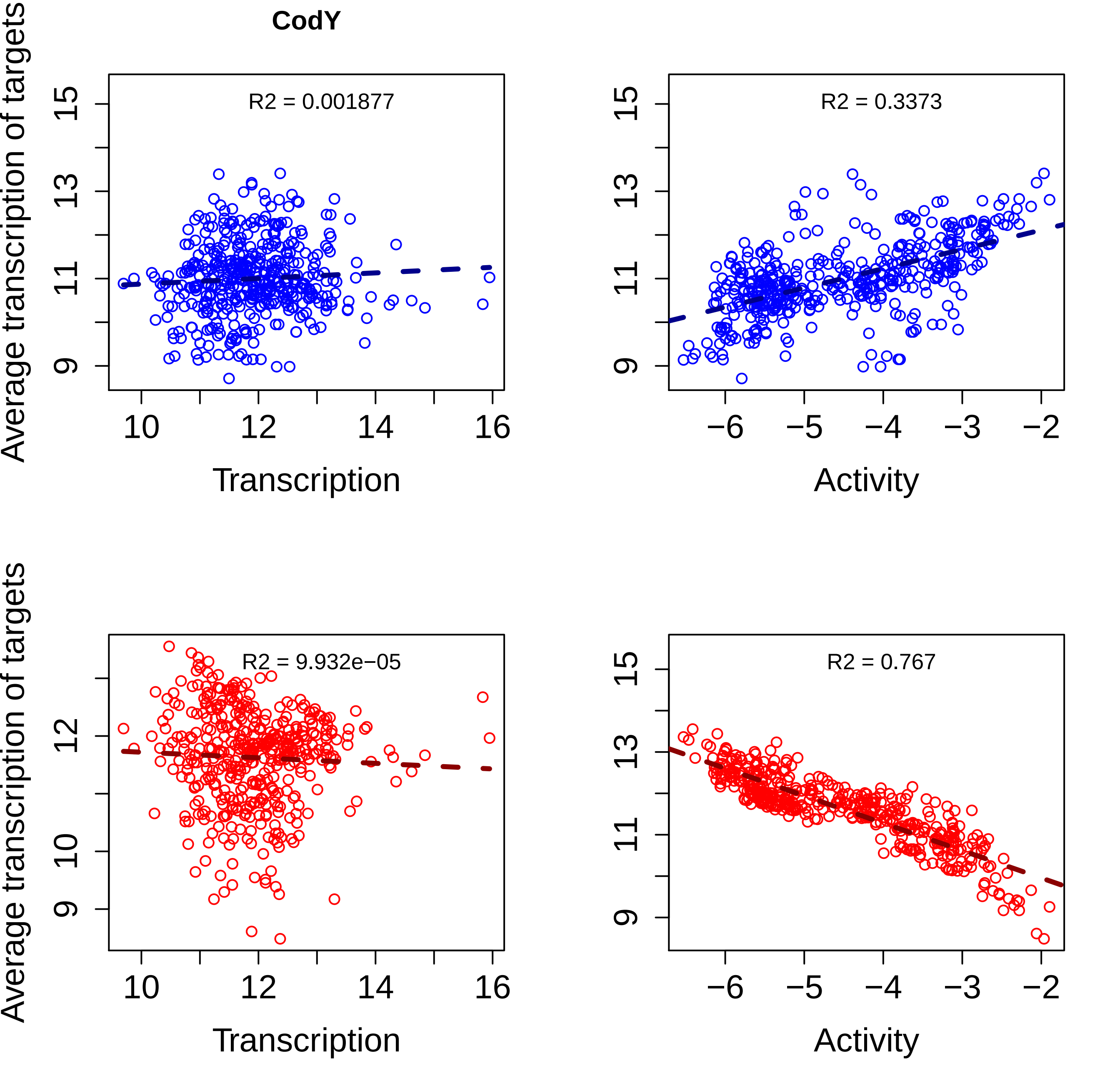

CodY
R2 = 0.001877
R2 = 0.3373
R2 = 9.932e−05
R2 = 0.767
Average transcription of targets
Transcription
Activity
15
13
11
9
12
10
16
14
−6
−5
−4
−3
−2

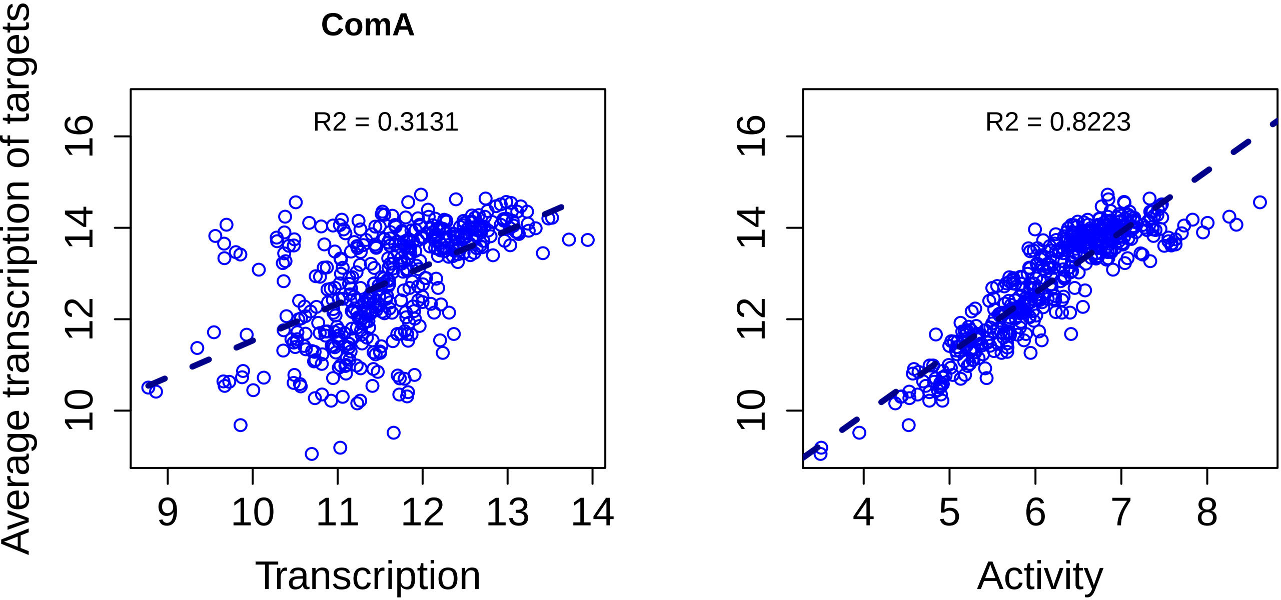
ComA
R2 = 0.3131
R2 = 0.8223
Average transcription of targets
Transcription
Activity
10
12
14
16
9
10
11
12
13
14
4
5
6
7
8

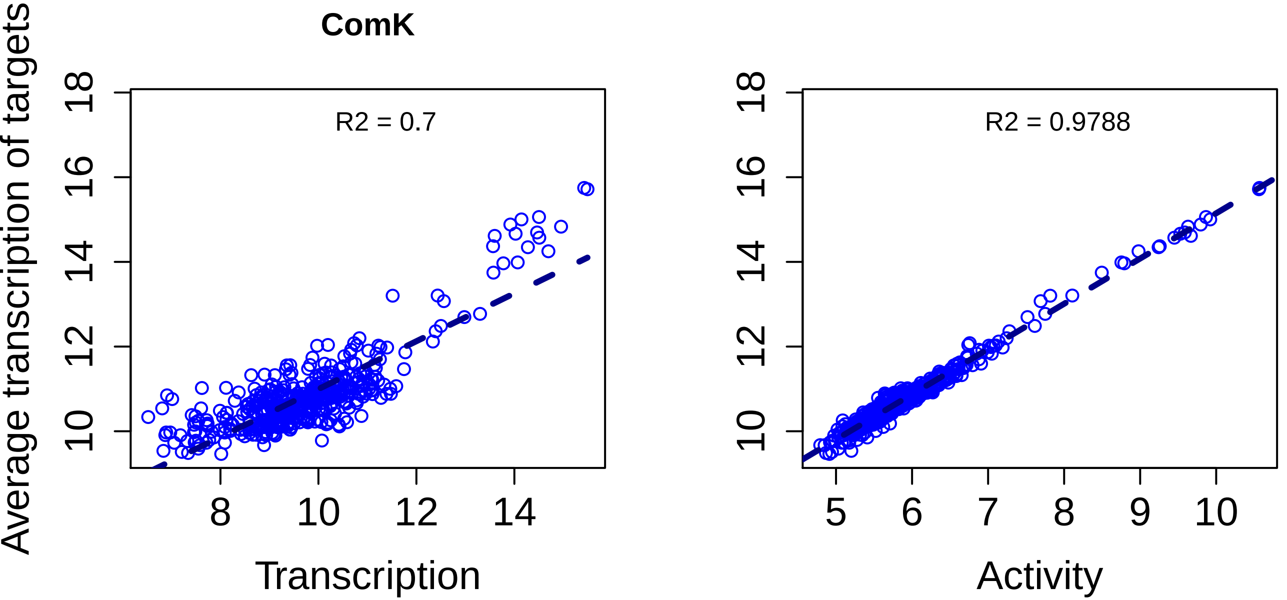

ComK
R2 = 0.7
R2 = 0.9788
Average transcription of targets
Transcription
Activity
10
12
14
16
18
8
10
12
14
5
6
7
8
9
10

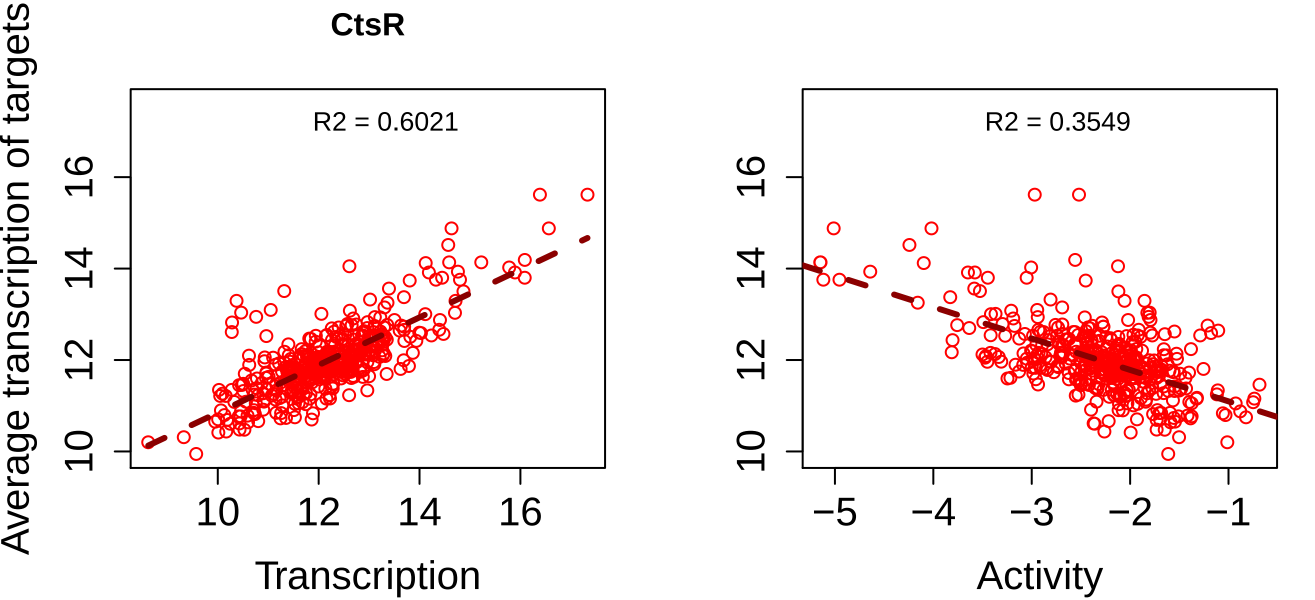

CtsR
R2 = 0.6021
R2 = 0.3549
Average transcription of targets
Transcription
Activity
10
12
14
16
−5
−4
−3
−2
−1

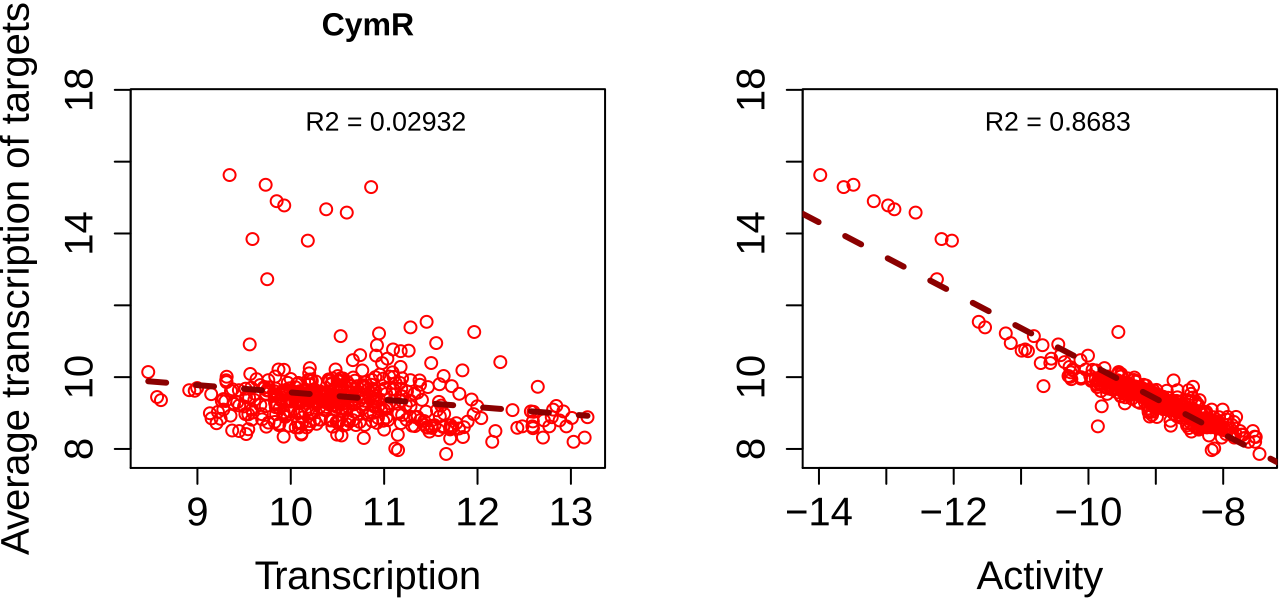
CymR
R2 = 0.02932
R2 = 0.8683
Average transcription of targets
8
10
14
18
9
10
11
12
13
Transcription
−14
−12
−10
−8
Activity

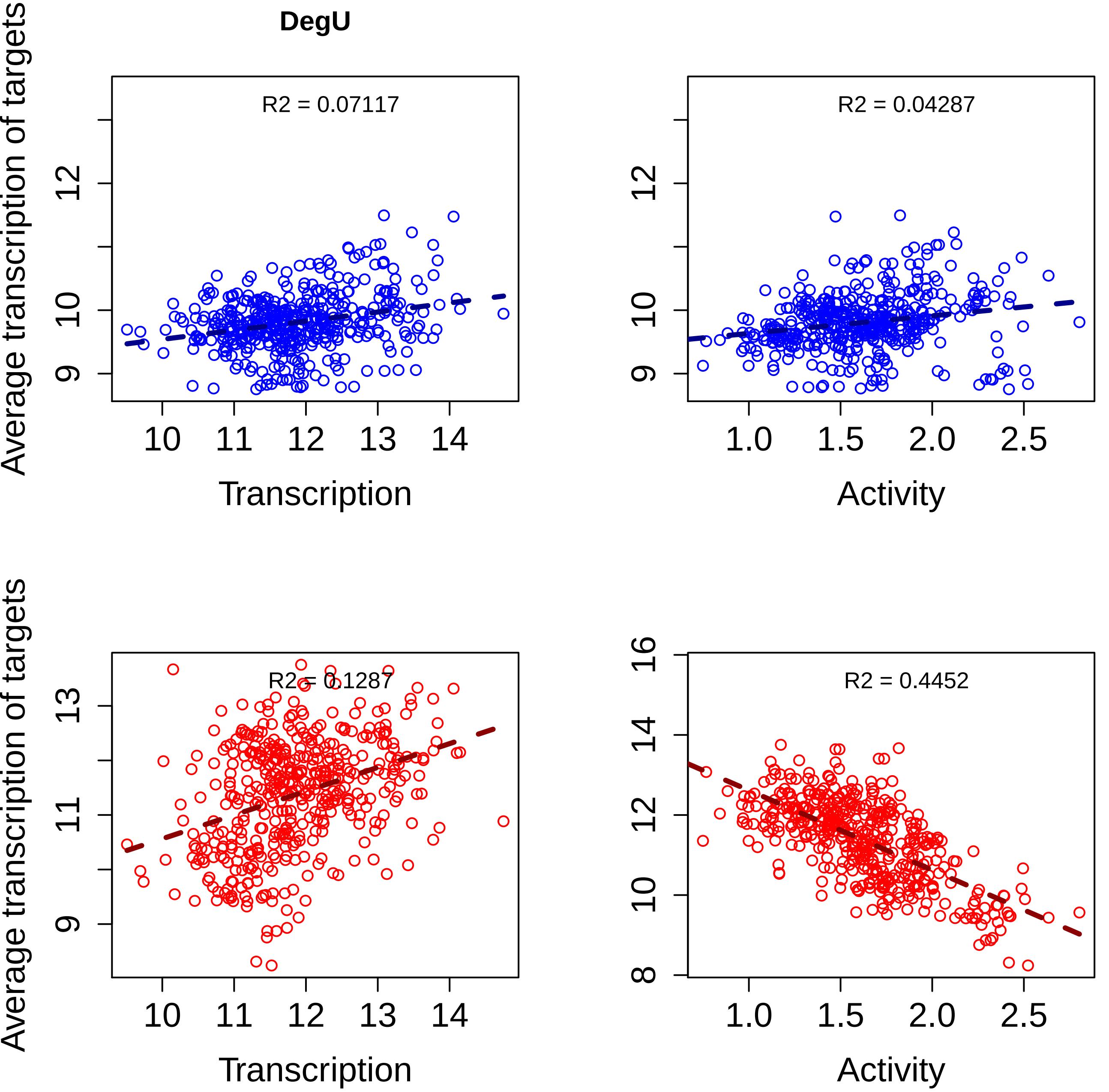

DegU
R2 = 0.07117
R2 = 0.04287
R2 = 0.1287
R2 = 0.4452
Average transcription of targets
Transcription
Activity

**ExuR**

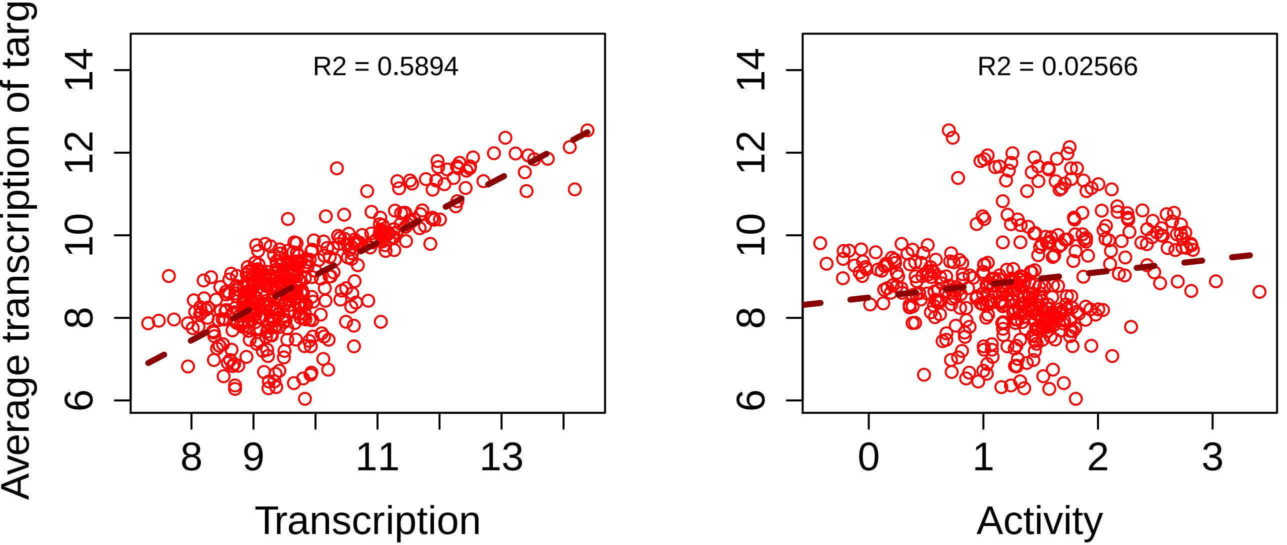

Average transcription of targets
R2 = 0.5894
14
12
10
8
6
8
9
11
13
Transcription
R2 = 0.02566
0
1
2
3
Activity

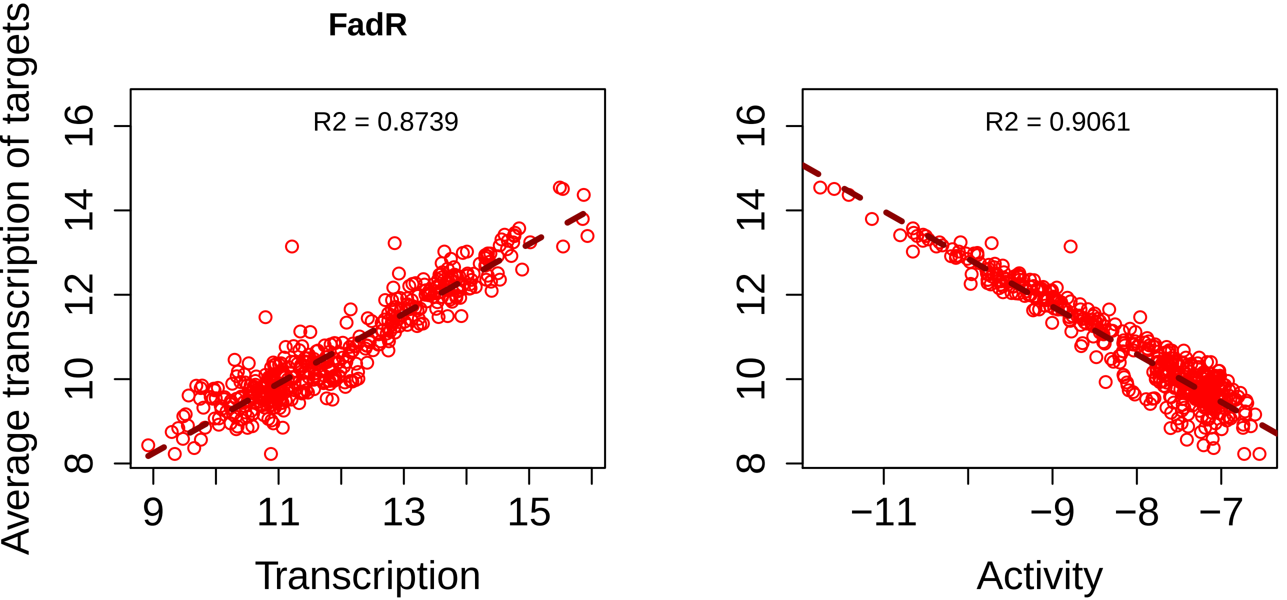

FadR
Average transcription of targets
R2 = 0.8739
16
14
12
10
8
9
11
13
15
Transcription
R2 = 0.9061
16
14
12
10
8
−11
−9
−8
−7
Activity

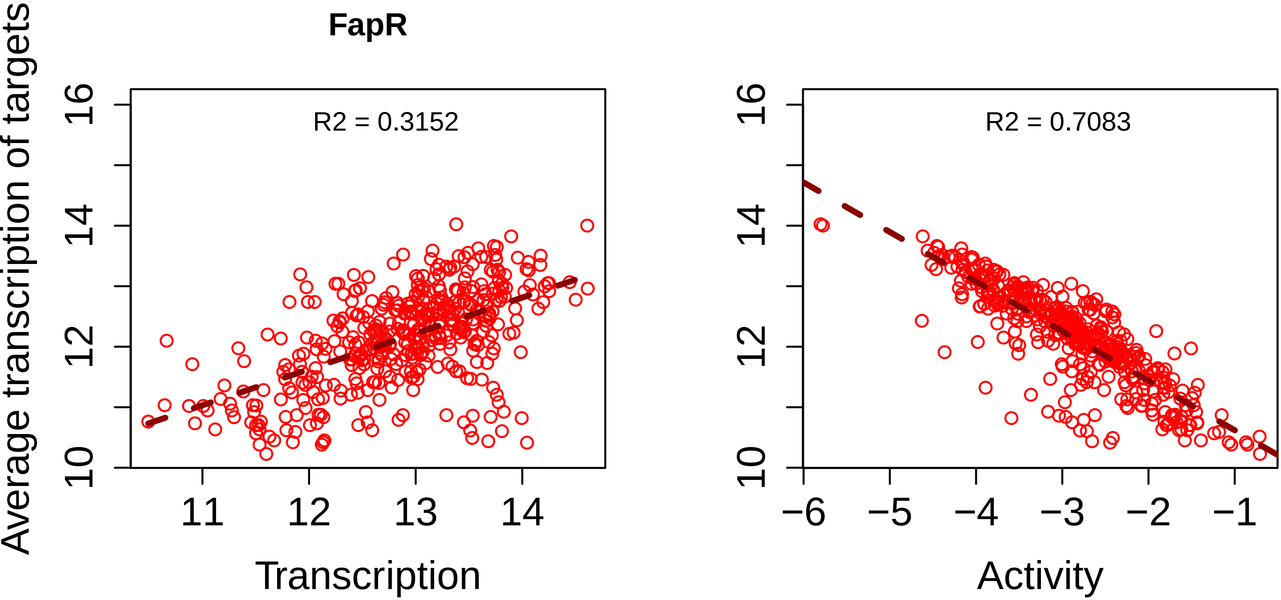
FapR
R2 = 0.3152
R2 = 0.7083
Average transcription of targets
Transcription
Activity
10
12
14
16
11
12
13
14
−6
−5
−4
−3
−2
−1

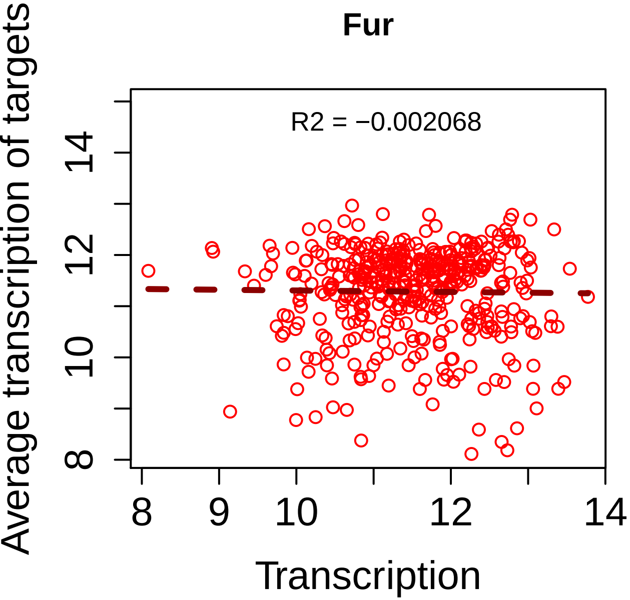
Fur
R2 = −0.002068
R2 = 0.9417
Average transcription of targets
Transcription
Activity
8
9
10
12
14
−11
−9
−8
−7

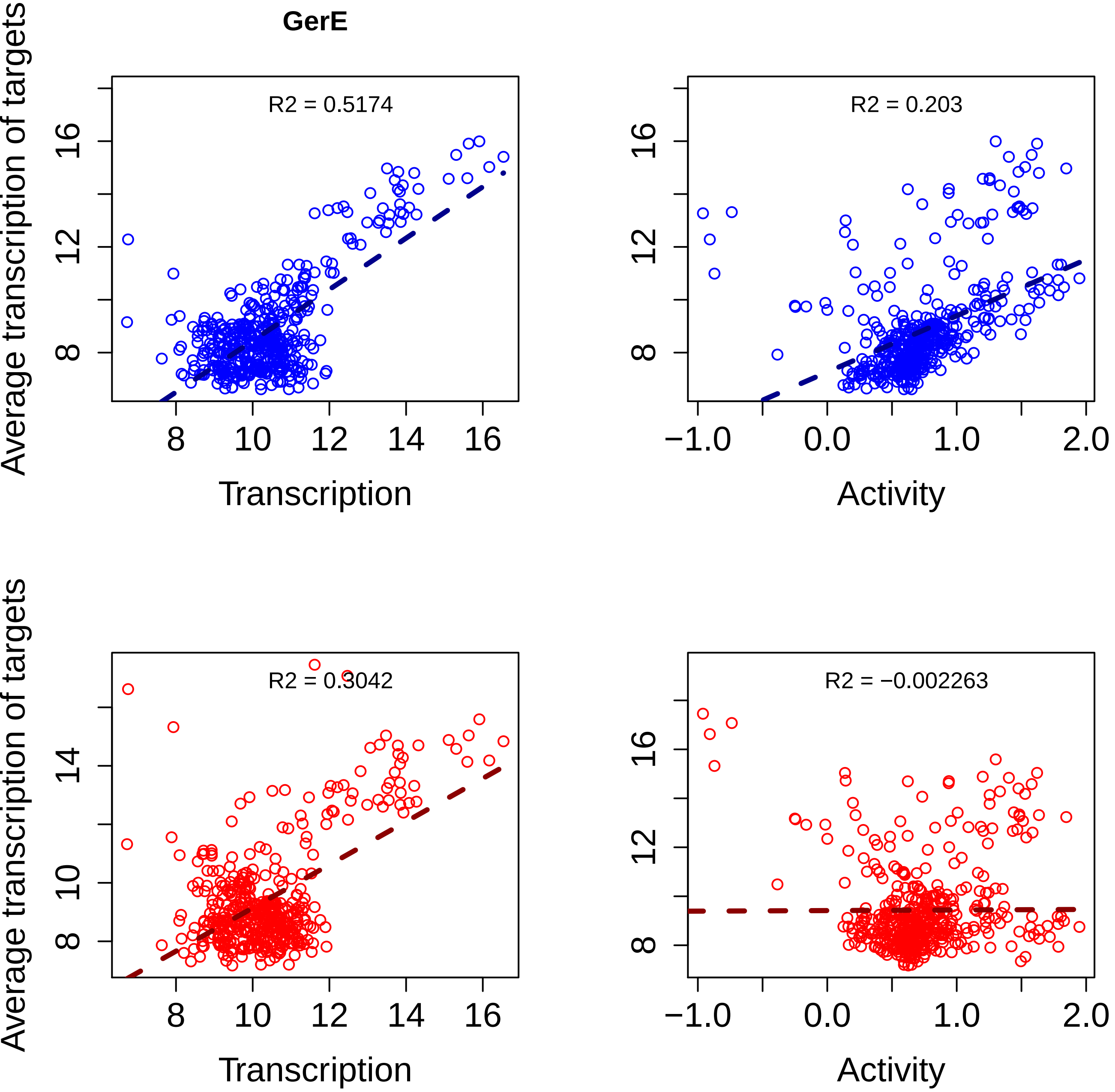

GerE
R2 = 0.5174
R2 = 0.203
R2 = 0.3042
R2 = −0.002263
Average transcription of targets
Transcription
Activity
8
10
12
14
16
−1.0
0.0
1.0
2.0

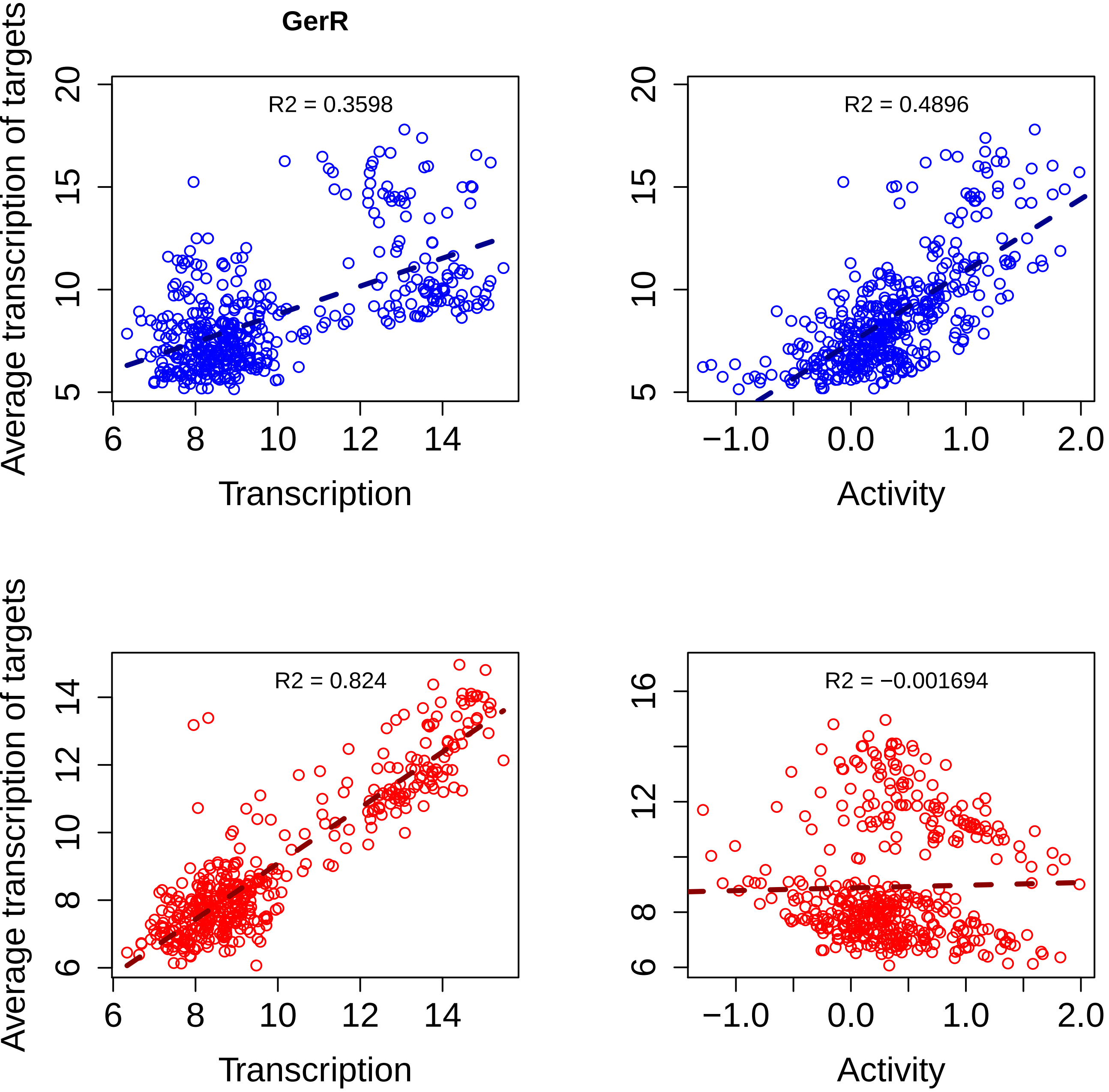
GerR
R2 = 0.3598
R2 = 0.4896
R2 = 0.824
R2 = −0.001694
Average transcription of targets
Transcription
Activity

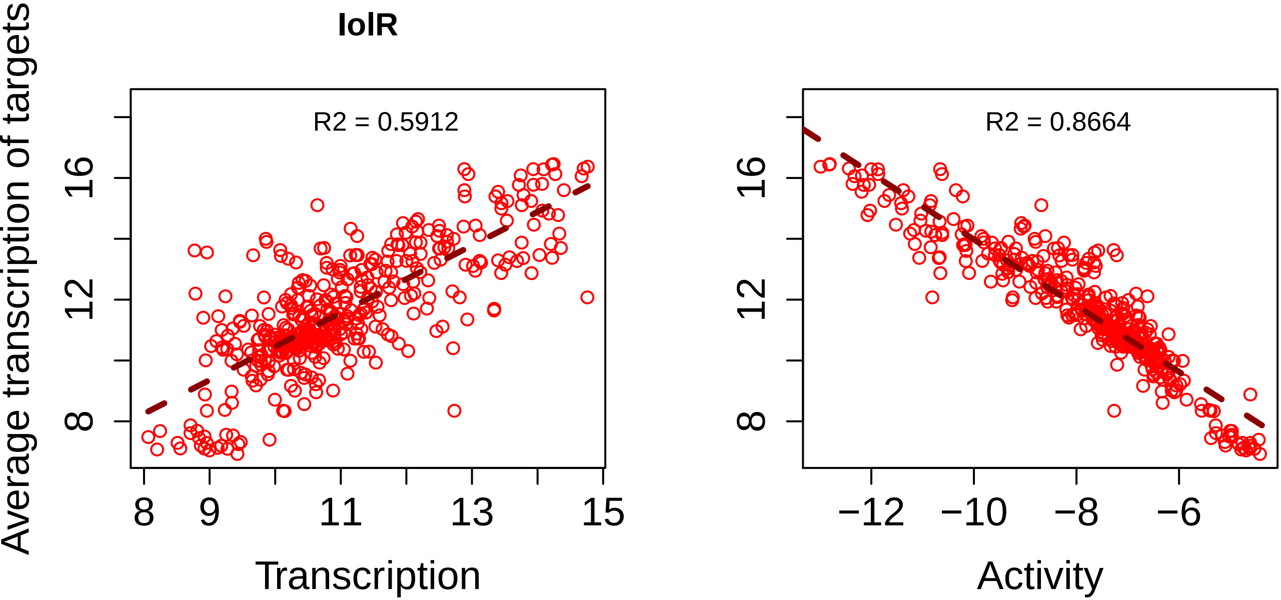

IolR
R2 = 0.5912
R2 = 0.8664
Average transcription of targets
Transcription
Activity
8
9
11
13
15
−12
−10
−8
−6
8
12
16

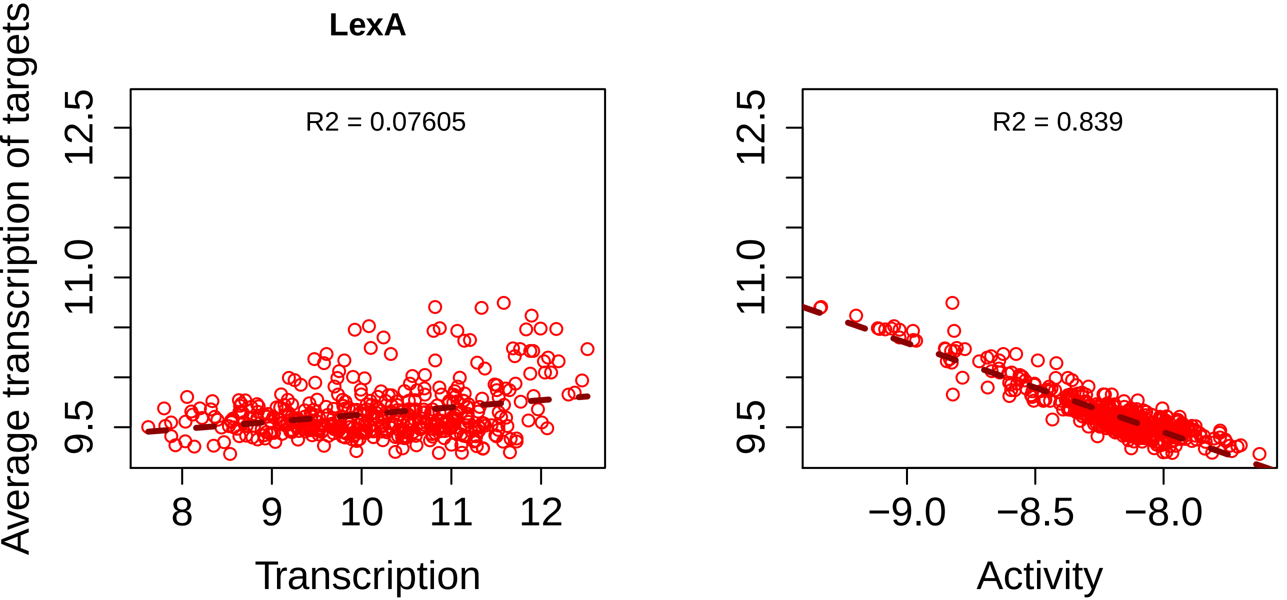

LexA
R2 = 0.07605
R2 = 0.839
Average transcription of targets
9.5
11.0
12.5
8
9
10
11
12
Transcription
−9.0
−8.5
−8.0
Activity

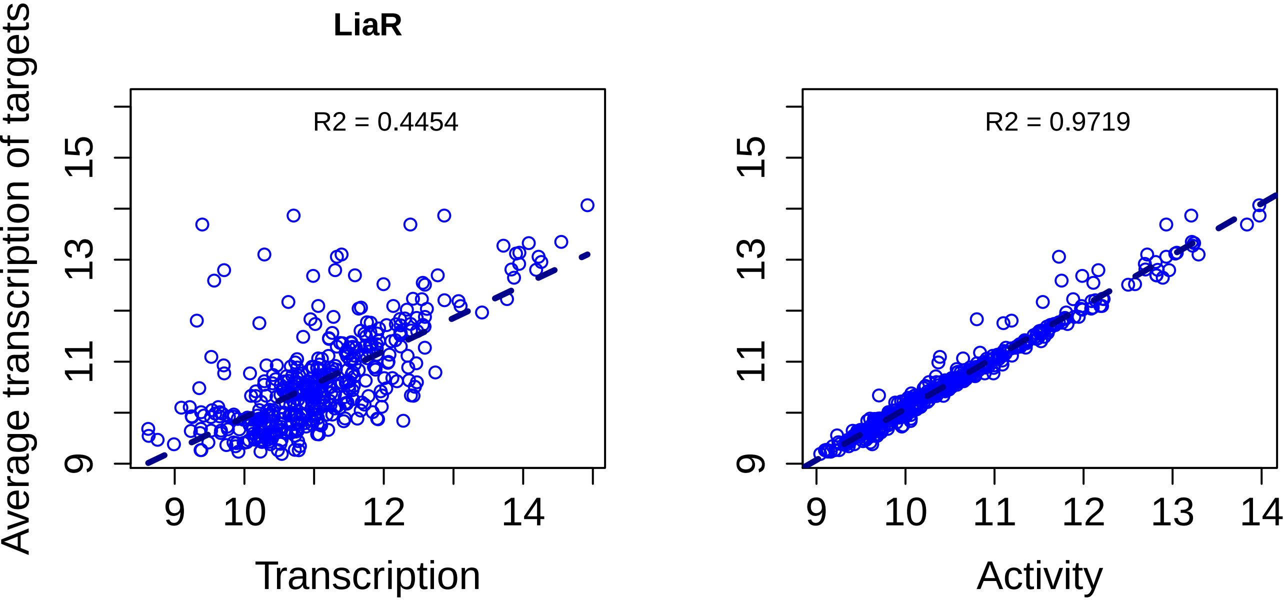

LiaR
R2 = 0.4454
R2 = 0.9719
Average transcription of targets
9
11
13
15
9
10
12
14
Transcription
9
10
11
12
13
14
Activity

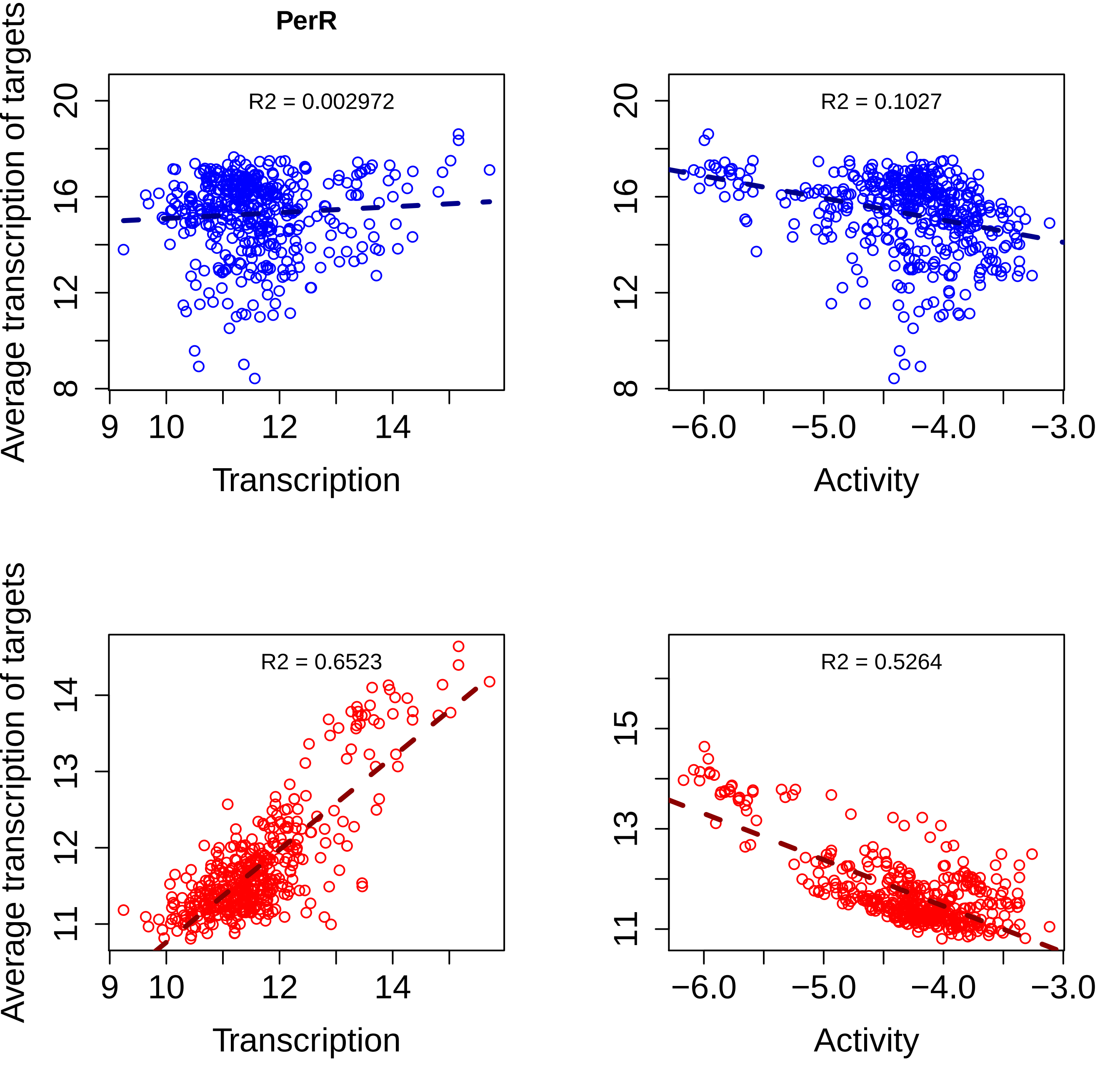

PerR
R2 = 0.002972
R2 = 0.1027
R2 = 0.6523
R2 = 0.5264
Average transcription of targets
Average transcription of targets
Transcription
Activity
Transcription
Activity
20
16
12
8
9
10
12
14
−6.0
−5.0
−4.0
−3.0
14
13
12
11
15

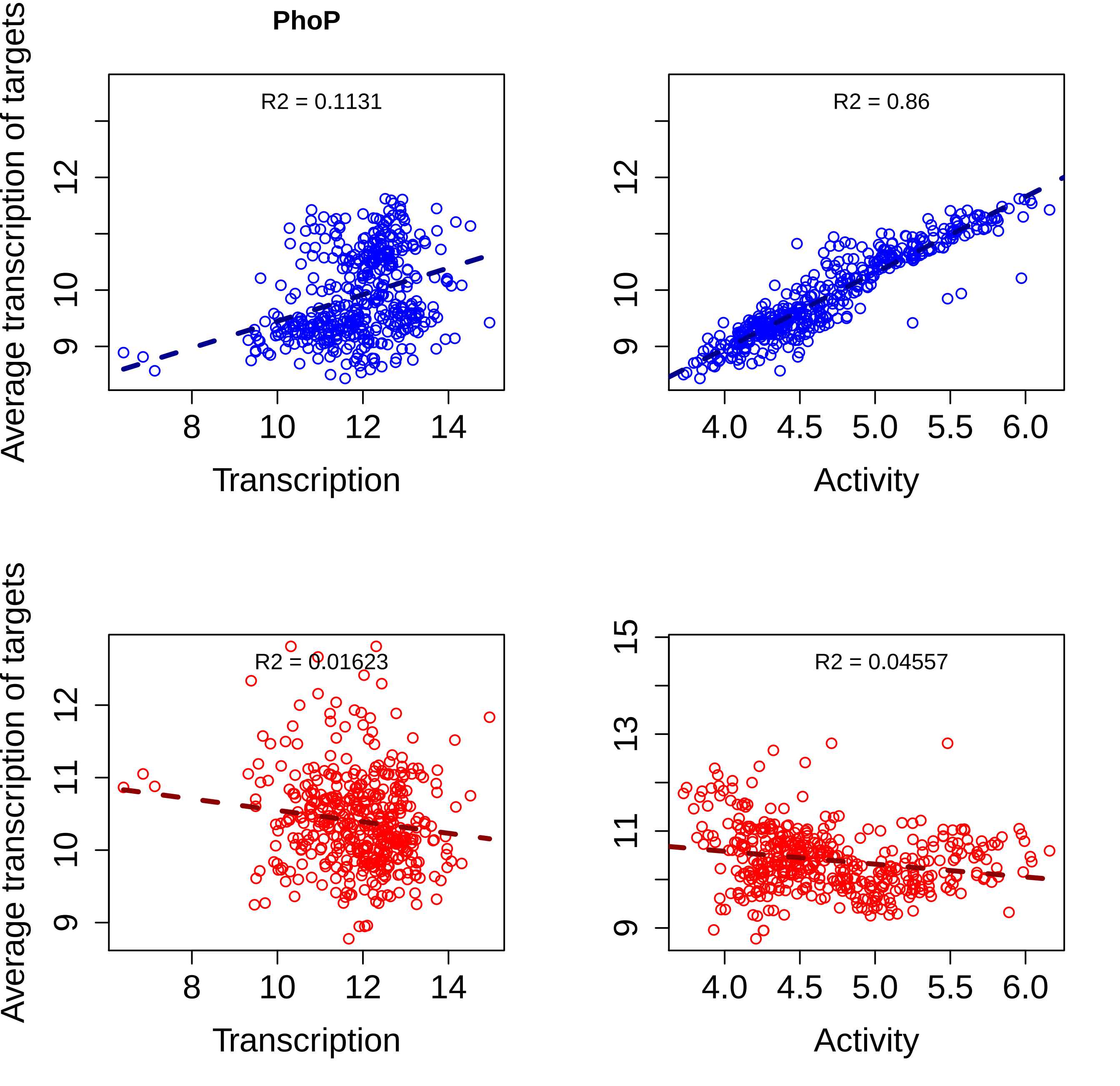
PhoP
R2 = 0.1131
R2 = 0.86
R2 = 0.01623
R2 = 0.04557
Average transcription of targets
Transcription
Activity
8
10
12
14
9
11
13
15
4.0
4.5
5.0
5.5
6.0

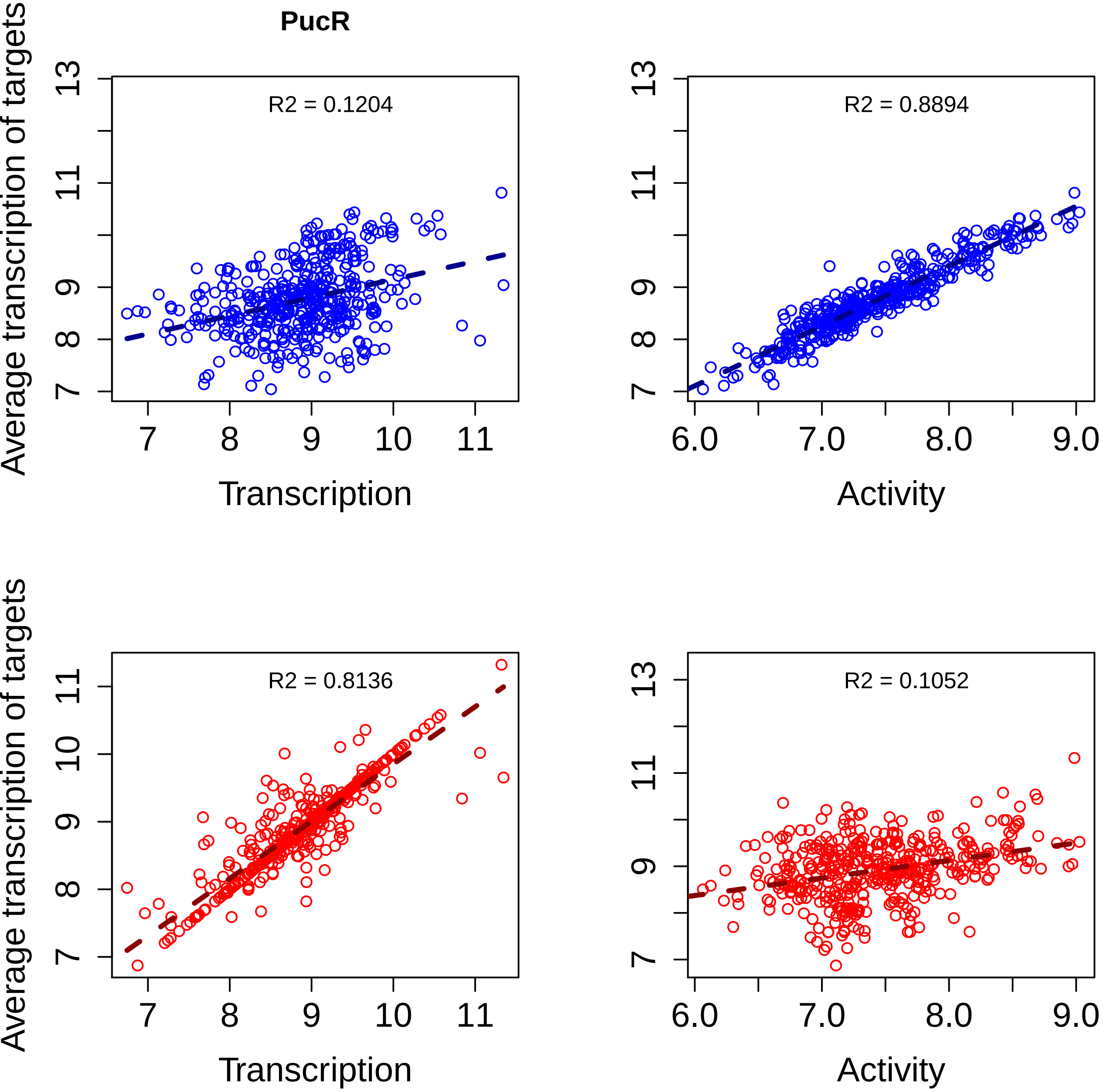

PucR
R2 = 0.1204
R2 = 0.8894
R2 = 0.8136
R2 = 0.1052
Average transcription of targets
Transcription
Activity
7
8
9
10
11
13
6.0
7.0
8.0
9.0

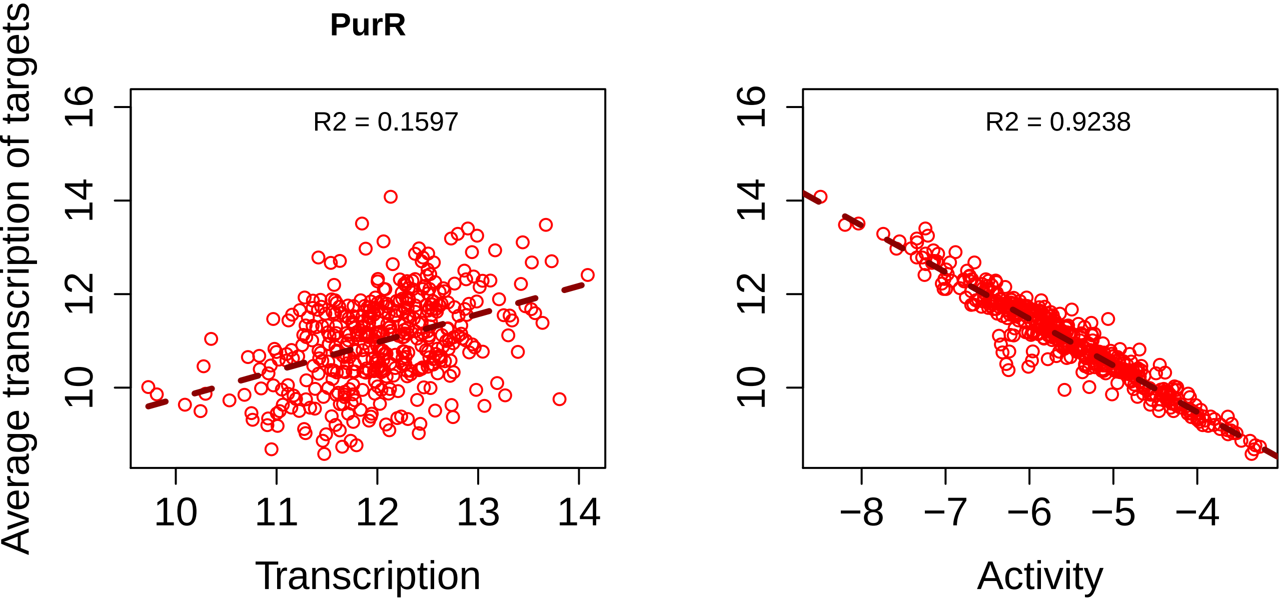

PurR
Average transcription of targets
R2 = 0.1597
16
14
12
10
10
11
12
13
14
Transcription
R2 = 0.9238
16
14
12
10
−8
−7
−6
−5
−4
Activity

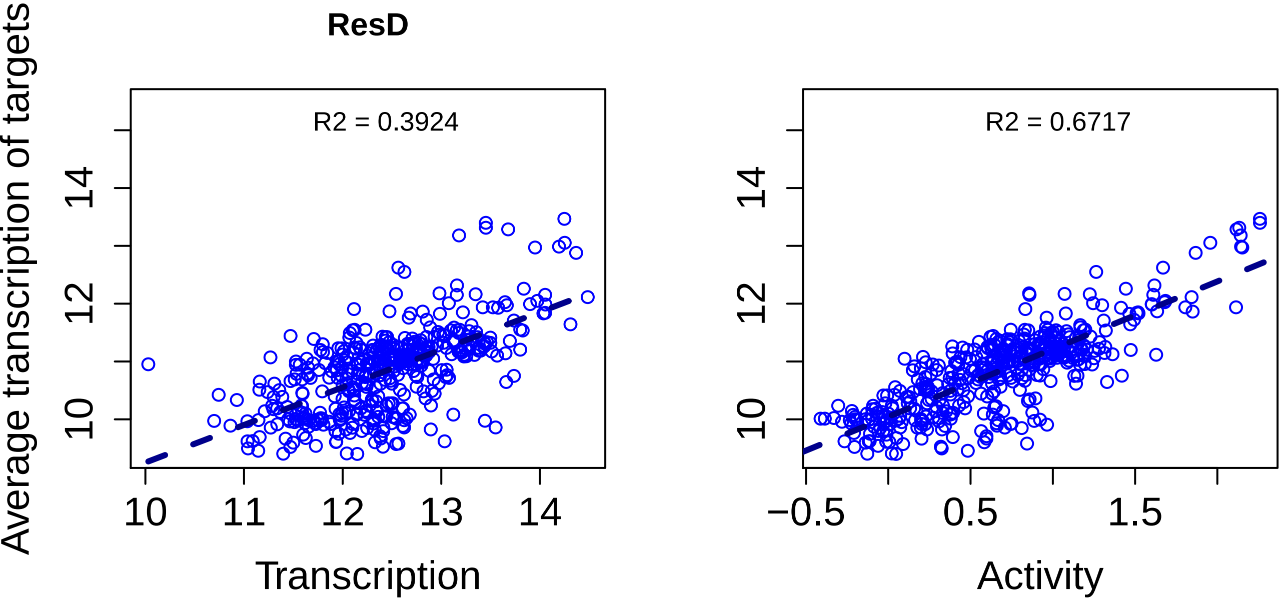

ResD
Average transcription of targets
R2 = 0.3924
10
12
14
10
11
12
13
14
Transcription
R2 = 0.6717
10
12
14
−0.5
0.5
1.5
Activity

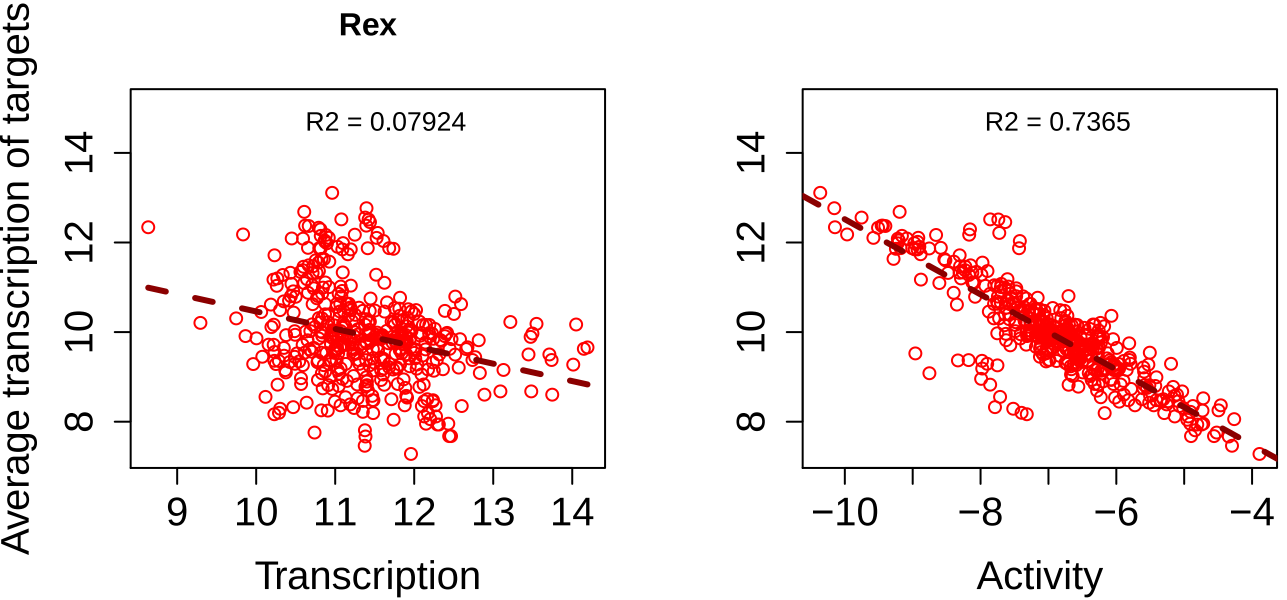

Rex
R2 = 0.07924
R2 = 0.7365
Average transcription of targets
8
10
12
14
9
10
11
12
13
14
Transcription
8
10
12
14
−10
−8
−6
−4
Activity

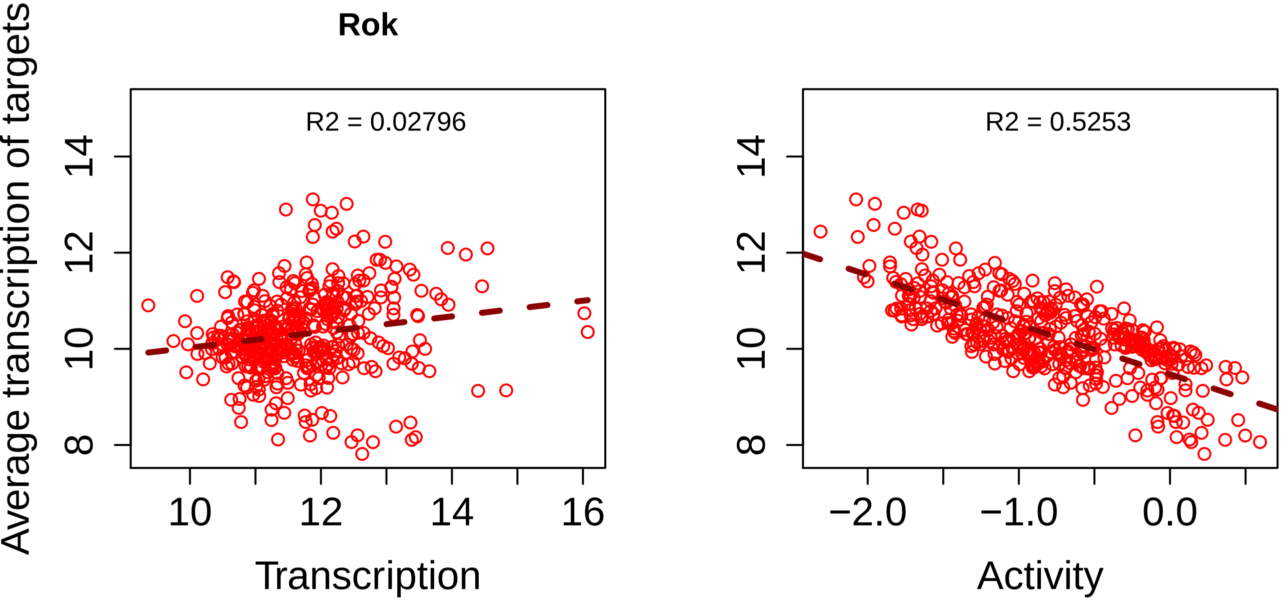

Rok
Average transcription of targets
R2 = 0.02796
14
12
10
8
10
12
14
16
Transcription
R2 = 0.5253
14
12
10
8
−2.0
−1.0
0.0
Activity

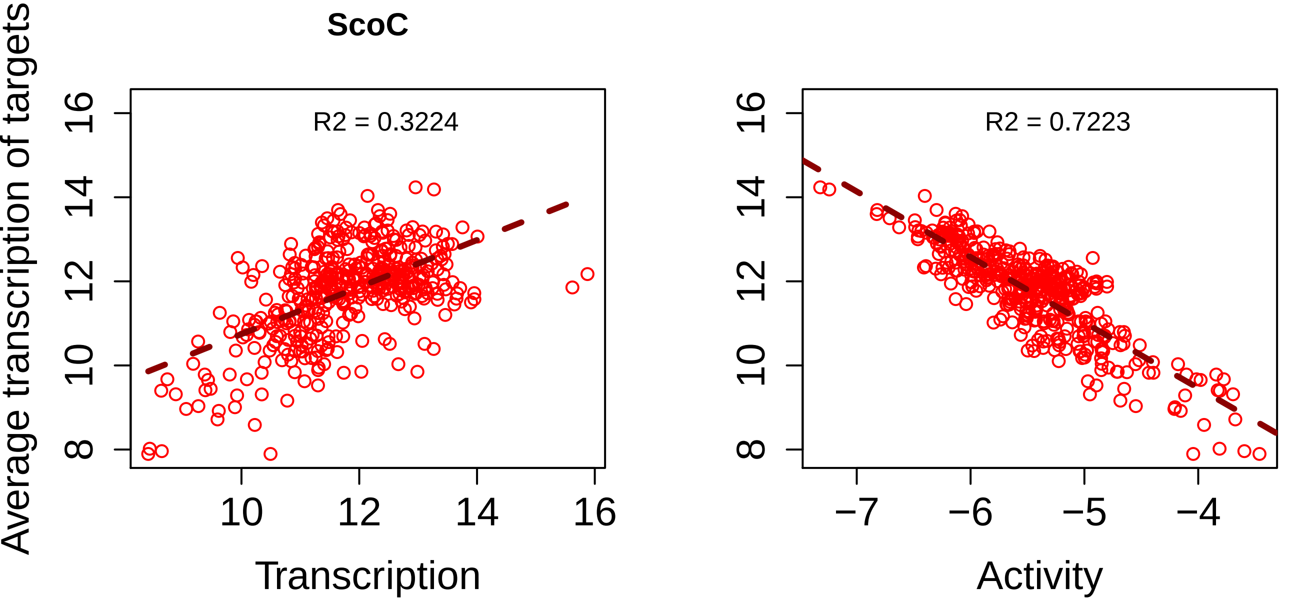

ScoC
R2 = 0.3224
R2 = 0.7223
Average transcription of targets
Transcription
Activity
8
10
12
14
16
−7
−6
−5
−4

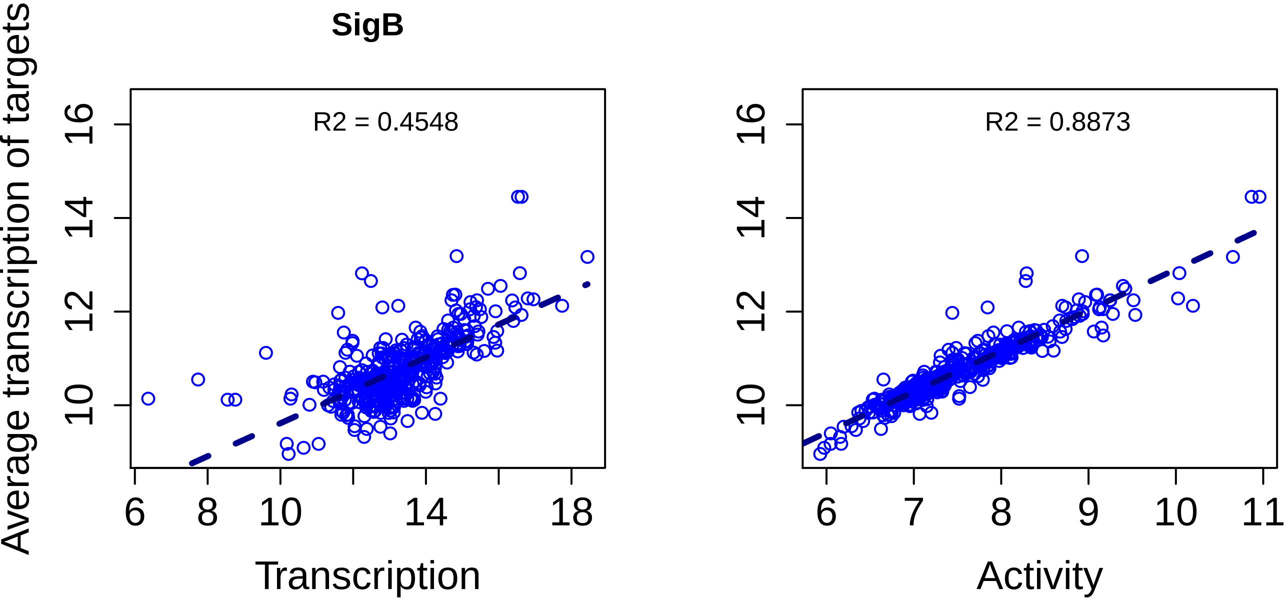
SigB
Average transcription of targets
R2 = 0.4548
R2 = 0.8873
16
14
12
10
6
8
10
14
18
Transcription
6
7
8
9
10
11
Activity

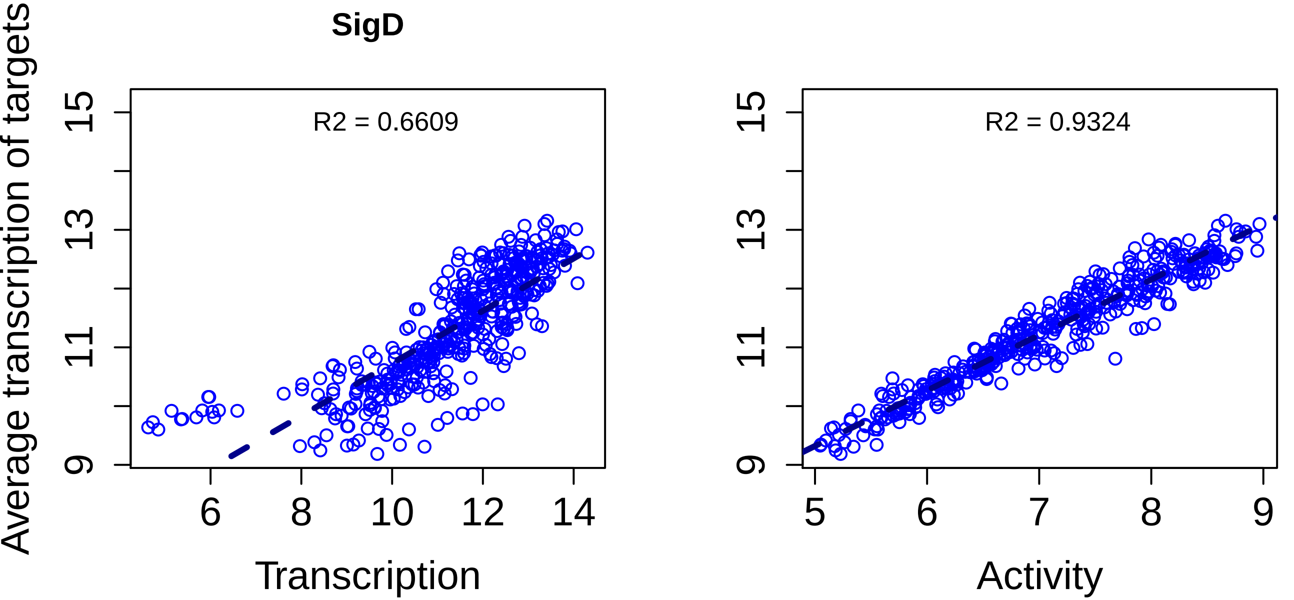
SigD
Average transcription of targets
R2 = 0.6609
Transcription
R2 = 0.9324
Activity
9
11
13
15
6
8
10
12
14
5
7

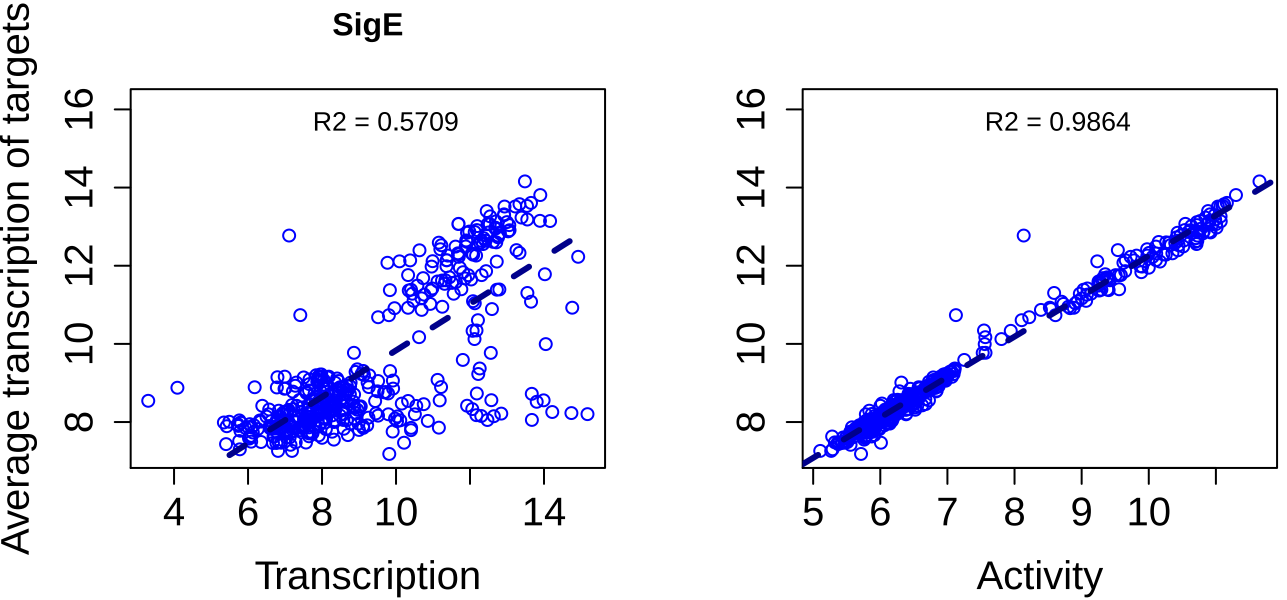
SigE
Average transcription of targets
R2 = 0.5709
Transcription
R2 = 0.9864
Activity
8
10
12
14
16
4
6
8
10
14
5
6
7
8
9
10

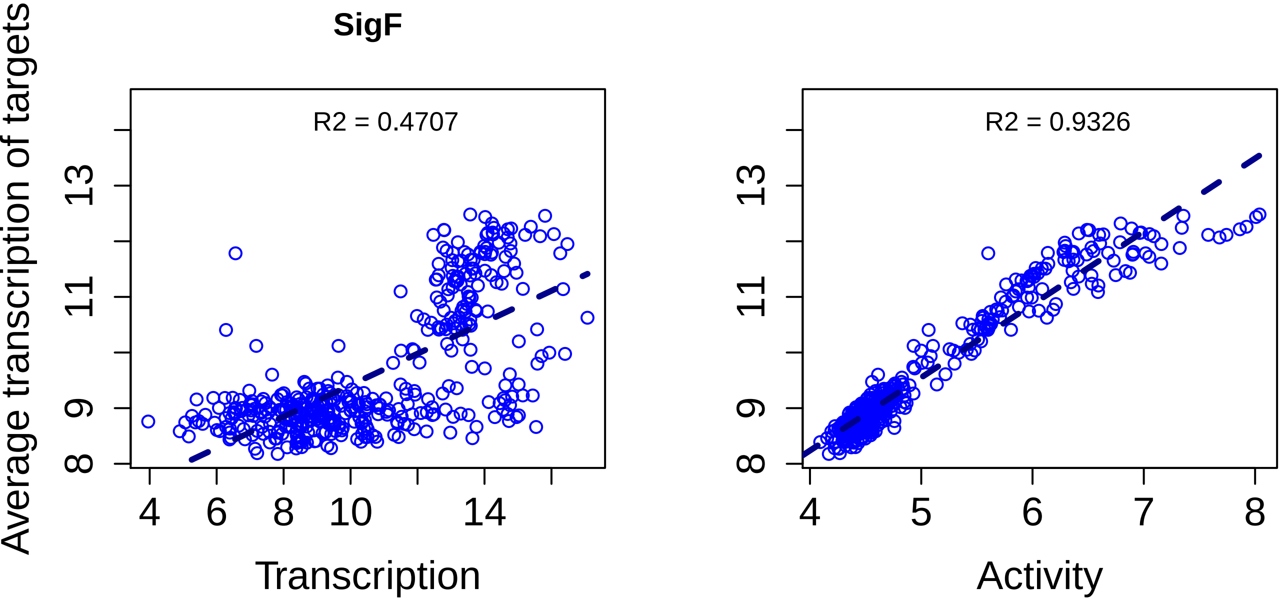
SigF
Average transcription of targets
R2 = 0.4707
8
9
11
13
4
6
8
10
14
Transcription
R2 = 0.9326
8
9
11
13
4
5
6
7
8
Activity

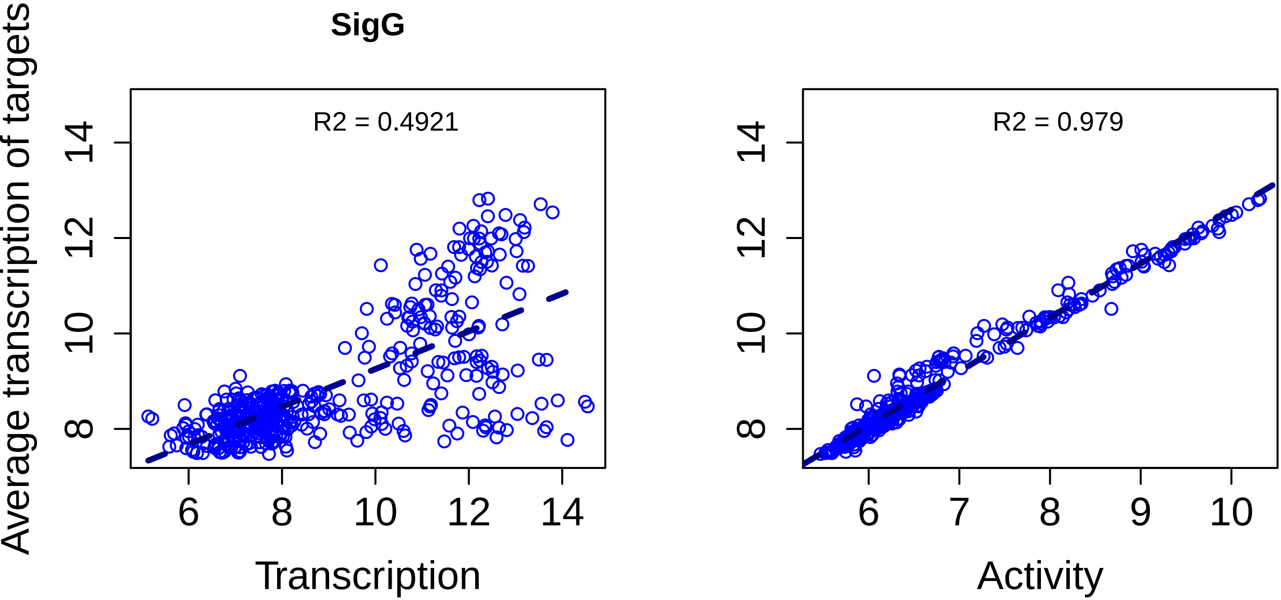
SigG
R2 = 0.4921
R2 = 0.979
Average transcription of targets
Transcription
Activity

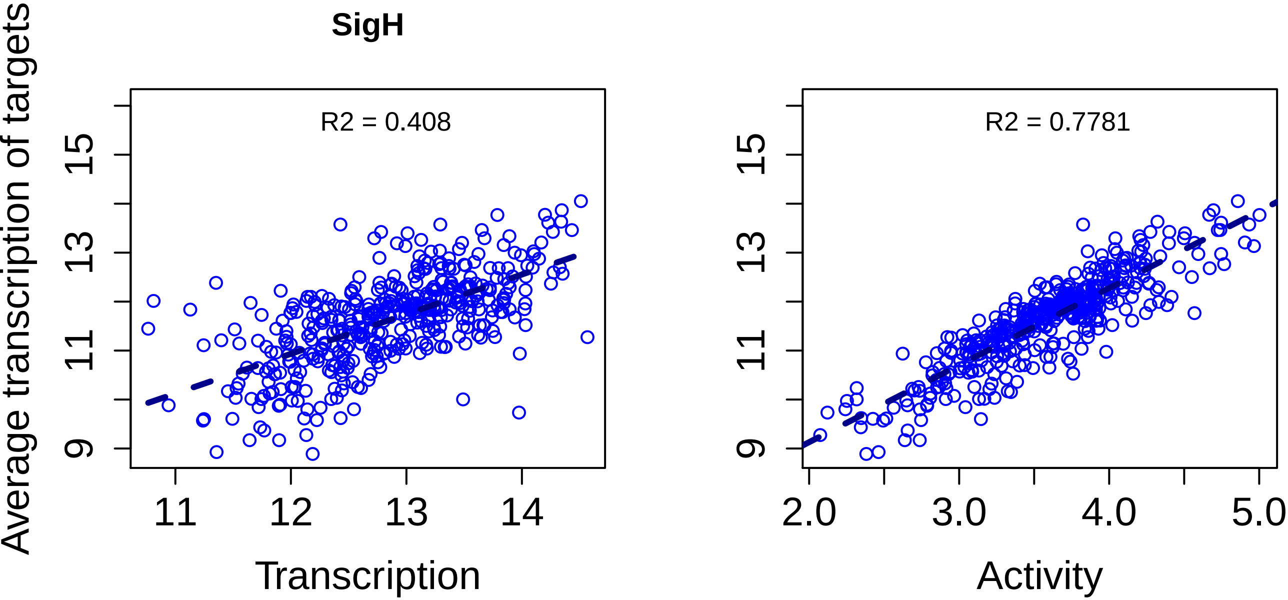
SigH
Average transcription of targets
R2 = 0.408
R2 = 0.7781
9
11
13
15
11
12
13
14
Transcription
2.0
3.0
4.0
5.0
Activity

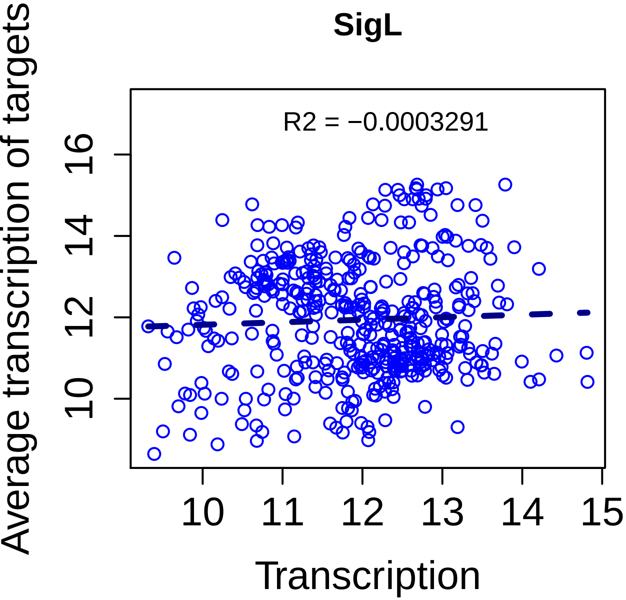
SigL
Average transcription of targets
R2 = −0.0003291
16
14
12
10
10
11
12
13
14
15
Transcription
R2 = 0.5915
16
14
12
10
4
5
6
7
8
Activity

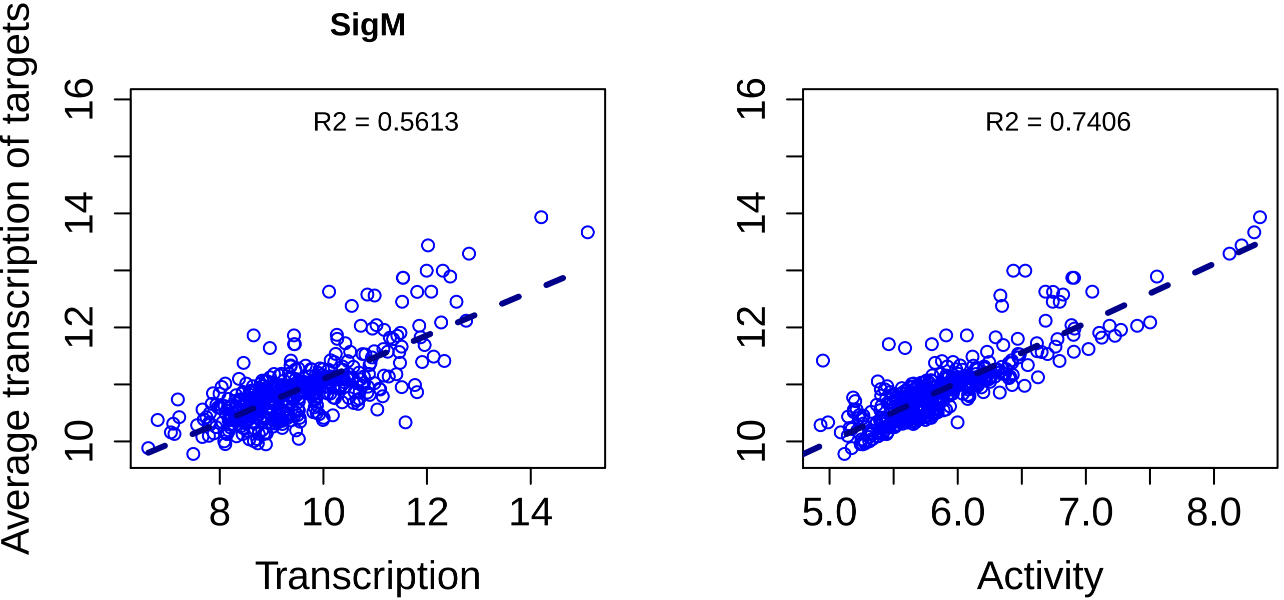

SigM
Average transcription of targets
R2 = 0.5613
R2 = 0.7406
10
12
14
16
8
10
12
14
5.0
6.0
7.0
8.0
Transcription
Activity

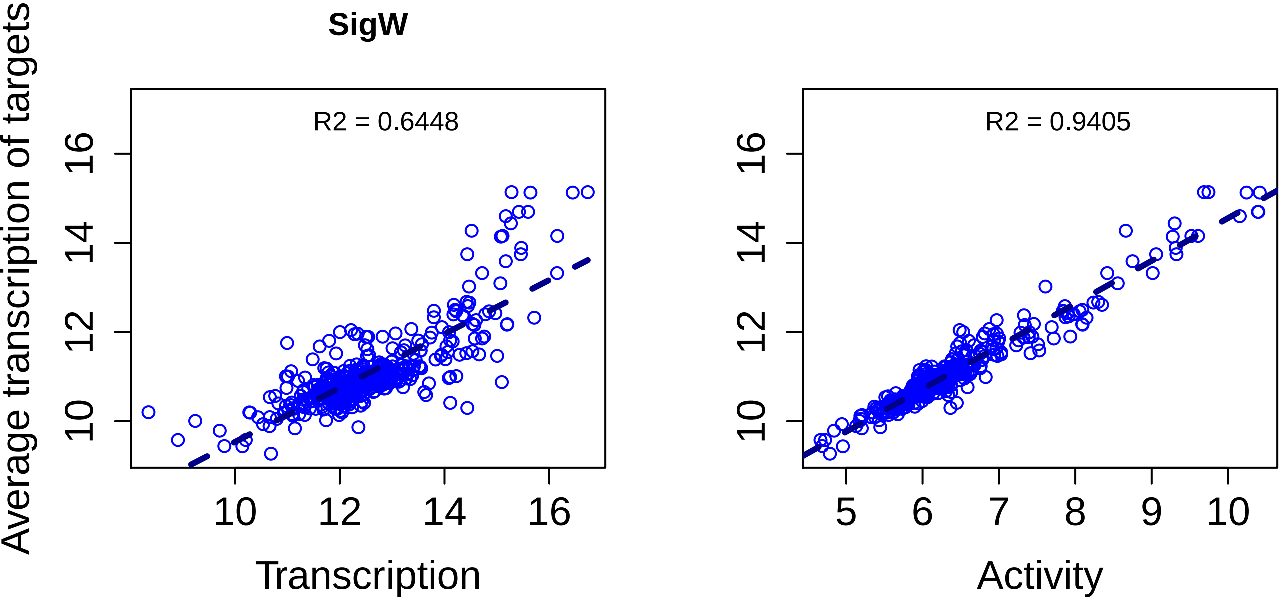

SigW
R2 = 0.6448
R2 = 0.9405
Average transcription of targets
Transcription
Activity
10
12
14
16
5
6
7
8
9
10

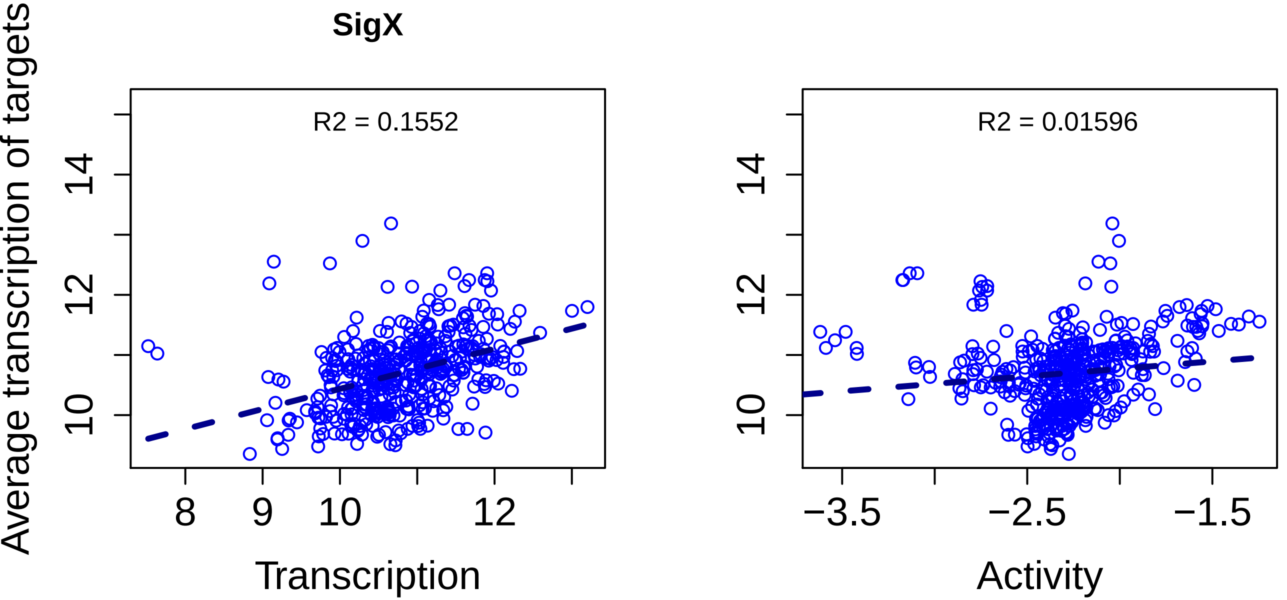
SigX
Average transcription of targets
R2 = 0.1552
14
12
10
8
9
10
12
Transcription
R2 = 0.01596
14
12
10
−3.5
−2.5
−1.5
Activity

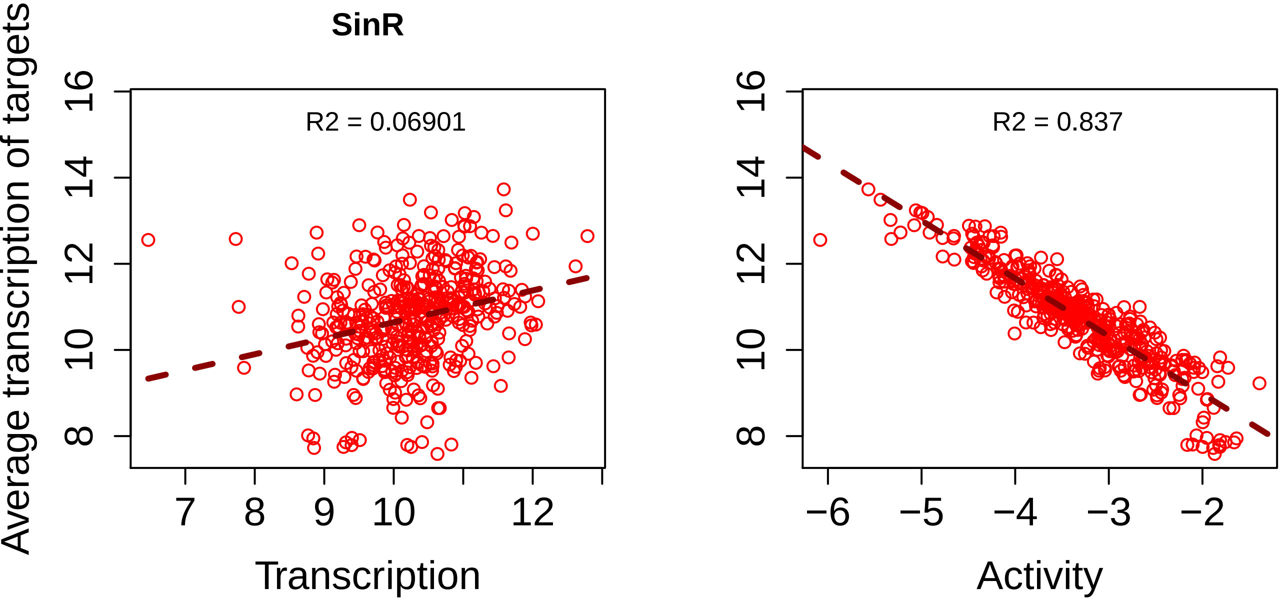
SinR
R2 = 0.06901
R2 = 0.837
Average transcription of targets
Transcription
Activity
16
14
12
10
8
7
8
9
10
12
−6
−5
−4
−3
−2

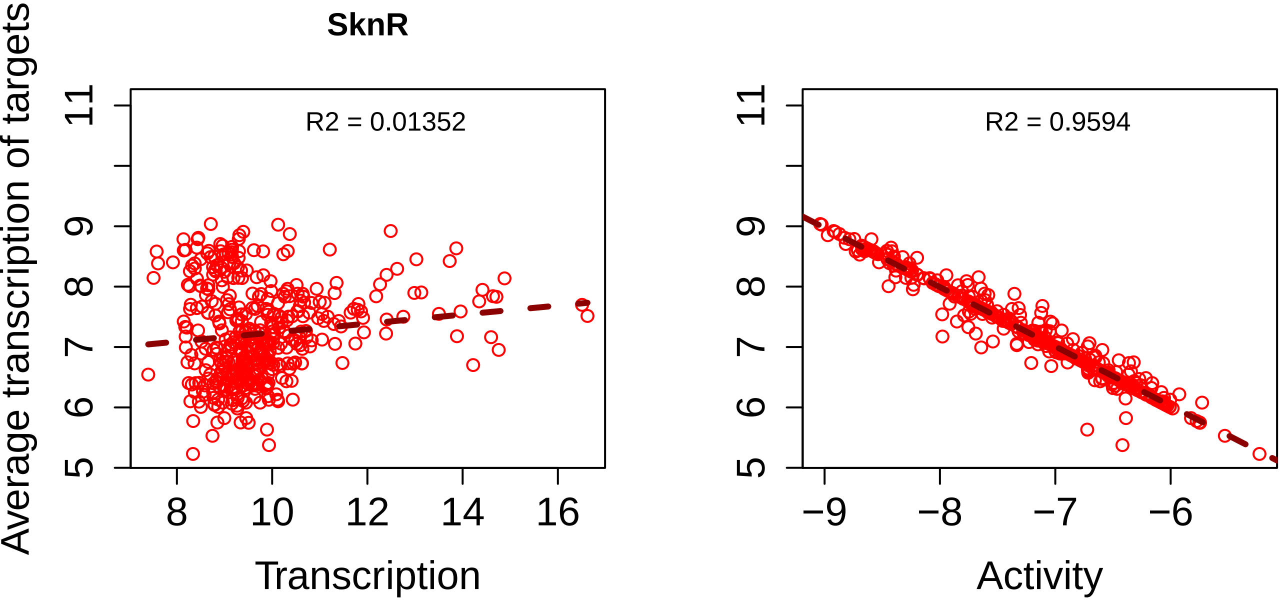
SknR
Average transcription of targets
R2 = 0.01352
Transcription
R2 = 0.9594
Activity
5
6
7
8
9
11
8
10
12
14
16
−9
−8
−7
−6

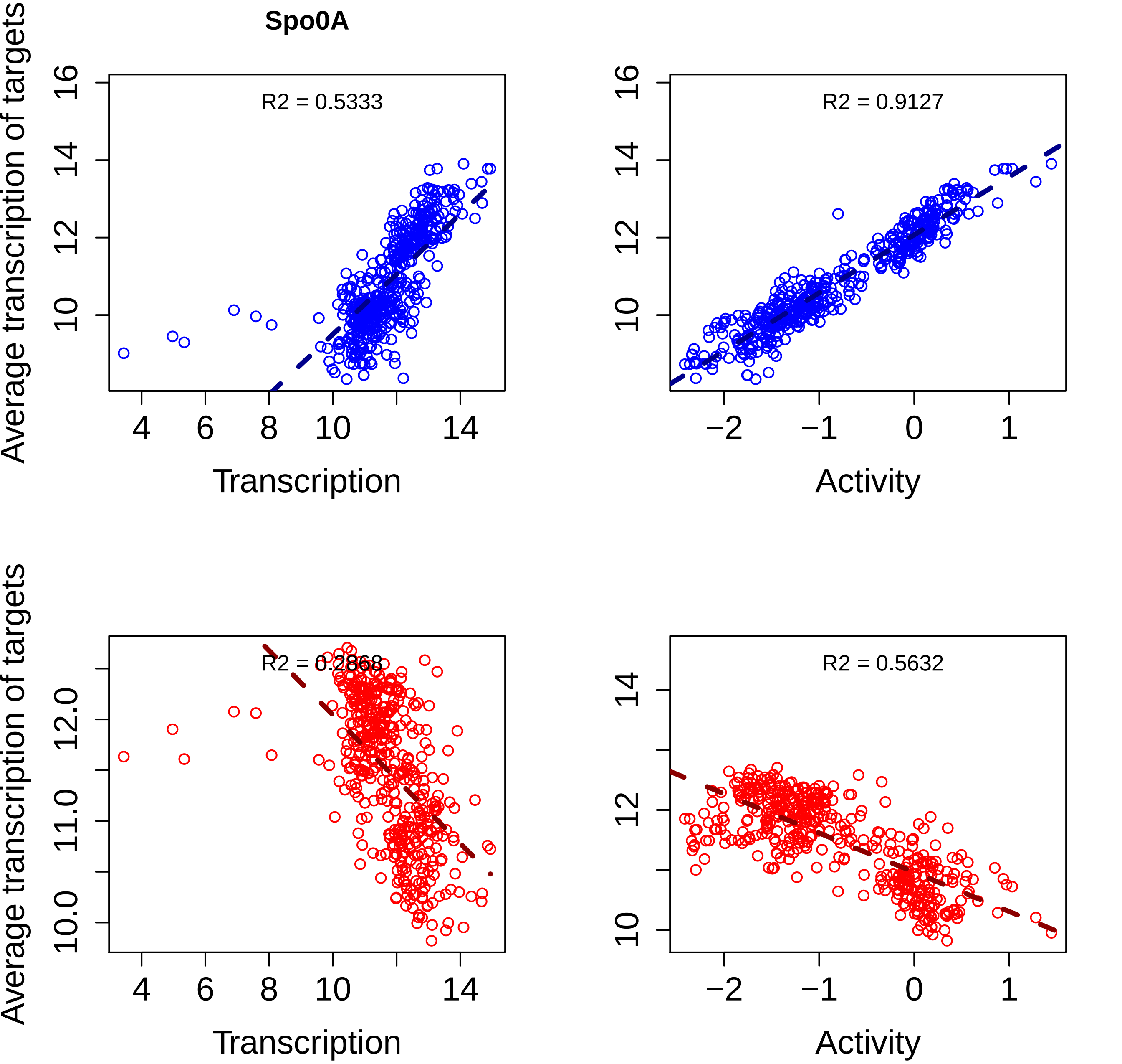

Spo0A
R2 = 0.5333
R2 = 0.9127
R2 = 0.2868
R2 = 0.5632
Average transcription of targets
Average transcription of targets
Transcription
Activity
Transcription
Activity

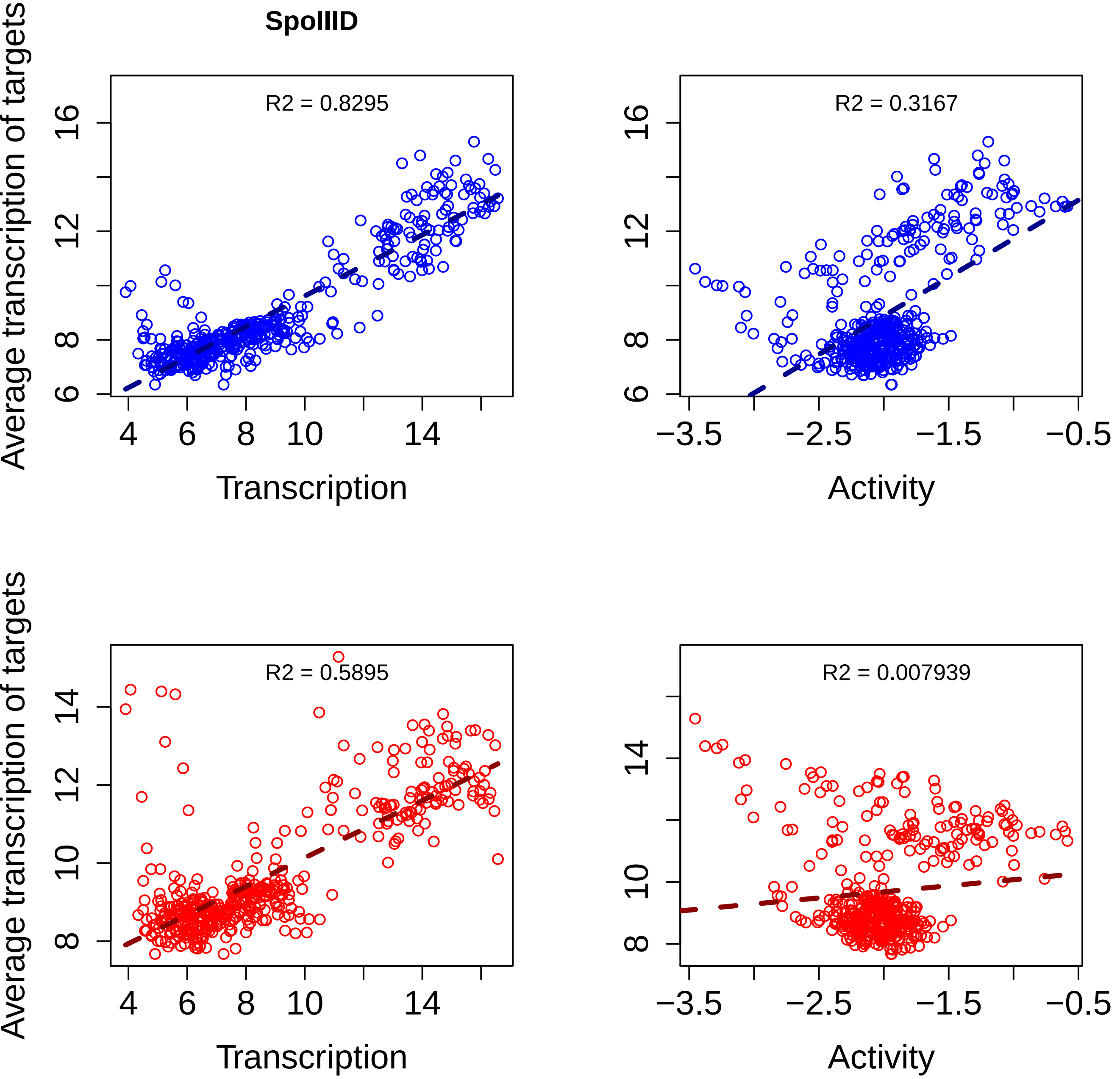
SpoIIID
Average transcription of targets
R2 = 0.8295
R2 = 0.3167
R2 = 0.5895
R2 = 0.007939
6
8
12
16
10
14
4
6
8
10
14
−3.5
−2.5
−1.5
−0.5
Transcription
Activity

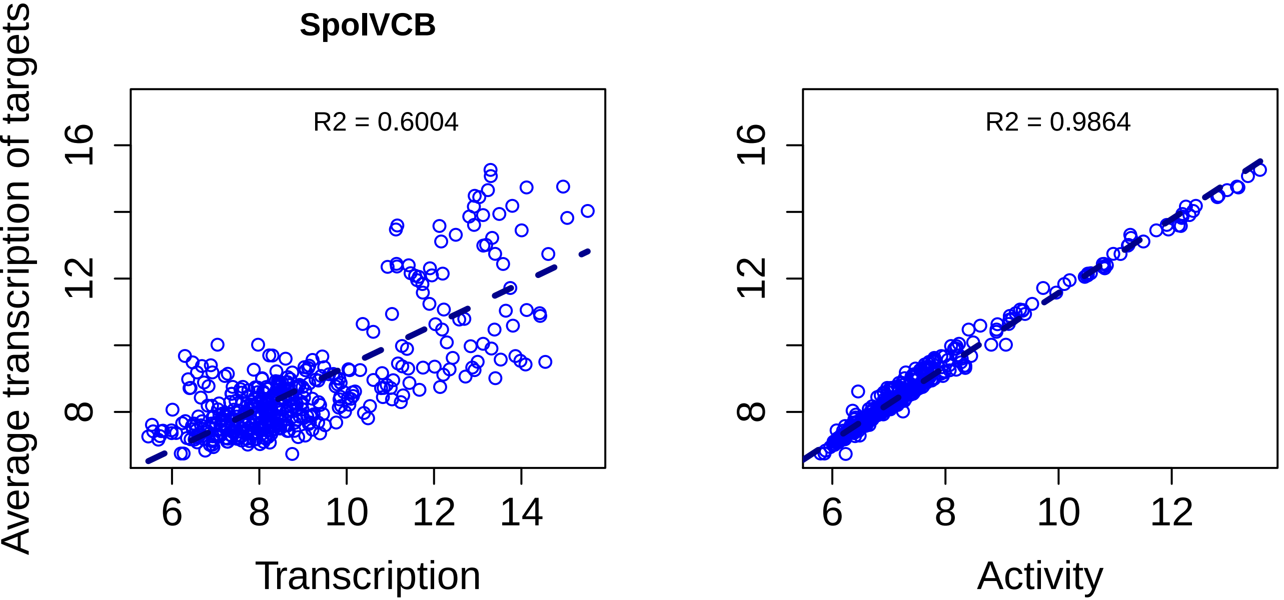

SpoIVCB
Average transcription of targets
R2 = 0.6004
16
12
8
6
8
10
12
14
Transcription
R2 = 0.9864
16
12
8
6
8
10
12
Activity

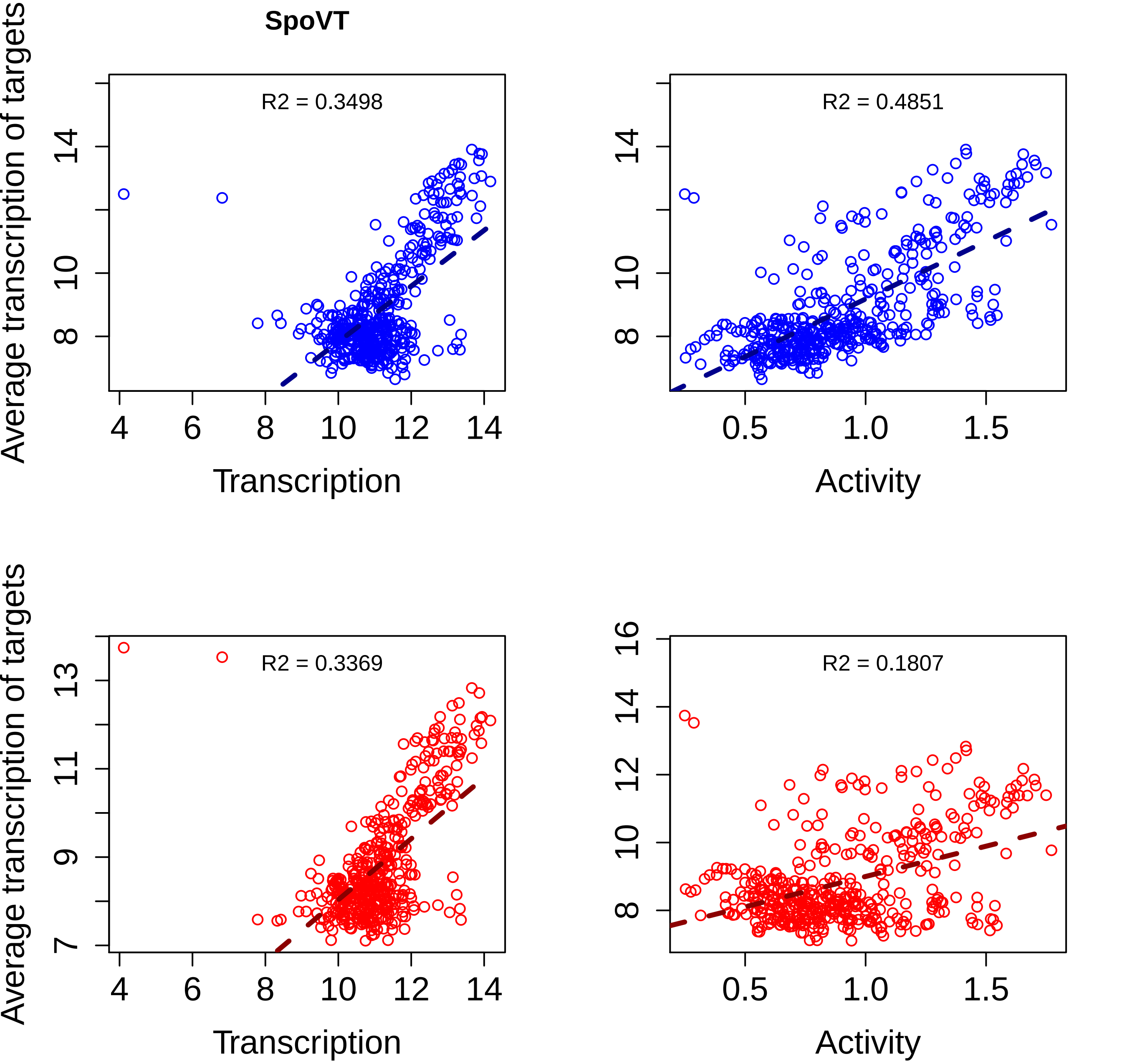
SpoVT
R2 = 0.3498
R2 = 0.4851
R2 = 0.3369
R2 = 0.1807
Average transcription of targets
Transcription
Activity
4
6
8
10
12
14
0.5
1.0
1.5
7
9
11
13
16

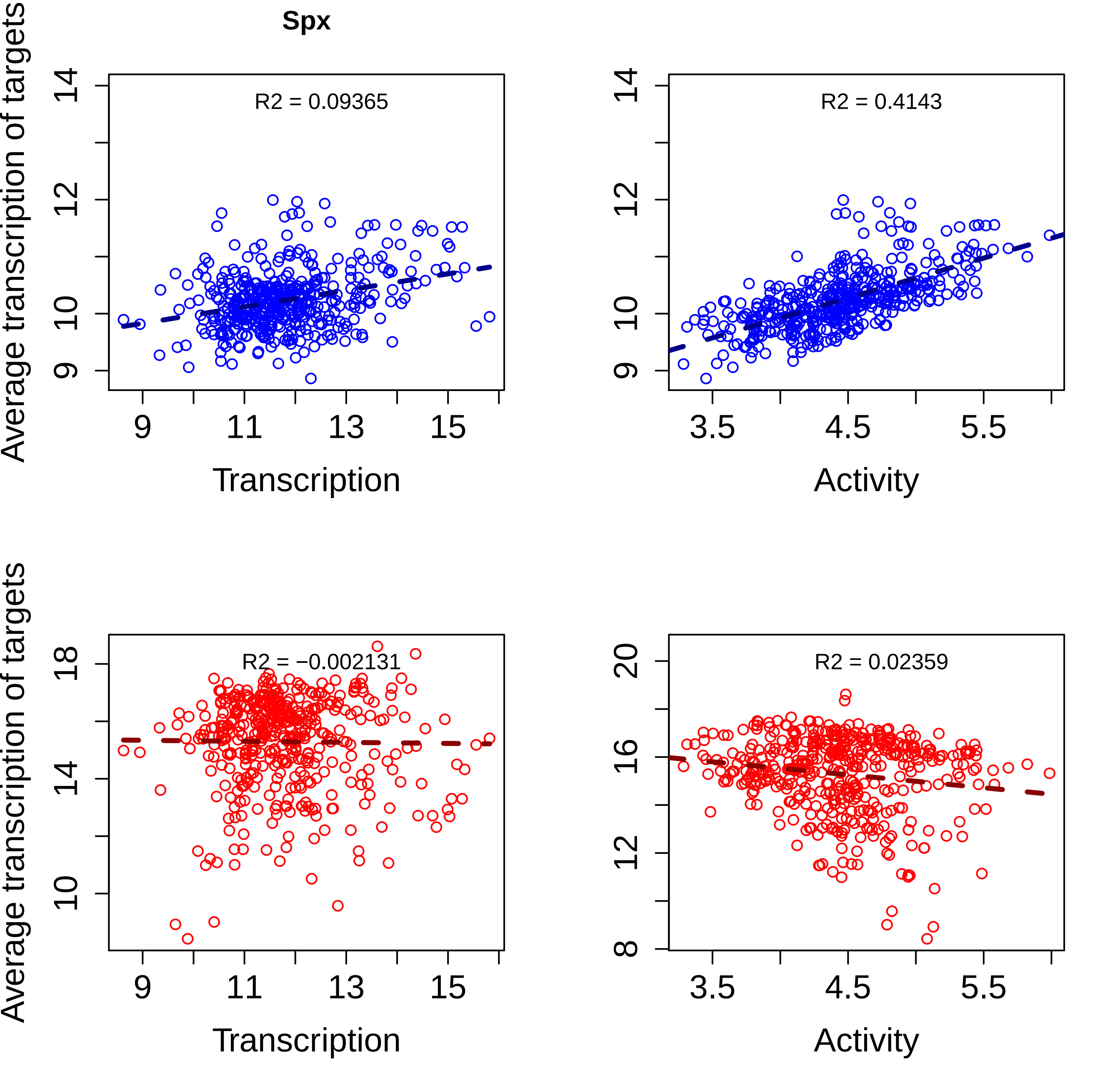

Spx
R2 = 0.09365
R2 = 0.4143
R2 = −0.002131
R2 = 0.02359
Average transcription of targets
Average transcription of targets
Transcription
Activity
Transcription
Activity
14
12
10
9
9
11
13
15
3.5
4.5
5.5
18
14
10
20
16
12
8

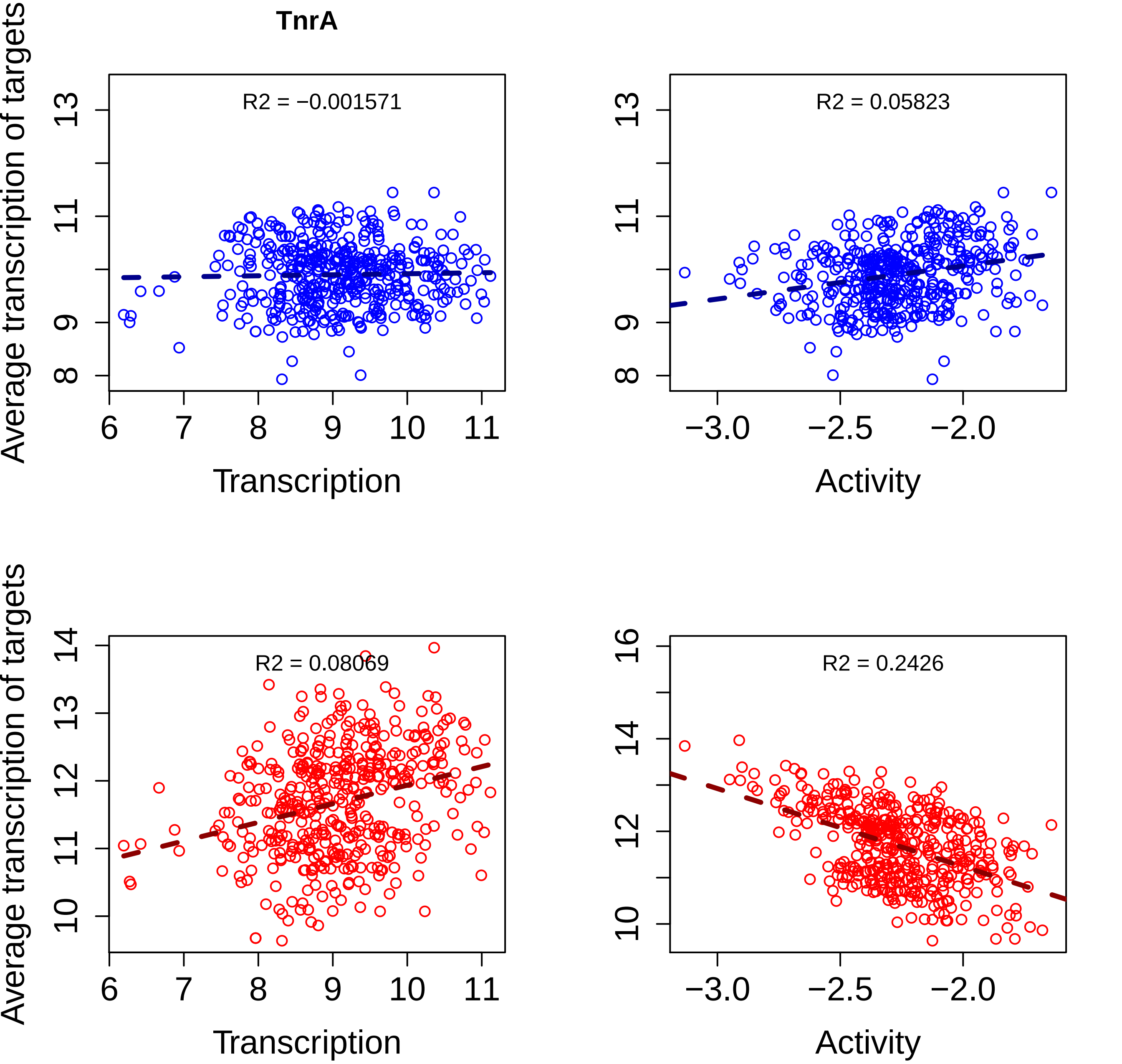

TnrA
R2 = −0.001571
R2 = 0.05823
R2 = 0.08069
R2 = 0.2426
Average transcription of targets
Transcription
Activity

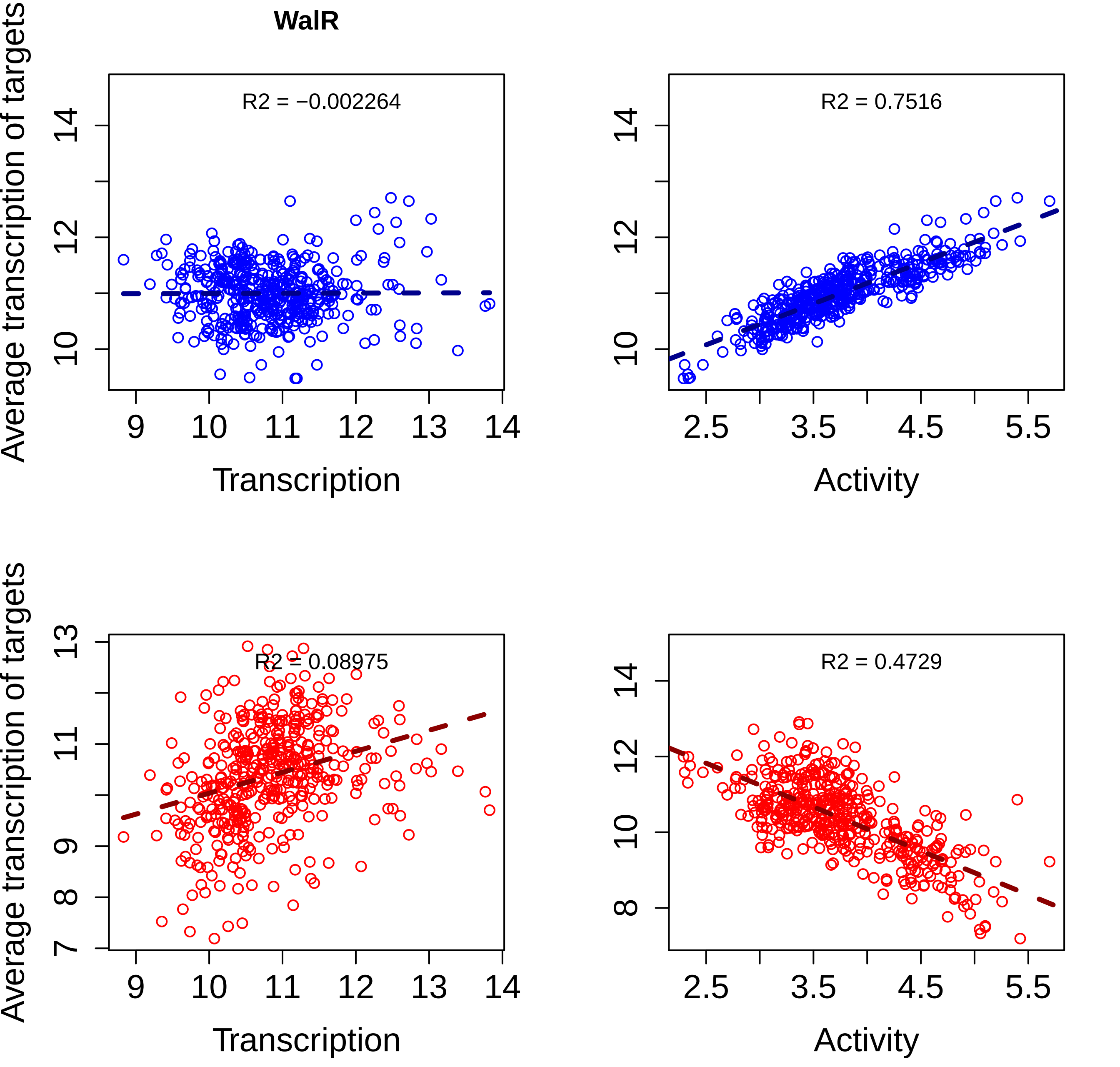

WalR
R2 = −0.002264
R2 = 0.7516
R2 = 0.08975
R2 = 0.4729
Average transcription of targets
Transcription
Activity

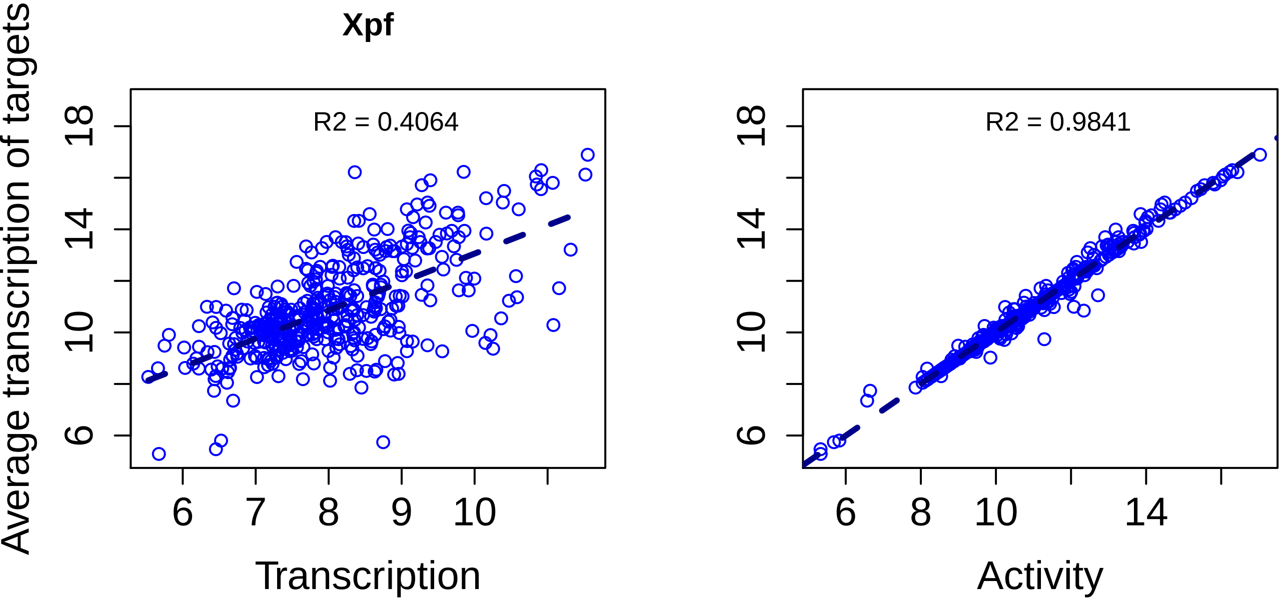

Xpf
R2 = 0.4064
R2 = 0.9841
Average transcription of targets
Transcription
Activity
6
7
8
9
10
14
18

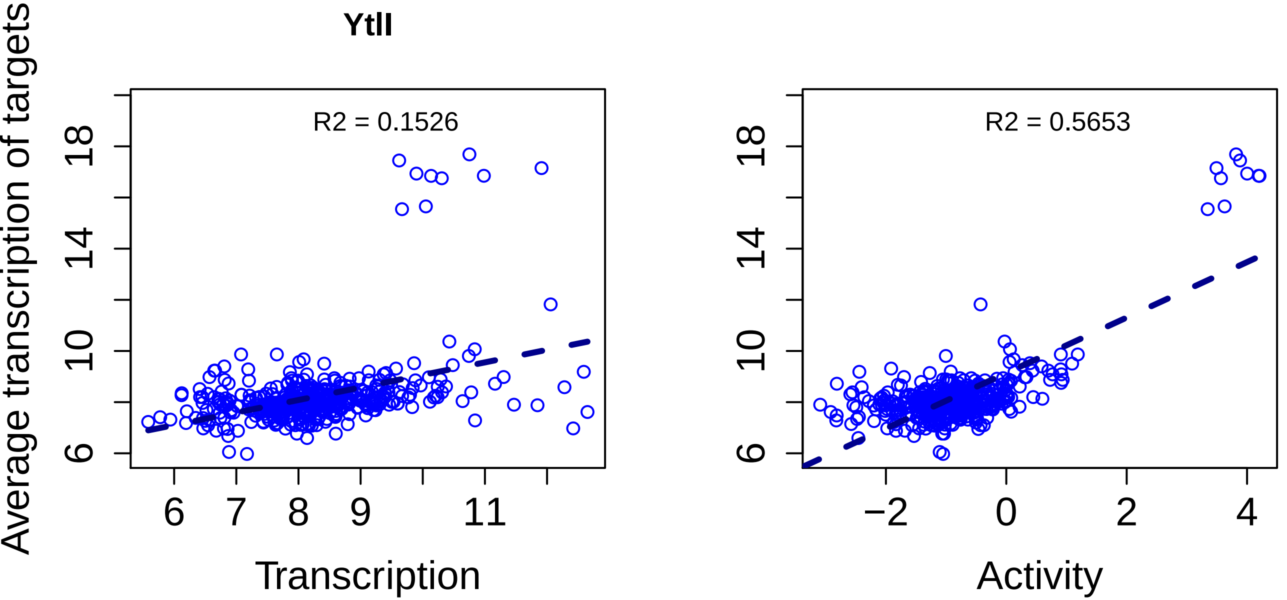

Ytll
Average transcription of targets
R2 = 0.1526
Transcription
R2 = 0.5653
Activity

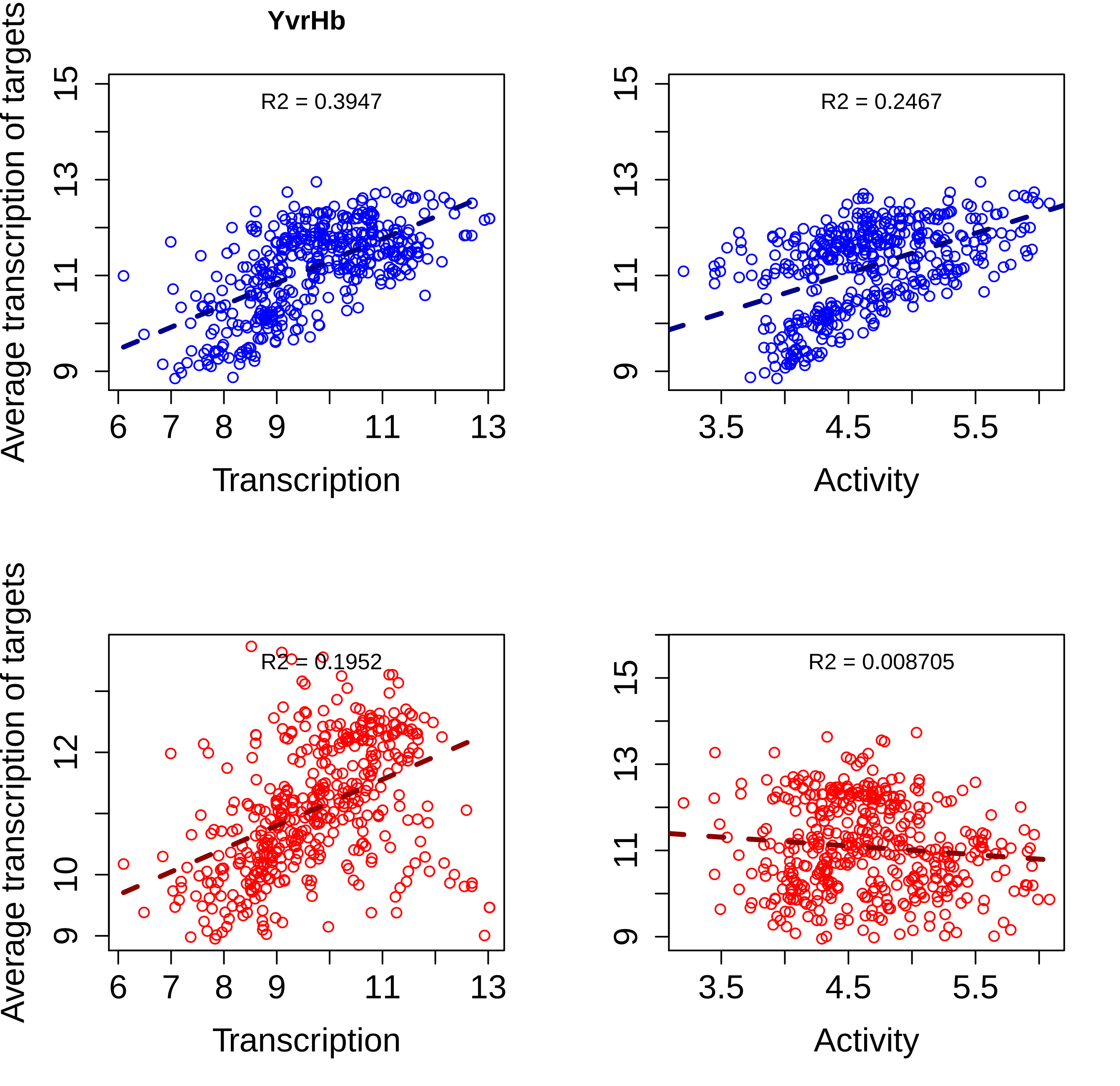

YvrHb
R2 = 0.3947
R2 = 0.2467
R2 = 0.1952
R2 = 0.008705
Average transcription of targets
Transcription
Activity
